# Functionalized Calix[4]Nanocones

**DOI:** 10.1002/anie.202512873

**Published:** 2025-07-25

**Authors:** Anika Haidisch, Frank Rominger, Michael Mastalerz

**Affiliations:** ^1^ Organisch‐Chemisches Insitut Ruprecht‐Karls‐Universität Heidelberg Im Neuenheimer Feld 270 69120 Heidelberg Germany

**Keywords:** Alkaline ion recognition, Calixarenes, Conjugated nanobelts, Selectivity

## Abstract

The interest in synthesizing conjugated nanobelts (CNBs) and nanocones has increased recently. Although a number of various structures have been achieved, almost none have additional functional groups at the peripheral rims. Based on a calix[4]arene, a functionalized calix[4]nanocone was synthesized. Due to its rigid structure in combination with methoxy groups on both the upper and the lower rim, the nanocone was tested for the binding of alkaline ions in solution by NMR measurements. The nanocone shows a high selectivity for binding Na^+^ in favor of Li^+^ and K^+^ in CDCl_3_. By comparing the nanocone with a related but structurally flexible methoxy calix[4]arene, the impact of structural rigidity on selectivity is clearly demonstrated.

## Graphical abstract

Herein we present a synthesis of a nanocone based on a calix[4]arene, which has additional functional groups at the peripheral rims. Due to its rigid structure in combination with alkoxy groups on both the upper and the lower rim, the nanocone was tested for the binding of alkaline ions in solution. It exhibits high selectivity for binding Na^+^ over Li^+^ and also K^+^ in CDCl_3_.

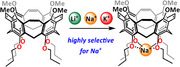

## Introduction

**Figure 1:**
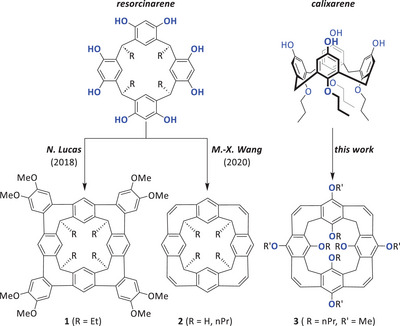
Overview of synthesized nanocones with alternating six‐ and seven‐membered rings, based on resorcinarenes (**1** ^[^ ^24^ ^]^ and **2** ^[^ ^29^ ^]^) and the work based on calix[4]arene (**3**), reported herein.

The interest in structurally distinct conjugated nanobelts (CNBs) has risen in the last decades because these structures are cut‐outs of carbon nanotubes or precursors for cyclacenes or related conjugated compounds.^[^ ^1^, ^2^, ^3^ ^]^ There are several strategies to synthesize CNBs and the like. Besides the formation of double‐stranded units by concerted cycloannulation,^[^ ^4^, ^5^, ^6^, ^7^, ^8^, ^9^, ^10^, ^11^, ^12^, ^13^ ^]^ a common strategy is the synthesis of macrocyclic precursors first, followed by nanobelt formation in the final steps.^[^ ^14^, ^15^, ^16^ ^]^ Very often, but not always, the macrocyclization is the crucial step in the multi‐step synthesis accompanied by low yields,^[^ ^10^, ^14^, ^17^ ^]^ because it is difficult to fully prevent oligomerization or polymerization. Thus, the macrocycles are usually kinetic products. It is known that reversible reactions allow the formation of thermodynamically controlled products, typically in high yields.^[^ ^18^, ^19^ ^]^ In this respect, the condensation of phenols, resorcinols, or hydroquinones with aldehydes is known to give very selectively the corresponding macrocyclic calixarenes, resorcinarenes, or pillarenes in high yields and on large scales.^[^ ^20^, ^21^, ^22^ ^]^ These high yields can rely on template effects but also on reversibility of the condensation, as has been experimentally demonstrated for calix[4]arene.^[^ ^23^ ^]^ Therefore, these kinds of macrocycles are thermodynamically controlled products and at the same time are ideal precursors for nanobelts. One of the first examples exploiting this strategy was reported by Nigel Lucas and coworkers in 2018. A resorcin[4]arene derivative was cyclodehydrogenated after Suzuki–Miyaura cross‐coupling by Scholl‐type reactions, giving the nanocone **1** in 48% yield in the final step (Figure 1, left).^[^ ^24^ ^]^ In 2020, Mei‐Xiang Wang's group presented the first synthesis of a cyclacene or beltarene precursor based on resorcinarene.^[^ ^25^ ^]^ Since then, resorcinarenes and pillarenes have been used more frequently for the synthesis of nanobelts.^[^ ^26^ ^]^ Besides carbocyclic nanobelts,^[^ ^27^ ^]^ heterocyclic nanobelts^[^ ^28^ ^]^ or nanocones (**2**, Figure 1) with seven‐ and six‐membered rings^[^ ^29^ ^]^ were realized with resorcinarenes as precursors. One of the first examples is the transformation of the hydroxyl groups of pillarenes to triflates followed by multiple Yamamoto couplings to make nanobelts with embedded five‐membered rings.^[^ ^30^ ^]^ Other macrocyclic precursors such as fluorenarenes^[^ ^31^ ^]^ or calixcarbazoles^[^ ^32^ ^]^ have been used to generate nanobelts as well. However, to the best of our knowledge, no examples based on calix[4]arenes have been described yet. Just very recently, a giant *N*‐heterocyclic nanobelt based on a calix[8]arene was introduced by Wang and coworkers.^[^ ^33^ ^]^

Here we describe the synthesis of the first nanobelts based on calix[4]arene, having a conical structure (**3**). Therefore, we call them calix[4]nanocones.^[^ ^34^, ^35^, ^36^, ^37^ ^]^ Due to the preorganized oxygens at the lower rim, it is an excellent and highly selective host for the complexation of sodium ions over lithium and potassium ions.

### Synthesis and Characterization

**Scheme 1:**
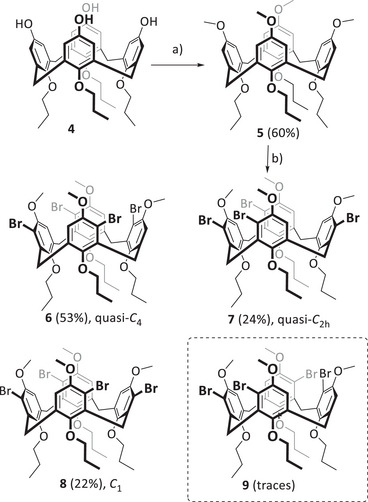
Synthesis of bromocalixarenes **6–8**. a) 1. NaOH (7.5 equiv.), H_2_O, DMSO, r.t. 30 min; 2. MeI (20 equiv.), DMSO, 40 °C, 3d. b) p‐TSA (0.3 equiv.), NBS (5 equiv.), DCM, −60 °C, 7 h. Note: The designated symmetries are for the “elusive” cone conformation.

**Figure 2:**
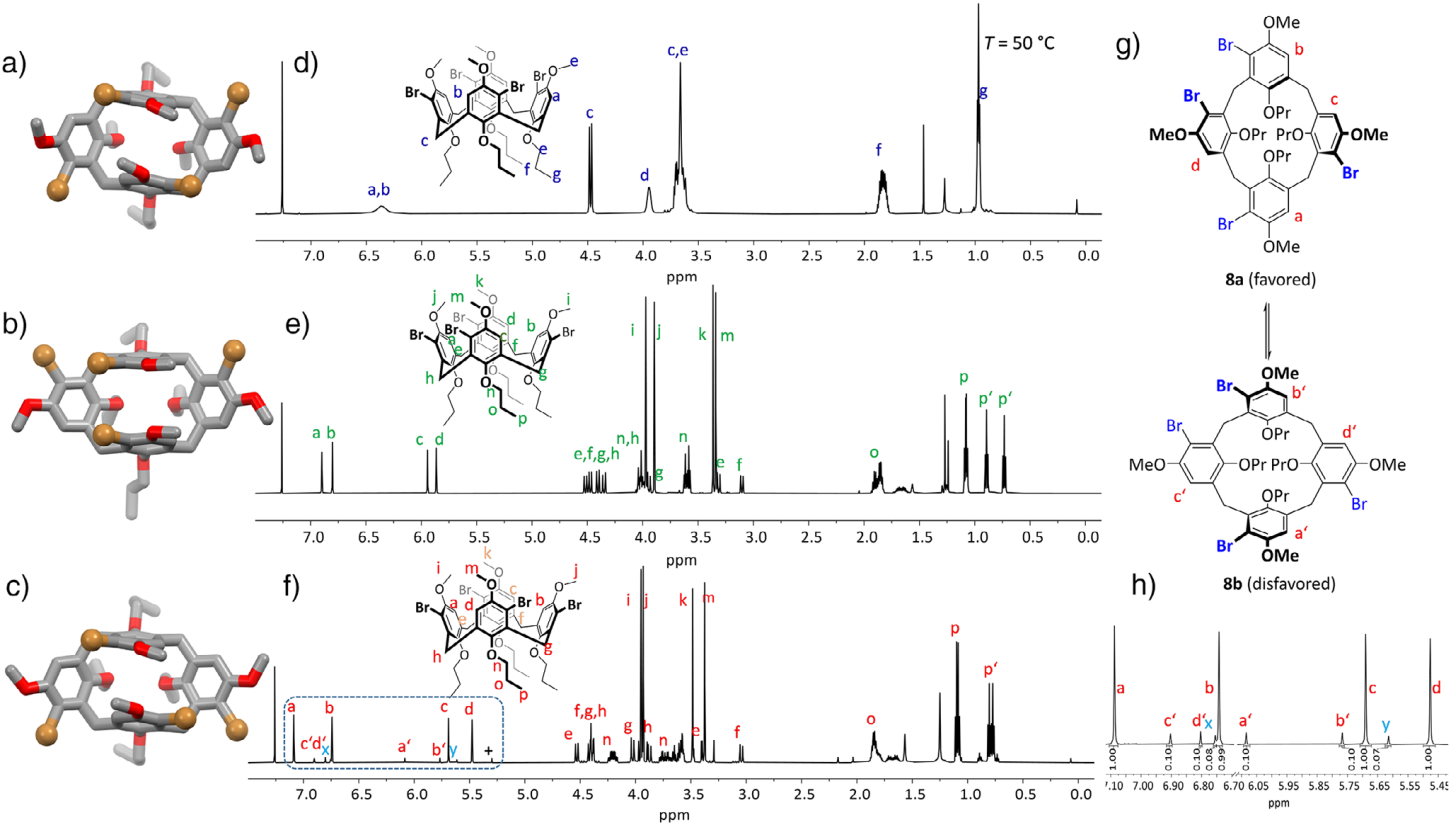
Single crystal X‐ray structures of **6** a), **7** b), and **8** c) in capped stick representation. Hydrogens are omitted for clarity. Bromine atoms are represented as balls. Depicted are only one enantiomer each of a racemic crystal. ^1^H NMR spectra (600 MHz, CDCl_3_) of **6** d), **7** e), and **8** f). d) at 323 K; (e) and (f) at 298 K. g) Equilibrium of diasteromorphic conformers of **8a** and **8b**. h) Zoomed in section of the aromatic region of ^1^H NMR spectrum of **8**. Signals *x* and *y* are proposed to be from traces of tetrabromide **9**.

The synthesis of nanocone **3** starts with tetrahydroxycalixarene **4**,^[^ ^38^ ^]^ which is fixed in the cone conformation by having four n‐propoxy chains at the lower rim (Scheme 1). The first step was the methylation of the phenolic oxygens with methyliodide to give calixarene **5** in 60% yield. Subsequent bromination with NBS by adopting a previously reported protocol on aminocalixarenes,^[^ ^39^ ^]^ gave a mixture of fourfold brominated calixarenes **6–8**, having one bromo substituent per aromatic ring. After column chromatography, these could be isolated in 53% (**6**), 24% (**7**), and 22% (**8**) yield, and their constitutional structures were unambiguously proven by NMR spectroscopy and single crystal X‐ray diffraction (Figure 2).^[^ ^40^ ^]^

C_2h_‐symmetric tetrabromocalixarene **9** was not isolated but probably has formed in traces together with **8** (see NMR discussion below). In solution as well as in the solid state, the *C*_2v_‐symmetric pinched cone conformation is preferred in comparison to a *C*_4v_‐symmetric cone conformation, and the latter is the transition state between two pinched cone conformations.^[^ ^41^ ^]^ At room temperature for most calix[4]arenes, the energy of the transition is low, and on the NMR time scale, an averaged signal set is observed.^[^ ^42^ ^]^ By temperature‐dependent NMR measurements the coalescence of the transition and thus the correlated energy can be determined. At room temperature, the signals of *C*_4_‐symmetric tetrabromo calixarene **6** are broad, revealing that interconversion between two pinched cone conformations is relatively slow on the NMR timescale (at *T_c_ =* 263 K coalescence is found, which corresponds to Δ*G* = 11.9 kcal mol^−1^). *C*_2h_‐symmetric tetrabromo calixarene **7** already shows at room temperature very sharp signals of a pinched‐cone conformation. Even at 140 °C coalescence was not reached, which shows that the transition barrier is higher than Δ*G* > 20.3 kcal mol^−1^. At first glance, the ^1^H NMR spectrum of tetrabromo compound **8** appears impure, with several small signals aside from the main peaks. It must be noted that the two *C*_1_‐symmetric pinched cone conformations are diastereoisomers, and by integration of the NMR peaks, a ratio of ∼ 10:1 toward the isomer **8a** is found, revealing that this one is thermodynamically more favored by about 1.4 kcal mol^−1^ in comparison to **8b**. This corroborates with PM6 based calculations (Δ*G*_f_ = 1.0 kcal mol^−1^). By temperature‐dependent NMR spectroscopy in CD_2_Cl_2_, a non‐linear behavior of pinched‐cone to pinched‐cone transformation (**8a** to **8b**) was found with Δ*G* ^‡^_298K _= 4.0 kcal mol^−1^. It is worth mentioning that the small peaks at 5.61 and 6.75 ppm (denoted *x* and *y*) probably stem from minor impurities of calixarene **9**, which we were not able to remove. According to the ratio of integrals, this is formed overall in less than 0.6% in the reaction.

**Scheme 2:**
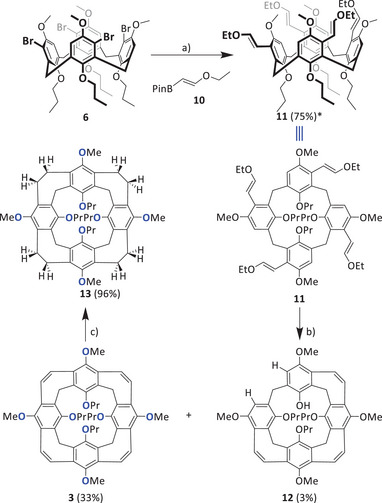
Syntheses of nanocone **3** and reduced nanocone **13**. a) **10** (20 equiv.), P^t^Bu_3_HBF_4_ (1.6 equiv.), Pd_2_dba_3_ (0.4 equiv.), THF, 1 M K_2_CO_3_, 80 °C, 20 h, Ar. b) BiOTf_3_ (0.3 equiv.), DCM (1 mg mL^−1^), 60 °C 16 h, Ar. c) Pd/C (2.5 equiv.) 60 bar H_2_ (autoclave), ethylacetate/MeOH 15:1 (v/v), 60 °C, 2d.

To synthesize calix[4]nanocone **3** (Scheme 2), the tetrabromo calixarene **6** was used in a Suzuki–Miyaura cross‐coupling with ethoxy vinyl boronic pinacol ester **10** to give **11** in 70% yield. The tetravinyl ether is very unstable and needs directly to be converted to nanocone **3** with Bi(OTf)_3_ under high dilution conditions (approx. 1 mg mL^−1^) in dry DCM.^[^ ^43^ ^]^ After purification, nanocone **3** was isolated in 33% yield, besides traces (5%) of partially closed nanocone **12** with a missing propylchain. The olefinic double bonds of nanocone **3** can be hydrogenated with Pd/C (5 wt%) to give reduced nanocone **13** in 96% yield.

**Figure 3:**
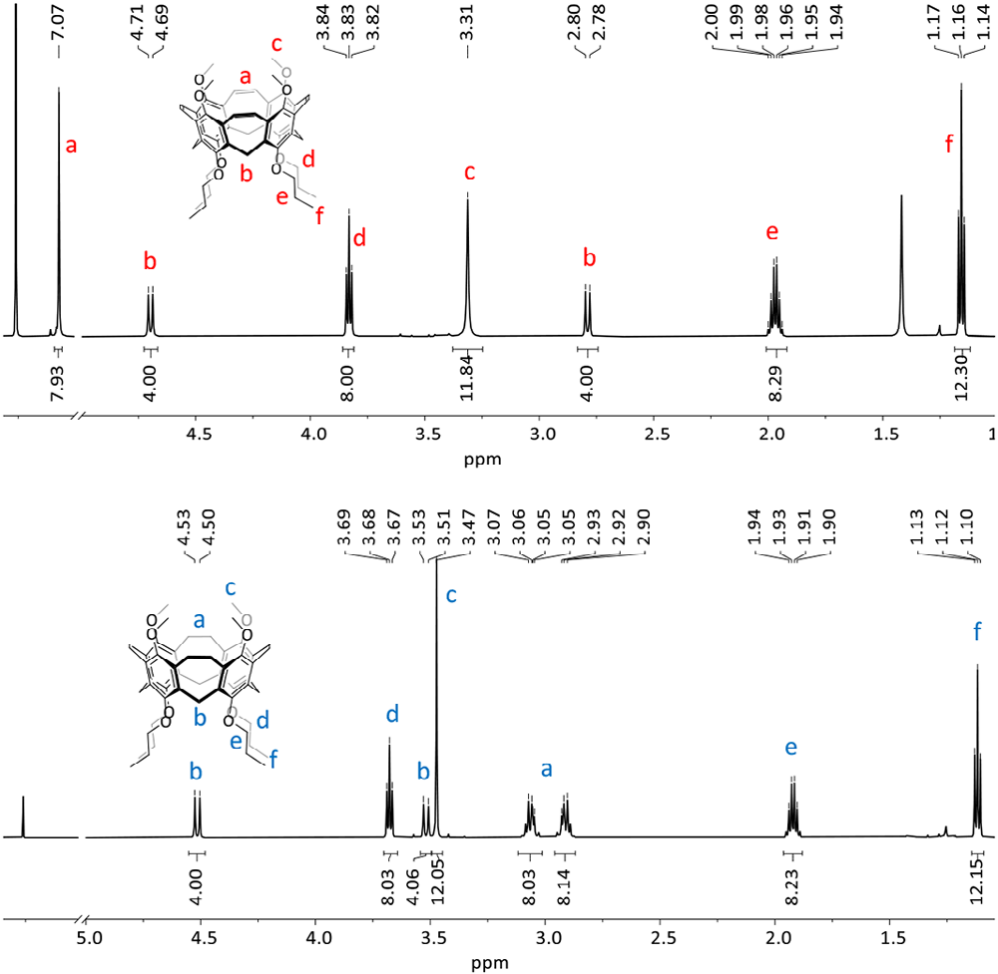
NMR spectra (600 MHz in CDCl_3_) of calix[4]nanocone **3** (top) and its reduced congener **13** (bottom). Please note that between 5.00 and 7.00 ppm the spectra are not depicted. For full spectra, see Supporting Information.

Both nanocone **3** and reduced nanocone **13** were clearly identified by MS, NMR spectroscopy, and single crystal X‐ray diffraction. Nanocone **3** shows an *m/z* peak for [M]^∙^^+^ at 808.3973 (calc. 808.3975). It is worth noting that the basis peak is found at *m/z* 831.3869, which corresponds to [M+Na]^+^, giving already a hint that the tendency of this compound toward binding of sodium ions is probably high. The reduced nanocone **13** has eight more atomic units and the basis peak at *m/z* 816.4567 fits to the calculated one at *m/z* 816.4601. Here, the corresponding [M+Na]^+^ is also found (*m/z* 839.4494), however less pronounced. Both ^1^H NMR spectra are rather simple (Figure 3). Besides the typical pattern of the propoxy chains, the bridging methylene protons are still diagnostic of the cone conformation and appear as two doublets at δ = 4.70 and 2.79 ppm, each with geminal coupling of *J =* 11.4 Hz for **3** and at δ *=* 4.52 and 3.52 ppm with *J* = 12.8 Hz for **13**. The vinylene bridge protons of **3** resonate as a singlet at δ = 7.07 ppm, whereas in the reduced nanocone **13**, the corresponding ethylene bridges give two multiplets at δ = 3.06 and 2.91 ppm with an integral 8 each for the inner and outer protons.

**Figure 4:**
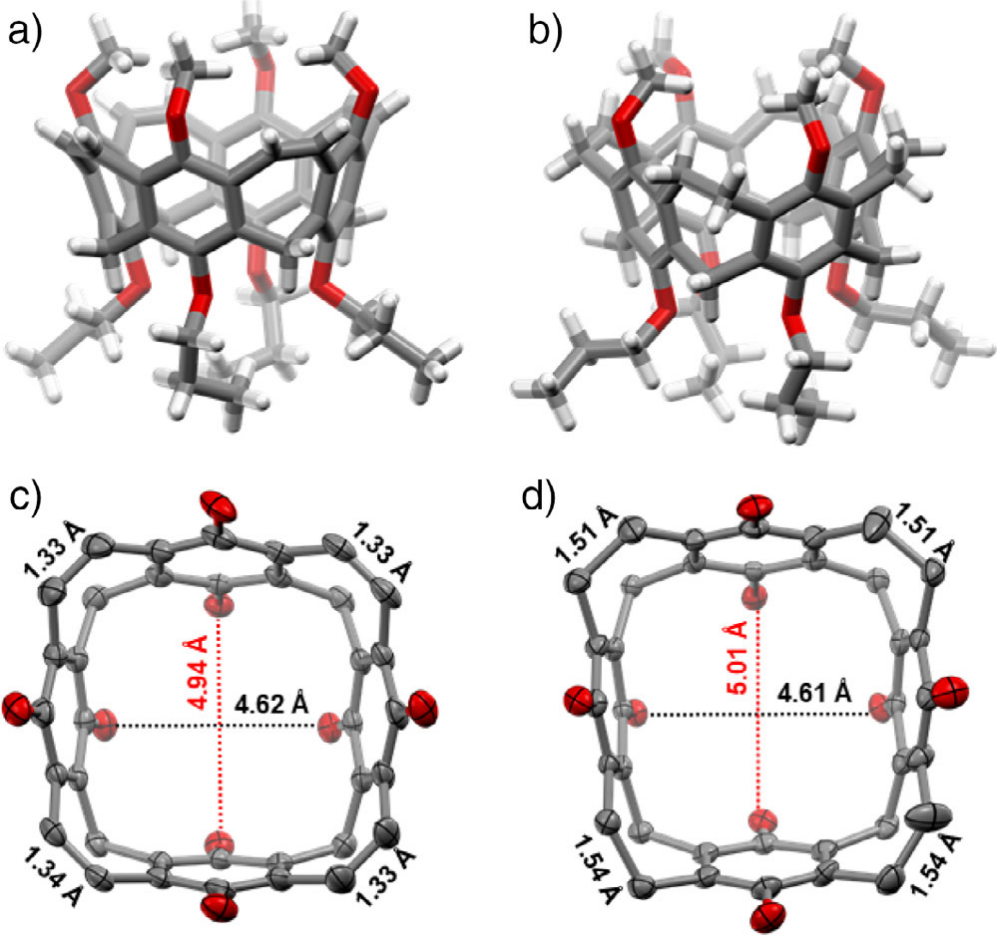
Single‐crystal X‐ray structures of nanocones **3** (a and c) and **13** (b and d). In (a) and (b) the compounds are represented as stick models; in (c) and (d) as ORTEP plots with 50% probability are shown. Here, hydrogen atoms as well as alkoxy chains were omitted for clarity.

Single crystals of nanocone **3** were grown by slow evaporation of solvent from a saturated solution of the compound in acetonitrile (Figure 4). The compound crystallizes in the monoclinic space group *C*2/c. Due to the flexible alkoxy chains, the molecule is *C*_1_‐symmetric. The vinylene bridges are 1.33 Å long, and the six‐membered rings have bonds that are non‐alternating in length, as it is typical for aromatic benzene derivatives. More interesting is the dimension of the nanocone, especially the distances of the oxygens at the upper and lower rims. Whereas the overall dimensions of the backbone are comparable to the nanocone reported by Wang and coworkers,^[^ ^29^ ^]^ the distances between distal‐oriented oxygens are at the upper rim 8.24 and 8.32 Å and at the lower rim 4.62 and 4.94 Å (average: 4.78 Å). The latter two seem ideal for complexation of lithium or sodium ions, because they are in the range of the distances calculated from van der Waals and ion radii of oxygen and the ions (*d*_(O∙∙∙Li+∙∙∙O)_ = 4.56 Å and *d*_(O∙∙∙Na+∙∙∙O_) = 5.08 Å.^[^ ^44^ ^]^

**Figure 5:**
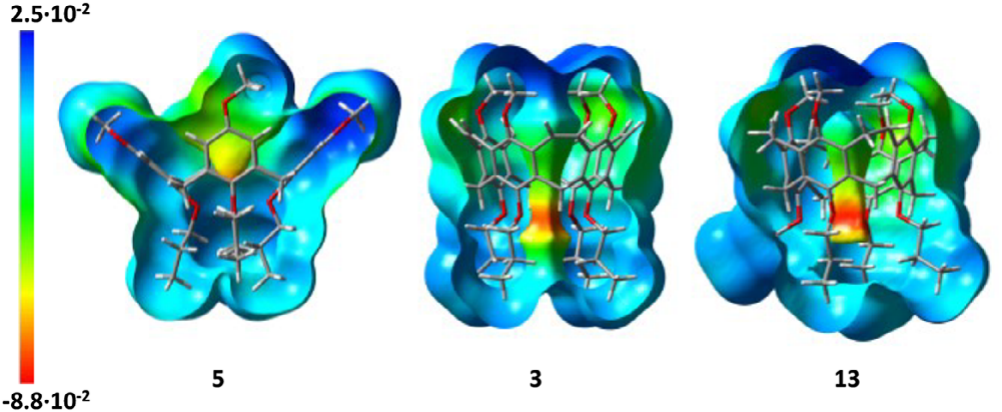
Comparison of electron density maps of calixarene **5**, nanocones **3**, and **13**. B3LYP/6–311(d,p)‐optimized structures (see Supporting Information).

By reduction of the double bonds, as done to achieve **13**, the structure becomes more flexible, leading to larger alternation of distances between opposite atoms or groups. Single crystals of reduced nanocone **13** were grown from evaporation of dichloromethane from a saturated solution, and the compound crystallized in the triclinic space group *P*‐1. The distances between distal‐oriented oxygens are at the upper rim 7.59 and 8.40 Å and at the lower rim 4.61 and 5.01 Å (average: 4.81 Å). The reduction of the vinylene bridges to ethylene bridges has been proven by their elongation from 1.33 Å in **3** to 1.51 Å in **13**. Comparison of electron density maps of calixarene **5** with nanocones **3** and **13** (Figure 5) clearly shows that in the latter two, the highest electron densities are located in the center of the plane between the oxygens at the lower rim, which should be beneficial for hosting the smaller alkaline ions.

### Complexation Studies

**Figure 6:**
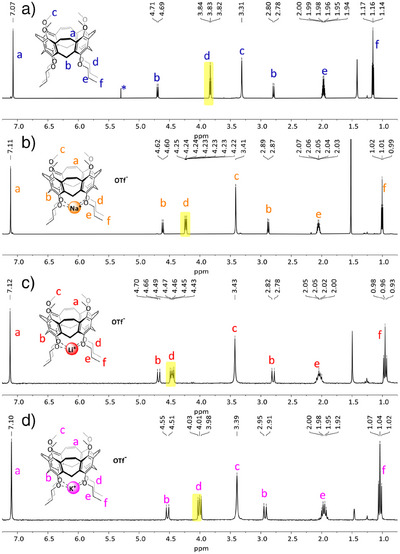
Comparison of NMR spectra (600 MHz, CDCl_3_) of nanocone **3** a) and the corresponding Na^+^‐ b) and Li^+^‐complexes c) and K^+^ complexes d). The most prominent shift during complexation is that of signal H^d^ from the methylene protons attached to the phenolic oxygens (highlighted in yellow).

We were interested, if the decrease of structural flexibility and the nearly ideal distances between the oxygens at the lower rim influence the complexation of alkaline metal ions Li^+^, Na^+^, and K^+^ also in comparison to the flexible calixarene **5**, which can in principle adopt any preferred conformation, but **3** and **13** cannot. To a solution of nanocone **3** in CDCl_3_, solid LiOTf was added and sonicated. LiOTf was fully complexed. Please note that without nanocone **3**, the triflate is nearly insoluble in CDCl_3_. The same occurs with NaOTf and KOTf. The ^1^H NMR spectra of the complexes show that all peaks of the nanocone are significantly shifted (Figure 6). The protons most affected by cation complexation are those of the methylene units attached to the oxygens of the propoxy chains (H^d^).

These shift from δ = 3.83 ppm in nanocone **3** to δ = 4.47 ppm in Li^+^‐complex Li^+^⊂**3**, δ = 4.23 ppm in Na^+^‐complex Na^+^⊂**3**, and δ = 4.01 ppm in K^+^‐complex K^+^⊂**3**. Reduced nanocone **13** behaved very similarly and dissolved any alkaline triflates in CDCl_3_ solutions thereof (for details, see Supporting Information), forming complexes Li^+^⊂**13**, Na^+^⊂**13**, and K^+^⊂**13**. From ^1^H,^19^F HOESY NMR measurements it is assumed that in solution the triflate anion is dissociated since no correlation peak of the nanocone with the triflate was found. Furthermore, another salt (NaClO_4_) gave exactly the same shifts of the methylene protons H^d^, once more suggesting that no contact ion pairs are formed.

**Figure 7:**
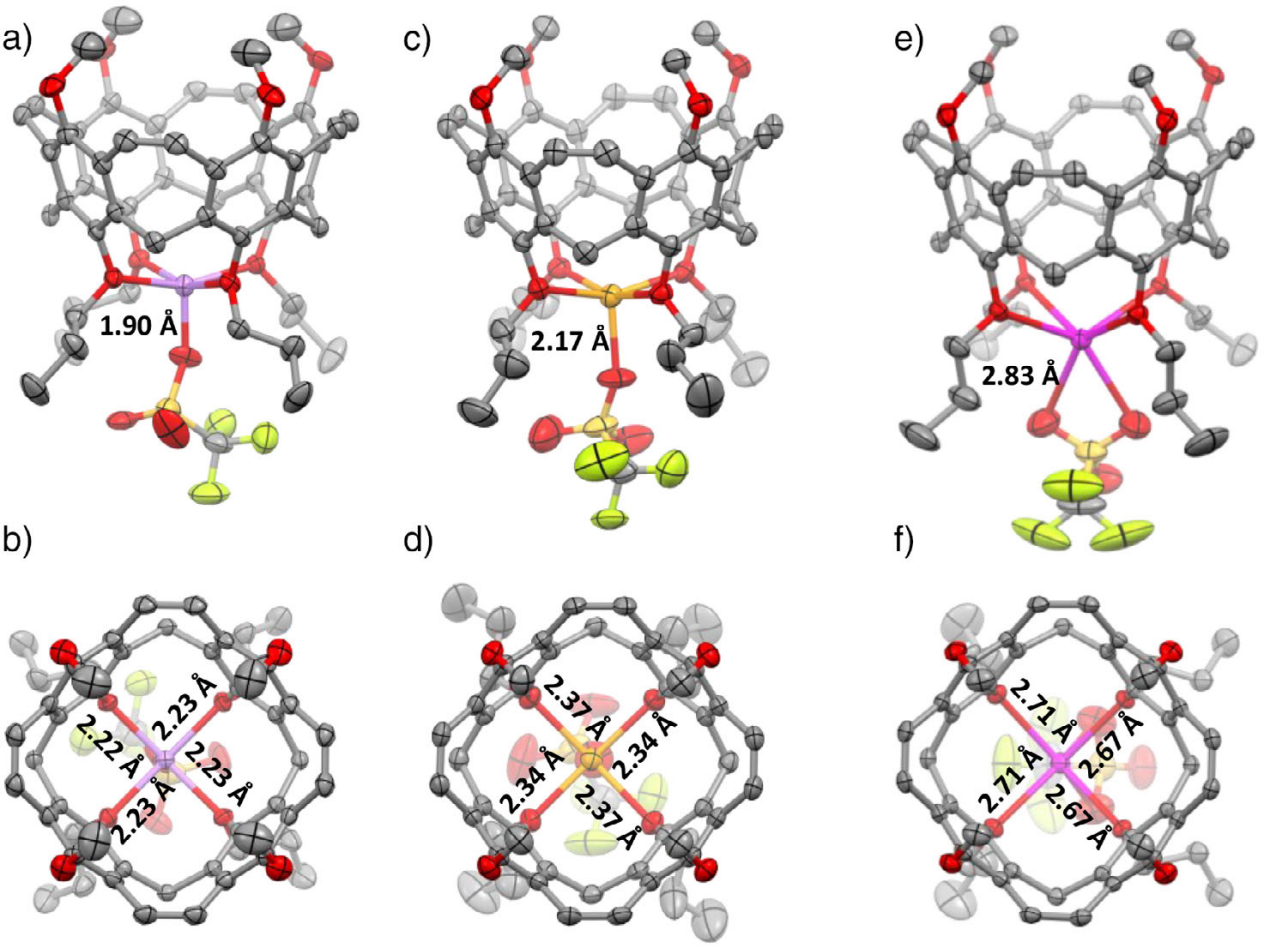
Single‐crystal structures of (Li^+^⊂**3**)OTf^−^ (a and b), (Na^+^⊂**3**)OTf^−^ (c and d), and (K^+^⊂**3**)OTf^−^ (e and f) depicted as ORTEP plots (50% probability). Hydrogen atoms and solvate molecules are omitted for clarity.

From (Li^+^⊂**3**)OTf^−^, (Na^+^⊂**3**)OTf^−^, and (K^+^⊂**3**)OTf^−^, single crystals of high quality were obtained from CDCl_3_ by evaporation of solvent (Figure 7). (Li^+^⊂**3**)OTf^−^, crystallizes in the space group *P*4/n. The lithium cation is centered between the four oxygens of the propoxy chains of the lower rim at a distance of *d* = 2.22–2.23 Å. The lithium is placed only 0.39 Å below the plane that the four oxygen atoms build. The triflate is bound as a fifth ligand to the lithium at a distance of 1.90 Å. (Na^+^⊂**3**)OTf^−^ crystallizes in the space group *P*21/c. Here, the distance between the alkaline metal ion and the oxygens is *d* = 2.34 and 2.37 Å somewhat larger, and the distance to the triflate is with 2.17 Å also larger. Most important, the sodium cation is found at 0.41 Å below the plane that is formed by the oxygens. This is only somewhat lower than for the lithium, so it is assumed that both cations fit well to the preorganized host molecules. This situation is different for the larger potassium ion. (K^+^⊂**3**)OTf^−^ crystallized in the spacegroup *P*nma, the distance between K^+^ and the oxygens is with *d *= 2.67 and 2.71 Å substantially larger than in the two other complexes. The cation is placed 1.06 Å below the plane that is formed by the oxygen atoms, which is substantially longer than for the other cations. The triflate is acting as a bifurcating ligand with a distance of 2.83 Å between the two oxygens. (Li^+^⊂1**3**)OTf^−^ shows very similar distances (*d*_Li∙∙∙O _= 2.23 Å) compared to (Li^+^⊂**3**)OTf^−^ (for details see Supporting Information).

We were interested in how well and selectively the nanocones bind alkaline cations in solution and how important the rigidity and pre‐organization of the alkoxy oxygens at the lower rim is.^[^ ^45^ ^]^ Although the used alkaline triflates are nearly insoluble in CDCl_3_ (all < 0.015 mmolL^−1^), as soon as nanocone **3** is present, the salts dissolve immediately and complexes Li^+^⊂**3**, Na^+^⊂**3**, and K^+^⊂**3** are formed quantitatively. By adding understoichiometric amounts of triflates, we tried to determine the association constants, but again, all triflate is bound within the nanocone **3**, assuming that the binding constants K_a_,_CDCl3_ exceeded >10^5^ M^−1^. Reduced nanocone **13** behaved very much the same and was therefore not further investigated in detail.

The electronic situation of more flexible calixarene **5** is comparable to nanocones **3** and **13** and gives insight about the impact of ligand preorganization.^[^ ^46^ ^]^ Here, although triflates were added in large excesses, the formation of Li^+^⊂**5** never exceeded 95%, and Na^+^⊂**5** never exceeded 84%. K^+^ is not taken up at all by calixarene **5**.

In the experiments with understoichiometric amounts of salt, two well‐separated sets of peaks were found in the NMR spectra, suggesting that the exchange is slow on the NMR time scale. This allowed us to clearly distinguish Li^+^⊂**3**, Na^+^⊂**3**, and K^+^⊂**3** and nanocone **3** and quantify the equilibrium concentration of those compounds. Therefore, 1:1:0.8 mixtures of two different alkaline triflates and the nanocone **3** were mixed, and NMR spectra were measured until they reached equilibrium (no further change in relation of integrals). In the mixture of LiOTf, NaOTf, and nanocone **3**, exclusively Na^+^ was taken up, which means that this one is at least 100 times better bound than Li^+^. A similarly observation toward the Na^+^ in favor of K^+^ was made with their triflates: Only traces of K^+^⊂**3** were found in the ^1^H NMR spectrum with integrals below the detection limit (< 5%). When LiOTf, KOTf, and nanocone **3** were mixed 1:1:0.8, the equilibrium showed a ratio of 45% of Li^+^⊂**3** and 55% of K^+^⊂**3**.

The advantage of ligand preorganization to the binding of Na^+^ was confirmed by competition experiments of CDCl_3_ solutions of pure (Na^+^⊂**3**)OTf^−^, treated with defined amounts of calix[4]arene **5**. Here an equilibrium constant of *K*_e _= 2.2∙10^4^ in favor of Na^+^⊂**3** was determined. We also compared the nanocone **3** with some of the best ligands for Na^+^ ions,^[^ ^45^ ^]^ namely 18‐crown‐6^[^ ^47^, ^48^ ^]^ and Lehn's cryptand 2.2.1.^[^ ^49^, ^50^ ^]^ When adding stoichiometric amounts of cryptand 2.2.1, all Na^+^ is immediately removed, and only signals of free nanocone **3** are observed in the ^1^H NMR spectrum and none of the complex Na^+^⊂**3**, clearly demonstrating that the cryptand has an association constant several orders of magnitude higher than the nanocone **3**. With 18‐crown‐6, the nanocone binds Na^+^ better with *K*_e_ = 3.2. By the knowledge of the association constant of 18‐crown‐6 for Na^+^ in CDCl_3_ (*K*_a_ = 1.3·10^6^ M^−1^)^[^ ^51^ ^]^ this results in a *K*_a_ = 4.2·10^6^ M^−1^ for binding sodium by nanocone **3**.

THF is known to be a good solvent/ligand for alkaline cations. Therefore, complexation studies in THF‐d8 were performed too. With the exception of KOTf, the other two salts were very good soluble. Nanocone **3** binds Na^+^ with an association constant of 2.5·10^3^ M^−1^ and again, two signal sets are found in the NMR spectrum, revealing a slow exchange on the NMR time scale. Although KOTf is badly soluble, binding is confirmed by two signal sets and K^+^ is bound only with *K*_a_ ∼ 150 M^−1^. With LiOTf in THF, neither a second signal set nor a significant shift of NMR signals was observed, suggesting that either Li^+^ in THF is weakly bound or its exchange is very fast on the NMR time scale at room temperature. Even if an NMR sample with 100 equiv. of LiOTf was cooled down to −100 °C, no splitting of signal sets was observed. Nevertheless, we did an NMR titration with LiOTf, treated as if it binds by fast exchange. The change in shift over the course of titrations is very small (Δδ_max_(H^a^) = +0.002 ppm) and in the range of the standard deviation (σ_δ(Ha)_ = ± 0.001 ppm), which is why the discussion of the results needs to be understood as a trend only. Within six NMR titrations of two series (each measured three times), a mean binding constant of *K*_a_ = 15.2 M^−1 ^± 13.5 was estimated. The large error is representative of the large uncertainty of the binding constant, resulting in a conservatively estimated selectivity of *S*_Na/K _> 165). Calixarene **5** in contrast, binds all cations (*K*_ass,Li_ < 10^−2^ M^−1^; *K*_ass,Na_ = 1.35 M^−1^, *K*_ass,K_ < 10^−2^ M^−1^) very weakly in THF‐d8, once more emphasizing the beneficial pre‐orientation of the oxygens for the complexation process as it is typically found for spherands and cryptospherands.^[^ ^46^ ^]^

## Conclusion

Based on a calix[4]arene a methoxy functionalized nanocone **3** was synthesized. The methoxy groups at the lower rim in combination with the structural rigidity are ideally oriented to bind alkaline metal cations. In chloroform, Li^+^, Na^+^ and K^+^ are immediately complexed from their triflates of the solid phase. The determined association constant for Na^+^ (*K*_a_ = 4.2 10^6^ M^−1^) is comparable to that of 18‐crown‐6 (*K*_a_ = 1.3·10^6^ M^−1^) but clearly lower than reported for Lehn's cryptand 2.2.1, (*K*_a_ = 10^13^ M^−1^) or Cram's cryptahemispherands but in the same range as von Delius’ ortho‐ester cryptands. ^[^ ^44^, ^46^, ^52^, ^53^ ^]^ The additional methoxy groups at the upper rim allow the nanocones to be functionalized in a way so that they can be immobilized on a stationary phase to allow the separation of Na^+^ from Li^+^ in a heterogenous manner. Furthermore, the functional groups will be the fundament to use nanocone **3** to make larger polycyclic aromatic compounds with conical shape.

## Supporting Information

The authors have cited additional references ^[^ ^54^, ^55^, ^56^, ^57^, ^58^, ^59^, ^60^, ^61^, ^62^, ^63^, ^64^, ^65^, ^66^, ^67^, ^68^, ^69^, ^70^, ^71^, ^72^, ^73^, ^74^, ^75^, ^76^, ^77^, ^78^, ^79^, ^80^, ^81^, ^82^, ^83^, ^84^, ^85^, ^86^, ^87^, ^88^, ^89^, ^90^, ^91^, ^92^, ^93^, ^94^, ^95^, ^96^ ^]^ within the Supporting Information

## Conflict of Interests

The authors declare no conflict of interest.

## Supporting information



Supporting Information

Supporting Information

## Data Availability

The data that support the findings of this study are available from the corresponding author upon reasonable request.
