# Supplementary material for: Functionalized Calix[4]Nanocones
Source: Angew Chem Int Ed Engl. 2025 Jul 25;64(37):e202512873. doi: 10.1002/anie.202512873 (PMC12416480; doi:10.1002/anie.202512873)
Supplement: Supplementary file 1 — Supporting Information [file ANIE-64-e202512873-s001.docx]

Functionalized Calix[4]nanocones

A. Haidisch, Dr. F. Rominger, Prof. Dr. M. Mastalerz*

Organisch-Chemisches Institut

Ruprecht-Karls-Universität Heidelberg

Im Neuenheimer Feld 270, 69120 Heidelberg (Germany)

Email: [michael.mastalerz@oci.uni-heidelberg.de](mailto:michael.mastalerz@oci.uni-heidelberg.de)

-Supporting Information-

Table of Contents

1 General remarks S3

2 Synthesis S5

3 NMR Spectroscopy S15

4 Mass Spectrometry S51

5 FTIR Spectrometry S55

6 UV/VIS Spectra S59

7 Crystallographic data S61

8 Pinched cone interconversion of bromocalixarenes **6**, **7** and **8** S73

9 NMR titration experiments S78

9.1 General Procedure S78

9.2 Titration of calixarene **5** with alkali triflates S78

9.3 Titration of nanocone **3** with alkali triflates S84

9.4 Competition reaction between salts and nanocone **3** S110

9.5 Na^+^⸦**3** in CDCl_3_ and CDCl_3_·D_2_O S115

9.6 Nanocone **3** NaClO_4_ and NaOTf S116

9.7 NMR spectra of redcone **13** and alkali triflates S117 9.8 Titration between calixarene **5** and nanocone **3** S118

10 Calculations S121

11 References S130

1. **General Remarks**

Flash column chromatography was performed using Silica Gel with a particle size of 0.040-0.063 mm. (Macherey-Nagel & Co. KG, Düren). Light Petroleum ether and ethyl acetate or mixtures of them were used as eluents. The eluent was chosen by thin layer chromatography (TLC), whereby the *R*_f_-value was set under 0.4 for the fastest eluting spot and then used for the corresponding flash column. For thin layer chromatography fluorescent labelled silica coated aluminium plates (60 F254, Merck) were used. The spots were detected by UV-light irradiation with wavelengths of λ = 254 and 366 nm. High performance liquid chromatography was performed on a Shimadzu Prominence LC-20 NP-rec.-HPLC preparative pump unit, CBM-20A communication bus module, SPD-M20A diode array detector, FCV-20AH2 valve unit and a Restek ultra silica 5 μm (250 x 21.2 mm) normal phase column. NMR-spectra (^1^H, ^13^C, ^19^F- 2-D Spectra) were recorded in CDCl_3,_ dimethylsulfoxide-d_6_, acetone-d_6,_ MeOD or tetrachlorethan-d_2_ using a Bruker Avance III 300 (^1^H: 300 MHz), Fourier 300 (^1^H: 300 MHz), Bruker Avance III 400 (^1^H: 400 MHz, ^13^C: 101 MHz), Avance III 500 (^1^H: 500 MHz, 5 mm TBO BB {^1^H/^19^F} ATM z-Gradient for Temperature measurement between -100°C to +150°C) Brucker Avance III 600 (^1^H: 600 MHz, ^13^C: 151 MHz) spectrometer or Bruker Avance III 700 (^1^H: 700 MHz) at 295 K, unless otherwise mentioned. Chemical shifts are reported in parts per million (ppm) relative to traces of the used solvents in the corresponding fully deuterated solvent, unless otherwise mentioned.^[S^[^1^](#_ENREF_1)^]^ Elemental analyses to determine the amount of C, N and H were performed by the Microanalytical Laboratory at the University of Heidelberg using an vario MICRO cube. Melting points (not corrected) were determined using a Büchi^©^ Melting Point B-565 apparatus. The melting points were measured in open glass capillaries. All mass spectra were recorded by the MS-Laboratory of the Chemical Institutes of the University of Heidelberg under supervision of Dr. Jürgen Gross. MALDI-TOF-mass spectra were carried out on a Bruker AutoFlex Speed time of-flight or a Bruker timsTOFfleX with DCTB (trans-2-[3-(4-tert-butylphenyl)-2-methyl-2-propenylidene]malo-nonitrile) as matrix ESI(+)-mass spectra were carried out on a **Bruker ApexQe** Fourier transform ion cyclotron resonance. IR spectra were recorded neat on a Fourier transform spectrophotometer (Bruker Lumos) equipped with a Zn/Se ATR crystal or in a potassium bromide matrix. The signal intensity was described with: s (strong), m (medium), w (weak) and br (broad). Absorption spectra were recorded on Jasco UV-VIS V-730 spectrophotometer. For the calculation of molar extinction coefficients at least five solutions of different concentration were prepared by standard addition method. Cyclovoltammetry (CV) and Differential Pulse Voltammetry (DPV) spectra were recorded on a Metrohm Autolab PGSTAT101 potentiostat using a Pt working electrode (0.78 mm^2^), a Pt counter electrode and an Ag/Ag+ pseudo reference electrode in degassed HPLC-grade dichloromethane. As an internal standard ferrocene was used for calibration. Crystal structure analysis was performed on a Bruker APEX II Quazar (λ_MoKα_ = 0.71073 Å) or a Stoe Stadivari (λ_CuKα =_1.54186 Å) diffractometer. Intensities were corrected for Lorentz and polarization effects, an empirical scaling and absorption correction was applied using SADABS ^[S^[^2^](#_ENREF_2)^]^ or X-Area LANA 1.83.8.0 (STOE, 2020) based on the Laue symmetry of the reciprocal space (μ, 𝑇𝑚𝑖𝑛 and 𝑇𝑚𝑎𝑥) structure solved with SHELXT-2014^[S^[^3^](#_ENREF_3)^]^ and refined against F^2^ with a Full-matrix least-squares algorithm using the SHELXL-2018/3^[S^[^4^](#_ENREF_4)^]^ software. Hydrogen atoms were input at calculated positions and refined with a riding model. When found necessary, disordered groups and/or solvent molecules were subjected to suitable geometry and adp restraints and/or constraints. This paper was written with Microsoft Word 2016. Chemical formulas were drawn using ChemDraw Professional 20.1.1 or and ChemDraw 3D 20.1.1 by Cambridgesoft. NMR spectra were integrated and processed using MestReNova (14.2.2). Mass Spectra, UV/vis spectra and fluorescence Spectra were processed using OriginPro2022. Exact masses were calculated using mMass version 5.5.0.

All quantum chemical calculations were performed by employing the Gaussian16 ^[S^[^5^](#_ENREF_5)^]^ program package. The theoretical approach is based on Kohn-Scham density functional methodologies ^[S^[^6-9^](#_ENREF_6)^]^ using the B3LYP ^[S^[^10-12^](#_ENREF_10)^][S^[^13^](#_ENREF_13)^]^ functional. As basis set the triple-ζ-bases 6-311G(d,p) ^[S^[^14-17^](#_ENREF_14)^]^ was used. The geometries were pre-optimized using the semi-empirical PM6 method ^[S^[^18^](#_ENREF_18)^]^. Ground states were confirmed by using frequency calculations to not exhibit any imaginary frequency. NICS(0) and NICS(1) values were calculated from the optimized geometries by adding a ghost atom in the centroid or 1 Å above/below of the corresponding ring and performing a single point calculation based on Hartree Fock ^[S19-S25]^ methods using the augmented double ζ-basis 6 31+G(d) ^[S^[^14-17^](#_ENREF_14)^]^ with the GIAO^[S26-S30]^ method. The electronic potential map was calculated by using the comment prop=EFG ^[S^[^31-32^](#_ENREF_31)^]^ and generating the potential and density cube files.

Precursor **4** was synthesized according to a literature known procedure. ^[S33-S37]^

1. **Synthesis**

2.1 5,11,17,23-Tetramethoxy-25,26,27,28-tetrapropoxycalix[4]arene **5**

Calixarene **4** (8 g, 12.1 mmol) was dissolved in dimethylsulfoxide (120mL) and sodium hydroxide (3.7 g in 8 mL water) was added. The solution was stirred for 30 min while the yellow solution turns into dark brown. Methyl iodide (13.6 mL, 243.6 mmol) was added and the solution was stirred for 3d at 40 °C. After cooling to room temperature, NEt_3_ (40 mL), dichloromethane (100 mL) and water (100 mL) were added. The layers were separated and the aqueous layer was extracted with dichloromethane (2 × 50 mL). The combined organic layers were washed with water (100 mL) and brine (100 mL) and dried over MgSO_4_. After evaporation of the solvent in vacuo, separation by flash column chromatography (SiO_2_, ethyl acetate/petroleum ether 1:5, *R*_f_ = 0.52) gave **5** as colourless crystals (4.8 g, 55% yield). **M.p. =** 162 °C (dec.), **^1^H NMR (600 MHz, CDCl_3_)** *δ*= 6.19 (s, 8H, Ar-*H*), 4.41 (d, *J* = 13.3 Hz, 4H, Ar-CH_2_-Ar), 3.77 (t, *J* = 7.6 Hz, 8H, O-CH_2_-), 3.56 (s, 12H, OCH_3_), 3.05 (d, *J* = 13.4 Hz, 4H, Ar-CH_2_-Ar), 1.88 (h, *J* = 7.5 Hz, 8H, CH_2_-CH_2_-CH_3_), 0.97 (t, *J* = 7.5 Hz, 12H, CH_2_-CH2-CH_3_) ppm.**^13^C NMR (151 MHz, CDCl_3_)** *δ* = 154.3 (Ar*C*-OMe), 151.0 (Ar*C*-OC_3_H_7_), 136.0 (Ar*C*-CH_2_-Ar*C*), 113.3 (Ar*C*-H), 77.1 (O-*C*H_2_-), 55.4 (O*C*H_3_), 31.8 (Ar-*C*H_2_-Ar), 23.4 (CH_2_-*C*H_2_-CH_3_), 10.6 (CH_2_-CH_2_-*C*H_3_) ppm. **IR (neat, ATR):** *ṽ* = 2993 (vw), 2959 (w), 2934 (w), 2920 (w), 2874 (w), 2835 (w), 1718 (vw), 1601 (m), 1591 (m), 1479 (m), 1466 (s), 1435 (m), 1383 (m), 1317 (w), 1298 (w), 1279 (w), 1258 (w), 1242 (w), 1229 (m), 1209 (vs), 1165 (w), 1144 (s), 1107 (vw), 1055 (vs), 1036 (m), 1005 (s), 966 (m), 959 (m), 934 (w), 922 (vw), 912 (vw), 887 (w), 849 (s), 820 (w), 787 (w), 756 (w), 735 (w), 712 (w), 683 (vw), 669 (vw), 613 (vw) cm^-1^. **UV/VIS** (CH_2_Cl_2_): *λ* = 287 nm. **CV** (CH_2_Cl_2_, Bu_4_NPF_6_, E vs. Fc/Fc^+^): *E*_1/2ox1_ = 0.68 eV; *E*_1/2ox2_ = 0.91 V. **HRMS (ESI+, CH_2_Cl_2_/MeOH):** *m/z* calculated for [M+Na]^+^ C_44_H_56_O_8_Na^+^: 735.3868 found: 735.3888. **Elemental Analysis**: Calcd. for C_44_H_56_O_8_ (%): C 74.13, H 7.92, found: C 73.89, H 8.04.

2.2 Bromomethoxypropoxycalixarenes **6, 7, 8**

In three reaction vessels, calix[4]arene **5** (3 × 600 mg, 841 µmol) and para-toluenesulfonic acid (3 × 48 mg, 25.0 mmol) were suspended in dichloromethane (36 mL each) and cooled to -61 °C. N-bromosuccinimide (3 × 749 mg, 4.2 mmol) was added and the mixtures were stirred for 6.5 h at -61 °C. The reaction mixture was allowed to reach room temperature and stirred for additional 30 min. The solutions were combined and water was added (60 mL). The phases were separated and the organic layer washed with water (2 × 60 mL) and dried over MgSO_4_. Purification by flash column chromatography (SiO_2_, ethyl acetate/petroleum ether 1:10) gave:

1^st^ fraction (*R*_f_ = 0.46): 1.4 g (53%) of 5,11,17,23-tetramethoxy-4,10,16,22-tetrabromotetra-propoxycalix[4]arene **6** as colorless solid.

**M.p.:** 222-224 °C. **^1^H NMR (700 MHz, CDCl_3_)** *δ =* 6.36 (s, 4H, Ar-*_H_*), 4.47 (d, *J* = 14.1 Hz, 4H, Ar-*CH_2_*-Ar), 3.94 (dd, *J* = 12.4, 6.4 Hz, 4H, O-C*H*_2_-), 3.76 – 3.57 (m, 20H: (4H, O-C*_H_*_2_-), (12H, OC*_H_*_3_), (4H, Ar-C*H*_2_-Ar), 1.84 (m, 8H, CH_2_-C*H*_2_-CH_3_), 0.97 (t, *J* = 7.5 Hz, 12H, CH_2_-CH_2_-C*H*_3_) ppm. **^13^C NMR (176 MHz, CDCl_3_)** *δ* = 151.8 (Ar*C*-OPr), 151.0 (Ar*C*-OMe), 135.3 (Ar*C*-CH_2_-*C*Ar), 132.8 (A*rC*-CH_2_-*C*Ar), 114.0 (Ar*C*-H/Br), 111.9 (Ar*C*-H/Br), 77.3 (O-*C*H_2_-), 56.6 (O*C*H_3_), 31.9 (Ar-*C*H_2_-Ar), 23.0 (CH_2_-*C*H_2_-CH_3_), 10.4 (CH_2_-CH_2_-C*H*_3_) ppm. Note: Due to peak broadening caused by the PCI the carbon signal for ArC-H were not detectable. **IR (neat, ATR):** *ṽ* = 2961 (m), 2935 (m), 2874 (w), 2839 (w), 1583 (m), 1454 (s), 1435 (s), 1400 (m), 1381 (s), 1342 (w), 1317 (m), 1298 (m), 1290 (m), 1273 (w), 1232 (vs), 1188 (m), 1146 (m), 1086 (vs), 1067 (vs), 1036 (m), 1001 (s), 968 (s), 945 (m), 933 (s), 887 (m), 864 (m), 843 (m), 831 (m), 804 (w), 785 (m), 773 (m), 733 (m), 710 (w), 698 (w), 685 (w), 660 (w), 640 (m), 631 (m), 609 (m) cm^-1^. **UV/VIS** (CH_2_Cl_2_): *λ* = 296 nm. **HRMS (MALDI-TOF+, DCTB):** m/z calcd. for [M]^+^ C_44_H_52_Br_4_O_8_^+^: 1024.0396, found: 1024.0390, *m/z* calcd. for [M+Na]^+^ C_44_H_52_Br_4_O_8_Na^+^: 1047.0288, found: 1047.0293, *m/z* calcd. for [M+K]^+^ C_44_H_52_Br_4_O_8_K^+^: 1063.0022, found: 1063.0028. **Elemental Analysis**: Calcd. for C_44_H_52_O_8_Br_4_ (%): C 51.38, H 5.10, found: C 51.62, H 5.10.

2^nd^ fraction (*R*_f_ = 0.29): 566 mg (24%) of 5,11,17,23-tetramethoxy-4,10,16,24-tetrabromo-25,26,27,28-tetrapropoxycalix[4]arene **8** as yellow solid.

**M.p:** 69°C wax like. **^1^H NMR (600 MHz, CDCl_3_)** *δ* = 7.09 (s, 1H, H-5), 6.74 (s, 1H, H-17), 5.69 (s, 1H, H-12), 5.48 (s, 1H, H-26), 4.53 (d, *J* = 13.7 Hz, 1H, H-8), 4.44 – 4.37 (m, 3H, H-15, H-22, H-1), 4.20 (dtd, *J* = 19.4, 11.9, 5.1 Hz, 2H, C*H*_2_-*C*H_2_-CH_3_), 4.03 (d, *J* = 14.6 Hz, 1H, H-22), 3.95 (s, 3H, H-29), 3.93 (s, 3H, H-31), 3.87 (d, *J* = 14.1 Hz, H-1), 3.73 (dtd, *J* = 28.6, 11.7, 5.1 Hz, 2H, C*H*_2_-*C*H_2_-CH_3_), 3.66 – 3.56 (m, 5H, O-C*H*_2_-), 3.48 (s, 3H, H-30), 3.40 (d, *J* = 5.8 Hz, 1H, H-8), 3.38 (s, 3H, H- 32), 3.07 – 3.02 (d, *J* = 14.2 Hz, 1H, H-15), 1.92 – 1.60 (m, 10H, CH_2_-*CH*_2_-CH_3_), 1.09 (dt, *J* = 8.5, 7.4 Hz, 6H, CH_2_-*C*H_2_-C*H*_3_), 0.79 (dt, *J* = 19.4, 7.5 Hz, 6H, CH_2_-*C*H_2_-C*H*_3_) ppm. **^13^C NMR (151 MHz, CDCl_3_)** *δ =* 152.7 (C-7), 152.5 (C-21), 151.3 (C-11), 151.23 (C-32), 151.19 (C-28), 150.7 (C-14), 150.6 (C-18), 150.0 (C-4), 137.8 (C-2), 136.1 (C-20), 135.5 (C-16), 134.4 (s,C-23), 133.2 (C-9), 133.0 (C-13), 132.6 (C-6), 132.1 (C-27), 117.8 (C-5), 116.8 (C-19), 112.3 (C-3), 112.2 (C-19), 110.8 (C-10), 110.4 (C-24), 109.4 (C-12), 108.4 (C-26), 77.6 (*C*H_2_-CH_2_-CH_3_), 77.1 (*C*H_2_-CH_2_-CH_3_), 77.0 (*C*H_2_-CH_2_-CH_3_), 76.8 (*C*H_2_-CH_2_-CH_3_), 57.2 (C-31), 57.0 (C-29), 55.8 (C-30, C-32), 33.3 (C-8), 32.5 (C-22), 32.0 (C-15), 30.3 (C-1), 23.6 (CH_2_-*C*H_2_-CH_3_), 23.6 (CH_2_-*C*H_2_-CH_3_), 22.2 (CH_2_-*C*H_2_-CH_3_), 21.9 (CH_2_-*C*H_2_-CH_3_), 11.0 (CH_2_-CH_2_-*C*H_3_), , 10.9 (CH_2_-CH_2_-*C*H_3_), 9.8 (CH_2_-CH_2_-*C*H_3_), 9.6 (CH_2_-CH_2_-*C*H_3_) ppm. Only the NMR-Signals from the main product at *T =* 25°C are listed. **IR (KBr):** *𝜐̃* = 3410 (s), 2999 (vs), 2961 (s), 2932 (s), 2874 (s), 1734 (s), 1663 (s), 1582 (s), 1454 (m), 1433 (m), 1402 (s), 1381 (s), 1319 (s), 1294 (s), 1227 (m), 1188 (s), 1080 (m), 1014 (vw), 953 (w), 932 (m), 835 (s) cm^-1^. **UV/VIS** (CH_2_Cl_2_): *λ* = 297.6. **HRMS (MALDI-TOF+, DCTB):** *m/z* calcd. for [M+K]^+^ C_44_H_52_Br_4_O_8_K^+^ : 1063.0022, found: 1063.0038. **Elemental Analysis**: Calcd. for C_44_H_52_O_8_Br_4_∙6H_2_O (%): C 46.50, H 5.68, found: C 46.38, H 5.89.

3^rd^ fraction (*R*_f_ = 0.06): 566 mg (22%) of 5,11,17,23-tetramethoxy-6,10,16,24-tetrabromo-25,26,27,28-tetrapropoxycalix[4]arene **8** as yellow solid.

**M.p.:** 85°C. **^1^H NMR (600 MHz, CDCl_3_)** *δ* = 6.90 (s, 1H, H-3), 6.80 (s, 1H, H-19), 5.94 (s, 1H, H-12), 5.86 (s, 1H, H-24), 4.52 (d, *J* = 14.2 Hz, 1H, H-1), 4.47 (d, *J* = 13.1 Hz, 1H, H-22), 4.40 (d, *J* = 13.5 Hz, 1H, H-15), 4.35 (d, *J* = 14.9 Hz, 1H, H-8), 4.05 – 3.98 (m, 4H, H. H-8, H/33/36/39/42), 3.97 (s, 3H, OC*H*_3_, Ar-OC*H*_3,_ H-31), 3.97 – 3.92 (m, 2H,H-15), 3.90 (s, 3H, OC*H*_3_, Ar-OC*H*_3_, H-29), 3.61 (t, *J* = 6.8 Hz, 2H, O-C*H*_2_), 3.58 (t, *J* = 6.8 Hz, 2H, O-C*H*_2_), 3.36 (s, 3H, OC*H*_3_, H-32), 3.34 (s, 3H, OC*H*_3_, H-30), 3.31 (d, *J* = 13.4 Hz, 1H, H-1), 3.10 (d, *J* = 13.3 Hz, 1H, H-22), 1.94 – 1.88 (m, 2H, CH_2_-C*H*_2_-CH_3_), 1.85 (pd, *J* = 7.2, 3.0 Hz, 4H, CH_2_-C*H*_2_-CH_3_), 1.74 – 1.60 (m, 2H, CH_2_-C*H*_2_-CH_3_), 1.08 (td, *J* = 7.4, 3.6 Hz, 6H, CH_2_-CH_2_-C*H*_3_), 0.89 (t, *J* = 7.4 Hz, 3H, CH_2_-CH_2_-C*H*_3_), 0.73 (t, *J* = 7.4 Hz, 3H, CH_2_-CH_2_-C*H*_3_) ppm. **^13^C NMR (151 MHz, CDCl_3_)** *δ* = 152.9 (C- 21), 152.0 (C-7), 151.84 (C-11), 151.77 (C-25), 151.72 (C-18), 151.3 (C-14), 151.1 (C-28), 150.1 (C-4), 138.6 (H-16), 135.5 (H-20), 135.4 (H-6), 135.1 (H-9), 134.5 (H-27), 133.1 (H-2), 131.1 (H-23), 130.9 (H-13), 116.5 (C-3), 116.1 (C-5), 112.9 (C-26), 112.25 (C-17), 112.24 (C-10), 112.03 (C-19), 111.99 (C-24), 111.0 (C-12), 78.0 (*C*H_2_-CH_2_-CH_3_), 77.8 (*C*H_2_-CH_2_-CH_3_), 77.5 (*C*H_2_-CH_2_-CH_3_), 75.9 (*C*H_2_-CH_2_-CH_3_), 57.2 (C-31), 57.2 (C-32), 57.1 (C-29), 57.0 (C-30), 34.3 (C-1), 32.9 (C-8), 31.3 (C-22), 29.7 (C-15), 23.6 (CH_2_-*C*H_2_-CH_3_), 23.6 (CH_2_-*C*H_2_-CH_3_), 23.2 (CH_2_-*C*H_2_-CH_3_), 20.7 (CH_2_-*C*H_2_-CH_3_), 10.9 (CH_2_-CH_2_-*C*H_3_), 10.9 (CH_2_-CH_2_-*C*H_3_), 10.0 (CH_2_-CH_2_-*C*H_3_), 9.6 (CH_2_-CH_2_-*C*H_3_) ppm. **IR (KBr):** *𝜐̃* = 2961 (s), 2934 (s), 2876 (m), 1585 (m), 1454 (vs), 1433 (vs), 1400 (m), 1383 (s), 1323 (m), 1298 (m), 1259 (m), 1231 (s), 1190 (m), 1146 (w), 1084 (vs), 1038 (m), 1003 (s), 972 (m), 932 (m), 889 (w), 835 (m), 789 (w), 775 (w), 758 (w), 542 (w) cm^-1^. **UV/VIS** (CH_2_Cl_2_): *λ* = 296.0 nm. **HRMS (MALDI-TOF+, DCTB):** *m/z* calcd. for [M]^+^ C_44_H_52_Br_4_O_8_^+^: 1024.0396, found: 1024.0385, *m/z* calcd. for [M+Na]^+^ C_44_H_52_Br_4_O_8_Na^+^ : 1047.0288, found: 1047.0286, *m/z* calcd. for [M+K]^+^ C_44_H_52_Br_4_O_8_K^+^ : 1063.0022, found: 1063.0038. **Elemental Analysis**: Calcd. for C_44_H_52_O_8_Br_4_ ·0.5 n-pentane (%): C 52.46, 5.49, found: C 52.55, H 5.67.

2.3 5,11,17,23-Tetramethoxy-4,10,16,22-tetravinylethoxy-25,26,27,28-tetrapropoxycalix[4]arene **11**

Calixarene **6** (450 mg, 438 µmol) was suspended in tetrahydrofuran (22 mL) and 1M K_2_CO_3(aq)_ (22 mL). After degassing of the solvents (5 min) with argon, trans-2-ethoxyvinylboronic acid pinacol ester (1.87 mL, 0.935 g/mL, 8.75 mmol) was added, followed by P(*^t^*Bu_3_)HBF_4_ (193 mg, 700 µmol) and Pd_2_dba_3_ (160.3 mg, 175 µmol). The mixture was stirred for 1 day at 80°C while vigorous stirring. The reaction mixture was cooled to room temperature, the layers were separated and the organic layer was washed with water (3 × 40 mL) and brine (40 mL) and dried over MgSO_4_. Further purification was performed by column chromatography (SiO_2_, ethyl acetate/petroleum ether 1:10) to give **11** in nearly quantitative yield with slight impurities.

Due to instability in solution and in air, the product was used without further purification for the next reaction. A pure sample (15.3 µmol, 31%) for ^1^H NMR and MS analysis was obtained by HPLC (SiO_2_, gradient n-hexane/ethyl acetate 5:1 (v:v) to pure ethyl acetate). **^1^H NMR (600 MHz, CD_2_Cl_2_**) *δ*= 6.69 (d, *J* = 12.8 Hz, 4H, CH=C*H*-OEt), 6.09 (s, 4H, Ar-*H*), 5.57 (d, *J* = 12.8 Hz, 4H, C*H*=CH-OEt), 4.39 (d, *J* = 14.0 Hz, 4H, Ar-*CH_2_*-Ar), 3.94 – 3.86 (m, 4H, C*H*_2_-CH_2_-CH_3_), 3.81 (q, *J* = 7.1 Hz, 8H, O-C*H*_2_-CH_3_), 3.67 – 3.61 (m, 5H, C*H*_2_-CH_2_-CH_3_), 3.52 (s, 12H, OMe), 3.44 (d, *J* = 14.0 Hz, 5H, C*H*_2_-CH_2_-CH_3_), 1.94 – 1.78 (m, 10H, CH_2_-C*H*_2_-CH_3_), 1.28 (t, *J* = 7.0 Hz, 13H, O-CH_2_-C*H*_3_), 0.96 (t, *J* = 7.5 Hz, 12H, CH_2_-CH_2_-C*H*_3_). **^13^C NMR (151 MHz, CD_2_Cl_2_)** *δ* = 152.1 (Ar*C*-OMe), 151.7 (Ar*C*-OPr), 150.1 (Ar-*C*H=*C*H-), 134.1 (Ar*C*-CH=CH-), 132.4 (Ar*C*-CH_2_-*C*Ar), 122.0 (Ar*C*-CH_2_-*C*Ar), 111.9 (Ar*C*-H), 101.7 (Ar-*C*H=*C*H-), 77.1 (Ar-O-*C*H_2_-CH_2_-CH_3_), 65.7 (Ar-O-*C*H_2_-CH_3_), 55.6 (OMe), 28.7 (s, 8C, Ar-*C*H_2_-Ar), 23.4 (Ar-O-CH_2_-*C*H_2_-CH_3_), 15.2 (O-CH_2_-*C*H_3_), 10.6 (O-CH_2_-CH_2_-*C*H_3_) ppm. **HRMS (MALDI-TOF+, DCTB):** *m/z* calcd. for [M]^+^ C_60_H_80_O_12_^+^: 992.5650:, found: 992.5641.

2.4 Nanocone **3**

BiOTf_3_ (48 mg, 73.7 µmol) was dissolved in dry dichloromethane (230 mL) and vinyl ether **11** (244 mg, 246 µmol, crude) dissolved in dry dichloromethane (10 mL) was added while stirring under an argon atmosphere in a closed vial. The reaction was heated to 60°C for 16 hours. After cooling to room temperature, the solution was washed with water (2 × 100 mL) and brine (100 mL) and dried over MgSO_4_. After evaporation of the solvent, the crude material was purified by column chromatography (ethyl acetate/petroleum ether 1:5; *R*_f_ = 0.36, 0.13, 0) to give:

1^st^ fraction: 33% yield (68 mg, 84.1 µmol) of nanocone **3** as colourless solid.

**M.p.:** 280°C start (dec.) **^1^H NMR (600 MHz, CDCl_3_)** *δ* = 7.07 (s, 8H, Ar-C*H*=C*H*-Ar), 4.70 (d, *J* = 11.4 Hz, 4H, Ar-C*H*_2_-Ar), 3.83 (t, *J* = 7.0 Hz, 8H, O-C*H*_2_-CH_2_-CH_3_), 3.31 (s, 12H, OMe), 2.79 (d, *J* = 11.3 Hz, 4H, Ar-C*H*_2_-Ar), 1.97 (h, *J* = 7.3 Hz, 8H, O-CH_2_-C*H*_2_-CH_3_), 1.16 (t, *J* = 7.4 Hz, 12H, O-CH_2_-CH_2_-C*H*_3_) ppm. **^13^C NMR (151 MHz, CDCl_3_)** *δ*= 149.3 (Ar*C*-OMe), 147.0 (Ar*C*-O-C_3_H_7_), 135.4 (ArC), 127.1 (Ar-*C*H=*C*H-Ar), 126.5 (ArC), 77.7 (O-*C*H_2_-CH_2_-CH_3_), 61.0 (OMe), 25.40 (s, 4C, Ar-*C*H_2_-Ar), 23.5 (O-CH_2_-*C*H_2_-CH_3_), 10.8 (O-CH_2_-CH_2_-*C*H_3_) ppm. **IR (KBr):** *𝜐̃* = 3026 (w), 2957 (w), 2928 (w), 2874 (w), 1740 (w), 1553 (w), 1452 (m), 1404 (m), 1379 (m), 1369 (m), 1267 (s), 1190 (m), 1096 (w), 1045 (vs), 978 (m), 960 (m), 910 (m), 860 (m), 812 (m), 756 (m), 706 (m), 635 (m). **UV/VIS** (**CH_2_Cl_2_**): *λ* = 317.6 (shoulder), 271.2 (shoulder) nm. **HRMS (MALDI-TOF+, DCTB):** *m/z* calcd. for [M]^+^ C_52_H_56_O_8_^+^: 808.3975, found: 808.3973, *m/z* calcd. for [M+Na]^+^ C_52_H_56_O_8_Na^+^: 831.3867:, found: 831.3869. **Elemental Analysis**: Calcd. for C_52_H_56_O_8_·2/3CH_2_Cl_2_ (%): C 73.08, H 6.68, O 14.79, Cl 5.46, found: C 73.18, H 6.89.

2^nd^ fraction: 11% yield of **12** that needed to be further purified by recycling-HPLC (SiO_2_, 20 mL/min, n-heptane/ethyl acetate 4:1 (v:v)) to give 7.6 mg of compound **12.**

**^1^H NMR (600 MHz, CDCl_3_)** *δ =* 7.49 (s, 1H, OH), 7.15 – 6.99 (m, 6H, Ar-C*H*-C*H*-Ar), 6.38 (s, 1H, Ar-*H*, H-20), 6.27 (s, 1H, Ar-*H*, H-3),4.95 (d, *J* = 11.3 Hz, 1H, H-32), 4.79 (d, *J* = 10.9 Hz, 1H, H-28), 4.67 (d, *J* = 11.5 Hz, 1H, H-24), 4.27 (d, *J* = 12.8 Hz, 1H, Ar-CH_2_-Ar,H-1), 4.02 – 3.78 (m, 7H), 3.67 (s, 3H, OMe, H-38), 3.65 (s, 3H, OMe, H-35), 3.63 (s, 3H, OMe, H-36), 3.61 (s, 3H, OMe, H-37), 3.28 (d, *J* = 12.9 Hz, 1H, Ar-CH_2_-Ar, H-1), 2.78 (d, *J* = 11.5 Hz, 1H, H-24), 2.72 (d, *J* = 10.9 Hz, 1H, H-28), 2.61 (d, *J* = 11.3 Hz, 1H, H-32), 2.26 – 1.89 (m, 6H, -O-CH_2_-C*H*_2_-CH_3_), 1.30 (t, *J* = 7.5 Hz, 6H, -O-CH_2_-CH_2_-C*H*_3_), 1.08 (d, *J* = 7.4 Hz, 3H, -O-CH_2_-CH_2_-C*H*_3_) ppm.**^13^C NMR (151 MHz, CDCl_3_)** *δ* = 152.73 (C-19), 149.6 (C-4), 149.5 (C-9), 149.5 (C-14), 146.9 (ArC-OPr, C-30), 146.8 (ArC-OPr, C-26), 144.60 (ArC-OPr, C-22), 143.6 (ArC-OH), 135.7 (C-21), 135.6 (C-29/27), 135.3 (C-31), 135.1 (C-27/29), 134.6 (C-25), 133.06 (C-23), 130.5 (C-2), 130.0 (C-33), 126.9 (C-10/13), 126.8 (C-6/7/11/12/16/17), 126.7 (C-6/7/11/12/16/17), 126.6 (C-8), 126.2 (C-10/13), 126.2 (C-6/7/11/12/16/17), 126.20 (C-15), 126.1 (C-6/7/11/12/16/17), 126.0 (C-6/7/11/12/16/17), 125.6 (C-6/7/11/12/16/17), 124.16 (C-18), 123.8 (C-5), 108.8 (C-20), 108.1 (C-3), 78.7 (O-*C*H_2_-), 77.9 (O-*C*H_2_-), 77.5 (O-*C*H_2_-), 61.3 (C-36, C-37), 55.7 (C-35), 55.5 (C-38), 33.5 (C-1), 25.6 (C-24, C-28, C-32), 24.0 ( -O-CH_2_-*C*H_2_-CH_3_), 23.4 (-O-CH_2_-*C*H_2_-CH_3_), 23.3 (-O-CH_2_-*C*H_2_-CH_3_), 11.1 (-O-CH_2_-CH_2_-*C*H_3_), 10.8 (-O-CH_2_-CH_2_-*C*H_3_), 10.7 (-O-CH_2_-CH_2_-*C*H_3_) ppm. **IR (KBr):** *𝜐̃* =3653 (vs), 3333 (s), 2961 (m), 2932 (m), 2876 (s), 1595 (s), 1458 (m), 1448 (m), 1412 (m), 1379 (m), 1321 (s), 1261 (m), 1103 (m), 1051 (vw), 972 (s), 955 (s), 818 (vs), 795 (s) cm^-1^. **HRMS (MALDI-TOF+, DCTB):** *m/z* calcd. for [M]^+^ C_47_H_50_O_8_^+^: 742.3506 found: 742.3500.

2.5 Octahydronanocone **13**

Nanocone **3** (155mg, 192 mmol) was dissolved in ethyl acetate/methanol (15 mL:1 mL). Pd/C (5 wt%, 50 mg, 479 µmol) was added and the mixture was stirred under a hydrogen atmosphere (50 bar) for 2 days at 50°C. The cooled suspension was filtered through a plug of Celite^©^ and washed with ethyl acetate (100 mL). After evaporation of the solvent, the crude material was purified by column chromatography (ethyl acetate/petroleum ether 1/4, *R*_f_ = 0.15) to give **12** in 96% yield as a colorless solid (150 mg, 184 µmol). **M.p:** 236°C (dec.) **^1^H NMR (600 MHz, CDCl_3_)** *δ =* 4.51 (d, *J* = 12.8 Hz, 4H, Ar-C*H*_2_-Ar), 3.68 (t, *J* = 7.0 Hz, 8H, O-C*H*_2_-CH_2_-CH_3_), 3.52 (d, *J* = 12.8 Hz, 4H, Ar-C*H*_2_-Ar), 3.47 (s, 12H, OMe), 3.12 – 3.01 (m, 8H, Ar-CH_2_-C*H*_2_-Ar), 2.96 – 2.87 (m, 8H, Ar-C*H*_2_-C*H*_2_-Ar), 1.92 (q, *J* = 7.2 Hz, 8H, O-CH_2_-C*H*_2_-CH_3_), 1.12 (t, *J* = 7.4 Hz, 12H, O-CH_2_-CH_2_-C*H*_3_) ppm. **^13^C NMR (151 MHz, CDCl_3_)** *δ* = 152.1 (Ar*C*-OMe), 149.7 (Ar*C*-OPr), 133.1 (Ar*C*-CH_2_), 128.6 (Ar*C*-CH_2_), 77.4 (O-*C*H_2_-CH_2_-CH_3_), 61.0 (OMe), 23.8 (Ar-*C*H_2_-*C*H_2_-Ar), 23.6 (Ar-*C*H_2_-Ar)., 23.6 (O-CH_2_-*C*H_2_-CH_3_), 11.0 (O-CH_2_-CH_2_-*C*H_3_) ppm. **IR (KBr):** *𝜐̃* =2961 (s), 2934 (s), 2876 (m), 2361 (w), 1574 (vw), 1454 (s), 1425 (m), 1414 (m), 1387 (m), 1323 (m), 1256 (vs), 1196 (m), 1111 (m), 1096 (s), 1053 (vs), 991 (m), 964 (w), 953 (m), 467 (w). **UV/VIS** (CH_2_Cl_2_): *λ* = 282.0 nm. **HRMS (MALDI-TOF+, DCTB):** *m/z* calcd. for [M]^+^ C_52_H_64_O_8_^+^: 816.4601, found: 816.4597, *m/z* calcd. for [M+Na]^+^ C_52_H_64_O_8_Na^+^: 839.4493:, found: 839.4494. **Elemental Analysis**: Calcd. for C_52_H_56_O_8_·1.5 CH_2_Cl_2_ (%): C 73.39, H 8.07, O 18.54, found: C 73.47, H 8.49.

2.6 Sodium triflate complex (Na^+^⸦**3**)(OTf^-^)

Nanocone **3** (7.7 mg, 9.52 µmol) and NaOTf (11.8 mg, 75.6 µmol) were suspended in 1 mL CHCl_3_ and stirred at room temperature for 30 min. The suspension was filtered through cotton and washed with 1 mL CHCl_3_. The solution was evaporated in vacuo to give (Na^+^⸦**3**)(OTf^-^) in quantitative yield (9.5 mg, 9.68 µmol) as colorless solid. **M.p.:** 250-252 °C (dec). **^1^H NMR (600 MHz, CDCl_3_)** *δ =* 7.11 (s, 8H, Ar-C*H*=C*H*-Ar), 4.61 (d, *J* = 11.7 Hz, 4H, Ar-C*H*_2_-Ar), 4.27 – 4.19 (m, 8H, O-C*H*_2_-CH_2_-CH_3_), 3.41 (s, 12H, O-Me), 2.88 (d, *J* = 11.8 Hz, 4H, Ar-C*H*_2_-Ar), 2.10 – 1.98 (m, 8H, O-CH_2_-C*H*_2_-CH_3_), 1.01 (t, *J* = 7.4 Hz, 12H, O-CH_2_-CH_2_-C*H*_3_). **^19^F NMR (471 MHz, CDCl_3_)** *δ* = -78.41 ppm. **^13^C NMR** **(151 MHz, CDCl_3_)** *δ =* 150.8 (Ar*C*-OMe), 143.9 (Ar*C*-OPr), 134.8 (Ar*C*), 127.2 (Ar-*C*H_2_=*C*H_2_-Ar), 126.9 (Ar*C*), 80.04 (Ar-O-*C*H_2_-), 61.1 (OMe), 26.1 (Ar-*C*H_2_-Ar), 22.7 (O-CH_2_-*C*H_2_-CH_3_), 9.6 (O-CH_2_-CH_2_-*C*H_3_) ppm.

2.7 Lithium triflate complex (Li^+^⸦**3**)(OTf^-^)

Truncated cone **3** (5.4 mg, 6.67 µmol) and LiOTf (13.0 mg, 83.3 µmol) were suspended in 1 mL CHCl_3_ and stirred at room temperature for 3d. The suspension was filtered through cotton and washed with 1 mL CHCl_3_. The solution was evaporated in vacuo to give (Li^+^⸦**3**)(OTf^-^) in quantitative yield (6.5 mg, 6.62 µmol). About 5% of NaOTf is in LiOTf, due to higher binding constants. **^1^H NMR (600 MHz, CDCl_3_)** *δ =* 7.12 (s, 9H, Ar-C*H*=C*H*-Ar), 4.68 (d, *J* = 11.5 Hz, 4H, Ar-C*H*_2_-Ar), 4.51 – 4.40 (m, 8H, O-C*H*_2_-CH_2_-CH_3_), 3.43 (s, 12H, OMe), 2.80 (d, *J* = 11.4 Hz, 4H, Ar-C*H*_2_-Ar), 2.09 – 1.98 (m, 8H, O-CH_2_-C*H*_2_-CH_3_), 0.96 (t, *J* = 7.3 Hz, 12H, O-CH_2_-CH_2_-C*H*_3_) ppm. **^13^C NMR (151 MHz, CDCl_3_)** *δ =* 150.7 (Ar*C*-OMe), 143.9 (Ar*C*-OPr), 134.8 (Ar*C*), 127.0 (Ar-*C*H_2_=*C*H_2_-Ar), 126.6 (Ar*C*), 79.8 (Ar-O-*C*H_2_-), 61.2 (OMe), 26.8 (Ar-*C*H_2_-Ar), 22.3 (O-CH_2_-*C*H_2_-CH_3_), 9.4 (O-CH_2_-CH_2_-*C*H_3_) ppm.

2.8 Potassium triflate complex (K^+^⸦**3**)(OTf^-^)

Nanocone **3** (11.3 mg, 14.0 µmol) and KOTf (14.0 mg, 114 µmol) were suspended in 1 mL CHCl_3_ and stirred at room temperature for 30 min. The suspension was filtered through cotton and washed with 1 mL CHCl_3_. The solution was evaporated in vacuo to give (K^+^⸦**3**)(OTf^-^)

in quantitative yield (13.5 mg, 14.4 µmol). **^1^H NMR (600 MHz, CDCl_3_)** *δ* = 7.10 (s, 8H, (s, 8H, Ar-C*H*=C*H*-Ar)), 4.53 (d, *J* = 11.9 Hz, 4H, (d, *J* = 11.7 Hz, 4H, Ar-C*H*_2_-Ar), 4.08 – 3.96 (m, 8H, O-C*H*_2_-CH_2_-CH_3_), 3.39 (s, 12H, O-Me), 2.93 (d, *J* = 11.8 Hz, 4H, Ar-C*H*_2_-Ar), 2.02 – 1.89 (m, 8H, O-CH_2_-CH_2_-C*H*_3_), 1.04 (t, *J* = 7.4 Hz, 12H, O-CH_2_-CH_2_-C*H*_3_) ppm. **^13^C NMR** **(151 MHz, CDCl_3_)** *δ =* 150.6 (Ar*C*-OMe), 144.2 (Ar*C*-OPr), 134.8 (Ar*C*), 127.2 (Ar-*C*H_2_=*C*H_2_-Ar), 127.0 (Ar*C*), 79.7 (Ar-O-*C*H_2_), 61.1 (OMe), 25.5 (Ar-*C*H_2_-Ar), 22.8 (O-CH_2_-*C*H_2_-CH_3_), 10.0 (O-CH_2_-CH_2_-*C*H_3_) ppm.

1. **NMR Spectra**

**Figure S1:** ^1^H NMR spectrum of calixarene **5** (CDCl_3_, 600 MHz).

**Figure S2:** ^13^C NMR spectrum of calixarene **5** (CDCl_3_, 151 MHz).

**Figure S3:** ^1^H,^1^H COSY NMR spectrum of calixarene **5** (CDCl_3_, 600/600 MHz).

**Figure S4:** ^1^H,^13^C HSQC NMR spectrum of calixarene **5** (CDCl_3_, 600/151 MHz).

**Figure S5**: ^1^H,^13^C HMBC NMR spectrum of calixarene **5** (CDCl_3_, 600/151 MHz).

**Figure S6:** ^1^H NMR spectrum of bromocalixarene **6** (CDCl_3_, 700 MHz, 323 K).

**Figure S7:** ^1^H NMR spectrum of bromocalixarene **6** (CDCl_3_, 300 MHz, 223 K). #: CH_2_Cl_2_.

**Figure S8:** ^13^C NMR spectrum of bromocalixarene **6** (CDCl_3_, 171 MHz, 323 K).

**Figure S9:** ^1^H,^1^H COSY NMR spectrum of bromocalixarene **6** (CDCl_3_, 700/700 MHz, 323 K).

**Figure S10:** ^1^H,^13^C HMBC NMR spectrum of bromocalixarene **6** (CDCl_3_, 700/171 MHz, 323 K).

**Figure S11:** ^1^H,^13^C HMBC NMR spectrum of bromocalixarene **6** (CDCl_3_, 600/171 MHz, 323 K).

**Figure S12:** ^1^H NMR spectrum of bromocalixarene **7** (CDCl_3_, 600 MHz). */+: H_2_O/PE

**Figure S13:** ^13^C NMR spectrum of bromocalixarene **7** (CDCl_3_, 151 MHz).

**Figure S14:** ^1^H,^1^H COSY NMR spectrum of bromocalixarene **7** (CDCl_3_, 600/600 MHz).

**Figure S15**: ^1^H,^1^H NOESY NMR spectrum of bromocalixarene **7** (CDCl_3_, 600/600 MHz).

**Figure S16:** zoom in section of ^1^H,^1^H NOESY NMR spectrum of bromocalixarene **7** (CDCl_3_, 600/600 MHz).

**Figure S17**: ^1^H,^13^C HSQC NMR spectrum of bromocalixarene **7** (CDCl_3_, 600/151 MHz).

**Figure S18:** ^1^H,^13^C HMBC NMR spectrum of bromocalixarene **7** (CDCl_3_, 600/151 MHz).

**Figure S19:** Band selective ^1^H,^13^C HMBC NMR spectrum of bromocalixarene **7** (CDCl_3_, 700/176 MHz).

**Figure S20:** Band selective ^1^H,^13^C HMBC NMR spectrum of bromocalixarene **7** (CDCl_3_, 700/176 MHz).

**Figure S21:** Band selective ^1^H,^13^C HMBC NMR spectrum of bromocalixarene **7** (CDCl_3_, 700/176 MHz).

**Figure S22:** ^1^H NMR spectrum of bromocalixarene **8** (CDCl_3_, 600 MHz). *: CH_2_Cl_2_., +: H_2_O

**Figure S23:** Zoom in section of ^1^H NMR spectrum of bromocalixarene **8** (CDCl_3_, 600 MHz).

**Figure S24:** ^13^C NMR spectrum of bromocalixarene **8** (CDCl_3_, 151 MHz).

**Figure S25:** Zoom in of ^13^C NMR spectrum of bromocalixarene **8** (CDCl_3_, 151 MHz).

**Figure S26:** ^1^H, ^1^H COSY NMR spectrum of bromocalixarene **8** (CDCl_3_, 600/600 MHz).

**Figure S27:** Zoom-in section of ^1^H, ^1^H COSY NMR spectrum of bromocalixarene **8** (CDCl_3_, 600/600 MHz).

**Figure S28:** ^1^H,^13^C HSQC NMR spectrum of bromocalixarene **8** (CDCl_3_, 600/151 MHz).

**Figure S29:** ^1^H,^13^C HMBC NMR spectrum of bromocalixarene **8** (CDCl_3_, 600/151 MHz).

**Figure S30:** Band selective ^1^H,^13^C HMBC NMR spectrum of bromocalixarene **8** (CDCl_3_, 700/171 MHz).

**Figure S31**: Bandselective ^1^H,^13^C HMBC NMR spectrum of bromocalixarene **8** (CDCl_3_, 700/176 MHz).

**Figure S32:** ^1^H NMR spectrum of bromocalixarene **7** (CDCl_3_, 600 MHz), */+: H_2_O/PE

**Figure S33:** ^1^H NMR spectrum of vinylethoxy calixarene **11** (CDCl_3_, 300 MHz). +: CH_2_Cl_2_.

**Figure S34:** ^1^H NMR spectrum of vinylethoxy calixarene **11** (CD_2_Cl_2_, 600 MHz). +: compound instable

**Figure S35:** ^13^C NMR spectrum of vinylethoxy calixarene **11** (CD_2_Cl_2_, 151 MHz).

**Figure S36:** ^1^H,^1^H NMR spectrum of vinylethoxy calixarene **11** (CD_2_Cl_2_, 600/600 MHz).

**Figure S37:** ^1^H,^13^C HSQC NMR spectrum of vinylethoxy calixarene **11** (CD_2_Cl_2_, 600/151 MHz).

**Figure S38:** ^1^H,^13^C HMBC NMR spectrum of vinylethoxy calixarene **11** (CD_2_Cl_2_, 600/151 MHz).

**Figure S39:** ^1^H NMR spectrum of nanocone **3** (CDCl_3_, 600 MHz). +: CH_2_Cl_2_., * water.

**Figure S40:** ^13^C NMR spectrum of nanocone **3** (CDCl_3_, 151 MHz).

**Figure S41:** ^1^H,^1^H COSY NMR spectrum of nanocone **3** (CDCl_3_, 600/600 MHz).

**Figure S42:** ^1^H,^13^C HSQC NMR spectrum of nanocone **3** (CDCl_3_, 600/151 MHz).

**Figure S43:** ^1^H,^13^C HMBC NMR spectrum nanocone **3** (CDCl_3_, 600/151 MHz).

**Figure S44:** ^1^H NMR spectrum of side product **12** (CDCl_3_, 600/151 MHz). * water.

**Figure S45:** ^13^C NMR of side-product **12** (CDCl_3_, 151 MHz).

**Figure S46:** ^1^H,^1^H COSY NMR spectrum of side-product **12** (CDCl_3_, 600/600MHz).

**Figure S47:** ^1^H,^13^C HSQC NMR spectrum of side-product **12** (CDCl_3_, 600/151 MHz).

**Figure S48:** ^1^H,^13^C HMBC NMR spectrum of side-product **12** (CDCl_3_, 600/151 MHz).

**Figure S49:** Band selective ^1^H,^13^C HMBC NMR spectrum of side-product **12** (CDCl_3_, 600/151 MHz).

**Figure S50:** Band selective ^1^H,^13^C HMBC NMR spectrum of side-product **12** (CDCl_3_, 600/151 MHz).

**Figure S51:** ^1^H NMR spectrum of reduced nanocone **13** (CDCl_3_, 600 MHz).

**Figure S52:** ^13^C NMR spectrum of reduced nanocone **13** (CDCl_3_, 151 MHz).

**Figure S53:** ^1^H,^1^H NOESY NMR spectrum of reduced nanocone **13** (CDCl_3_, 600/600 MHz).

**Figure S54:** ^1^H,^13^C HSQC NMR spectrum of reduced nanocone **13** (CDCl_3_, 600/151 MHz).

**Figure S55:** ^1^H,^13^C HMBC NMR spectrum of reduced nanocone **13** (CDCl_3_, 600/151 MHz).

**Figure S56**: ^1^H NMR spectrum of lithium triflate complex **14** (CDCl_3_, 600 MHz). * ~5% sodium triflate complex Li^+^⸦**3.**

**Figure S57.** ^13^C NMR spectrum of lithium triflate complex Li^+^⸦**3** (CDCl_3_, 151 MHz).

**Figure S58**: ^1^H^13^C HSQC NMR spectrum of lithium triflate complex Li^+^⸦**3** (CDCl_3_, 600/151 MHz).

**Figure S59:** ^1^H^13^C HMBC NMR spectrum of lithium triflate complex Li^+^⸦**3** (CDCl_3_, 600/151 MHz).

**Figure S60:** ^1^H NMR spectrum of sodium triflate complex Na^+^⸦**3** (CDCl_3_, 600 MHz).

**Figure S61:** ^19^F NMR of sodium triflate complex Na^+^⸦**3** (CDCl_3_, 471 MHz)

**Figure S62:** ^13^C NMR spectrum of sodium triflate complex Na^+^⸦**3** (CDCl_3_, 151 MHz).

**Figure S63:** ^1^H,^1^H COSY NMR spectrum of sodium triflate complex Na^+^⸦**3** (CDCl_3_, 600/600 MHz).**Figure S64:** ^1^H,^13^C HMBC NMR spectrum of sodium triflate complex Na^+^⸦**3** (CDCl_3_, 600/151 MHz).

**Figure S65:** ^1^H,^13^C HMBC NMR spectrum of sodium triflate complex Na^+^⸦**3** (CDCl_3_, 600/151 MHz).

**Figure S66:** ^1^H,^19^F HOESY NMR spectrum of sodium triflate complex Na^+^⸦**3** (CDCl_3_, 500/470 MHz).

**Figure S67**: ^1^H NMR spectrum of potassium triflate complex K^+^⸦**3** (CDCl_3_, 600 MHz).

**Figure S68:** ^19^F NMR of potassium triflate complex K^+^⸦**3** (CDCl_3_, 471 MHz)

**Figure S69:** ^13^C NMR spectrum of potassium triflate complex K^+^⸦**3** (CDCl_3_, 151 MHz).

**Figure S70:** ^1^H,^13^C HMBC NMR spectrum of potassium triflate complex K^+^⸦**3** (CDCl_3_, 600/151 MHz).

**Figure S71:** ^1^H,^13^C HSQC NMR spectrum of potassium triflate complex K^+^⸦**3** (CDCl_3_, 600/151 MHz).

**Figure S72:** ^1^H,^13^C HMBC NMR spectrum of sodium triflate complex K^+^⸦**3** (CDCl_3_, 600/151 MHz).

1. **Mass Spectra**

**Figure S73:** HR-MALDI-TOF mass spectrum (pos., DCTB) of calixarene **5**.

**Figure S74:** HR-MALDI-TOF mass spectrum (pos., DCTB) of bromocalixarene **6**.

**Figure S75:** HR-MALDI-TOF mass spectrum (pos., DCTB) of bromocalixarene **7**.

**Figure S76:** HR-MALDI-TOF mass spectrum (pos., DCTB) of bromocalixarene **8**.

**Figure S77:** MALDI-TOF mass spectrum (pos., DCTB) of vinlyethoxy calixarene **11**.

**Figure S78:** HR-MALDI-TOF mass spectrum (pos., DCTB) of nanocone **3**.

**Figure S79:** HR-MALDI-TOF mass spectrum (pos., DCTB) of sideproduct **12**.

**Figure S80:** HR-MALDI-TOF mass spectrum (pos., DCTB) of reduced nanocone **13**.

1. **FTIR Spectrometry**

**Figure S81:** FT-IR spectrum (ATR, ZnSe) of calixarene **5**.

**Figure S82:** FT-IR spectrum (ATR, ZnSe) of bromocalixarene **6**.


**Figure S83:** FT-IR spectrum (ATR, ZnSe) of bromocalixarene **7**.

**Figure S84:** FT-IR spectrum (KBr) of bromocalixarene **8**.

**Figure S85:** FT-IR spectrum (KBr) of nanocone **3**.

**Figure S86:** FT-IR spectrum (KBr) of side product **12**.

**Figure S87:** FT-IR spectrum (KBr) of reduced nanocone **13**.

1. **UV/vis Spectra**

**Figure S88:** UV/vis absorption spectrum of **8** measured in CH_2_Cl_2_.

**Figure S89:** UV/vis absorption spectrum of **7** measured in CH_2_Cl_2_.

**Figure S90:** UV/vis absorption spectrum of **3** measured in CH_2_Cl_2_.

**Figure S91:** UV/vis absorption spectrum of **13** measured in CH_2_Cl_2_.

1. **Crystallographic data**

7.1 Calixarene **5**

Crystals suitable for X-ray diffraction were obtained by evaporation of an ethanol/ CH_2_Cl_2_ mixture.


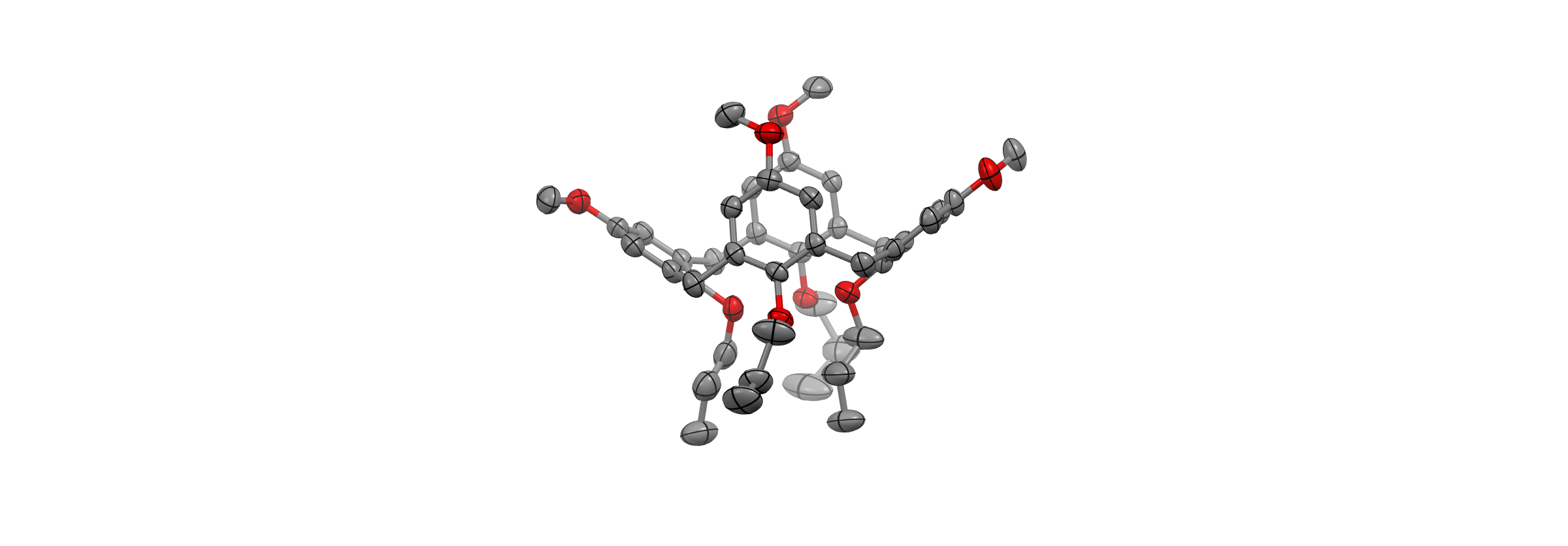


CCDC number 2434116

Empirical formula C_44_H_56_O_8_

Formula weight 712.88

Temperature 200(2) K

Wavelength 0.71073 Å

Crystal system monoclinic

Space group *P*21/n

Z 4

Unit cell dimensions a = 13.3758(4) Å α = 90 deg.

b = 20.4727(5) Å β = 101.5535(19) deg.

c = 14.9391(4) Å γ = 90 deg.

Volume 4008.01(19) Å^3^

Density (calculated) 1.18 g/cm^3^

Absorption coefficient 0.08 mm^-1^

Crystal shape hexagonal

Crystal size 0.131 x 0.106 x 0.066 mm^3^

Crystal colour colourless

Theta range for data collection 1.7 to 22.3 deg.

Index ranges -14≤h≤14, -21≤k≤21, -15≤l≤15

Reflections collected 30121

Independent reflections 5128 (R(int) = 0.0670)

Observed reflections 3086 (I > 2σ (I))

Absorption correction Semi-empirical from equivalents

Max. and min. transmission 0.96 and 0.92

Refinement method Full-matrix least-squares on F2

Data/restraints/parameters 5128 / 753 / 536

Goodness-of-fit on F2 1.02

Final R indices (I>2sigma(I)) R1 = 0.066, wR2 = 0.154

Largest diff. peak and hole 0.53 and -0.25 eÅ^-3^

7.2 Bromocalixarene **6**

Crystals suitable for X-ray diffraction were obtained by crashing out in acetonitrile.


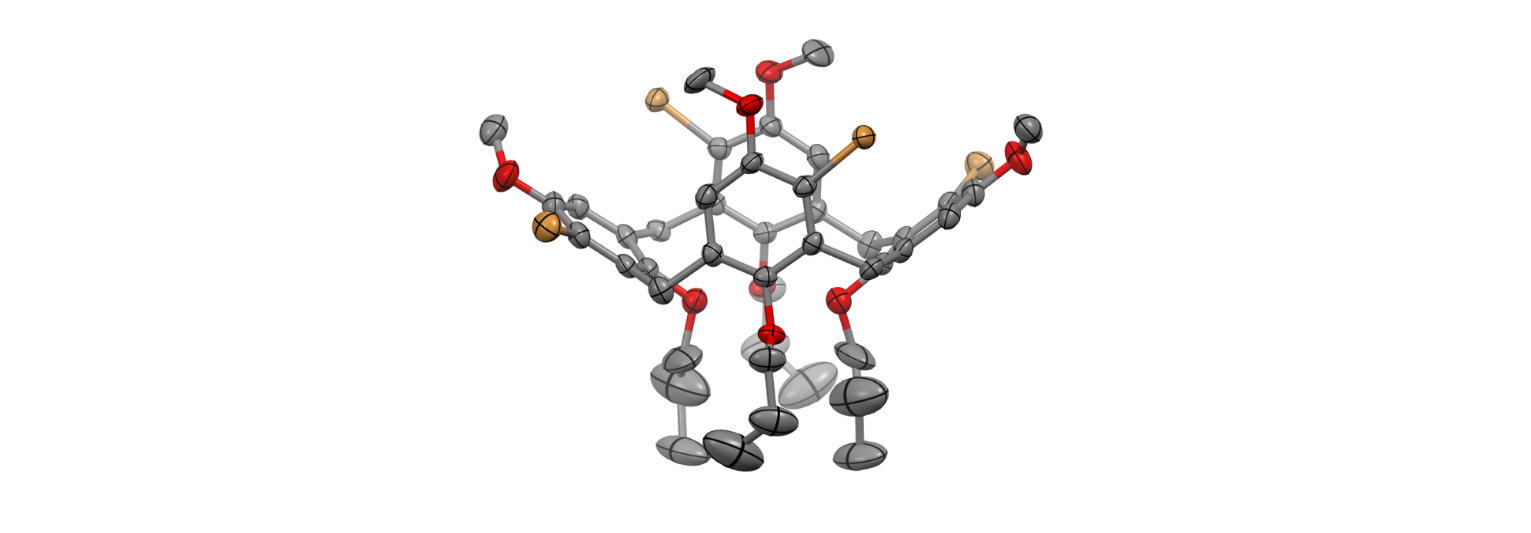


CCDC number 2434117

Empirical formula C44H52Br4O8.54

Formula weight 1037.15

Temperature 200(2) K

Wavelength 1.54178 Å

Crystal system tetragonal

Space group I$\bar{4}$ 2d

Z 8

Unit cell dimensions a = 19.7421(4) Å α = 90 deg.

b = 19.7421(4) Å β = 90 deg.

c = 23.5361(8) Å γ = 90 deg.

Volume 9173.2(5) Å^3^

Density (calculated) 1.50 g/cm3

Absorption coefficient 4.69 mm-1

Crystal shape prism

Crystal size 0.061 x 0.050 x 0.042 mm3

Crystal colour colourless

Theta range for data collection 2.9 to 71.7 deg.

Index ranges -24≤h≤15, -17≤k≤24, -22≤l≤28

Reflections collected 15802

Independent reflections 4328 (R(int) = 0.0327)

Observed reflections 3646 (I > 2σ(I))

Absorption correction Semi-empirical from equivalents

Max. and min. transmission 0.80 and 0.61

Refinement method Full-matrix least-squares on F2

Data/restraints/parameters 4328 / 0 / 260

Goodness-of-fit on F2 1.03

Final R indices (I>2sigma(I)) R1 = 0.037, wR2 = 0.087

Absolute structure parameter -0.017(15)

Largest diff. peak and hole 0.54 and -0.41 eÅ^-3^

7.3 Bromocalixarene **8**

Crystals suitable for X-ray diffraction were obtained in dimethylsulfoxide-d_6_ in an NMR tube.


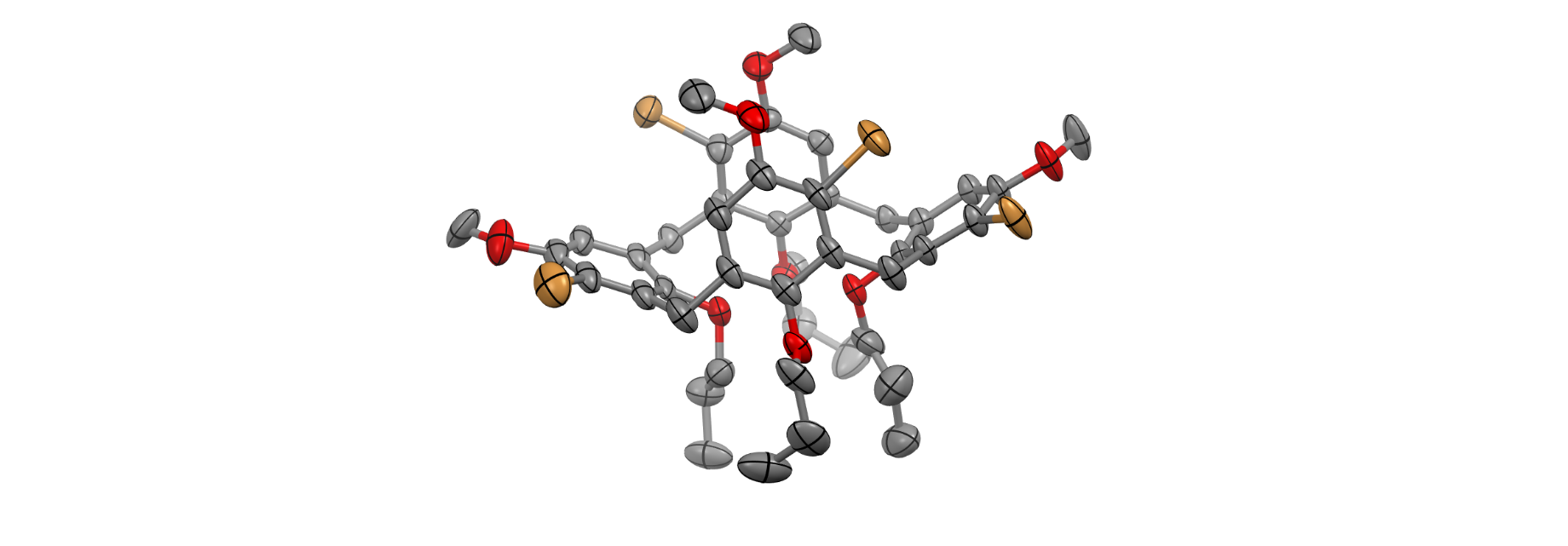


CCDC number 2434118

Empirical formula C44H52Br4O8

Formula weight 1028.49

Temperature 200(2) K

Wavelength 0.71073 Å

Crystal system triclinic

Space group P$\bar{1}$

Z 4

Unit cell dimensions a =12.4861(9) Å α = 86.6224(17) deg.

b = 16.5973(11) Å β = 83.7890(18) deg.

c =21.6636(14) Å γ = 84.4544(18) deg.

Volume 4436.9(5) Å3

Density (calculated) 1.54 g/cm3

Absorption coefficient 3.68 mm-1

Crystal shape prism

Crystal size 0.076 x 0.056 x 0.030 mm3

Crystal colour colourless

Theta range for data collection 0.9 to 23.5 deg.

Index ranges -14≤h≤14, -18≤k≤18, -24≤l≤24

Reflections collected 50575

Independent reflections 13168 (R(int) = 0.0767)

Observed reflections 7977 (I > 2σ(I))

Absorption correction Semi-empirical from equivalents

Max. and min. transmission 0.91 and 0.82

Refinement method Full-matrix least-squares on F2

Data/restraints/parameters 13168 / 2754 / 1083

Goodness-of-fit on F2 1.01

Final R indices (I>2sigma(I)) R1 = 0.058, wR2 = 0.105

Largest diff. peak and hole 1.08 and -1.04 eÅ^-3^

7.4 Bromocalixarene **7**

Crystals suitable for X-ray diffraction were obtained by evaporation of acetone and water.

^
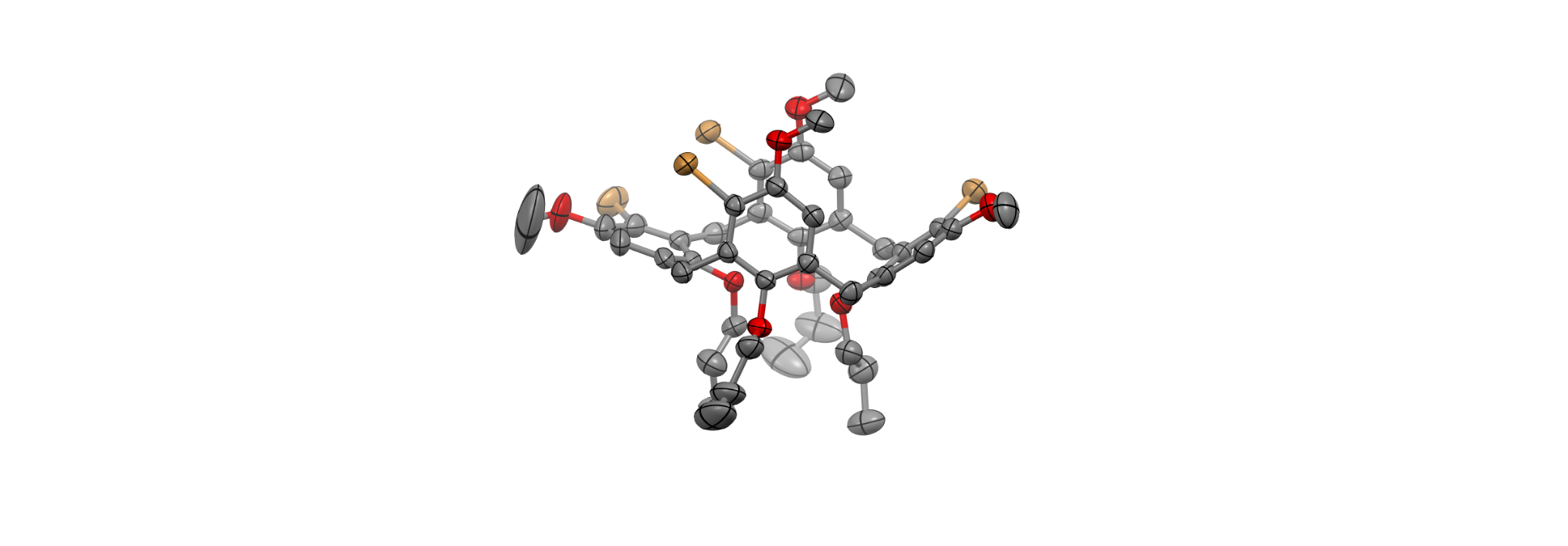
^

CCDC number 2434119

Empirical formula C_44_H_52_Br_4_O_8_

Formula weight 1028.49

Temperature 200(2) K

Wavelength 0.71073 Å

Crystal system triclinic

Space group P

Z 2

Unit cell dimensions a = 11.9949(10) Å α = 71.563(2) deg.

b = 13.7784(12) Å β = 84.612(2) deg.

c = 15.5995(14) Å γ = 85.657(2) deg.

Volume 2432.1(4) Å^3^

Density (calculated) 1.40 g/cm^3^

Absorption coefficient 3.36 mm^-1^

Crystal shape plate

Crystal size 0.088 x 0.060 x 0.032 mm^3^

Crystal colour colourless

Theta range for data collection 1.4 to 24.7 deg.

Index ranges -14≤h≤14, -16≤k≤16, -18≤l≤18

Reflections collected 30156

Independent reflections 8286 (R(int) = 0.0612)

Observed reflections 4945 (I > 2σ(I))

Absorption correction Semi-empirical from equivalents

Max. and min. transmission 0.91 and 0.85

Refinement method Full-matrix least-squares on F^2^

Data/restraints/parameters 8286 / 786 / 518

Goodness-of-fit on F^2^ 1.01

Final R indices (I>2sigma(I)) R1 = 0.049, wR2 = 0.107

Largest diff. peak and hole 0.63 and -0.50 eÅ^-3^

7.5 Vinylethoxy-methoxy-propoxy-calixarene **11**

Crystals suitable for x-ray diffraction were obtained after complete evaporation on a rotary evaporator of a column fraction (EA/PE) on a winter day in the Lab (T = <20°C). A crystal was isolated, set under argon in a closed vial and stored overnight in a freezer.


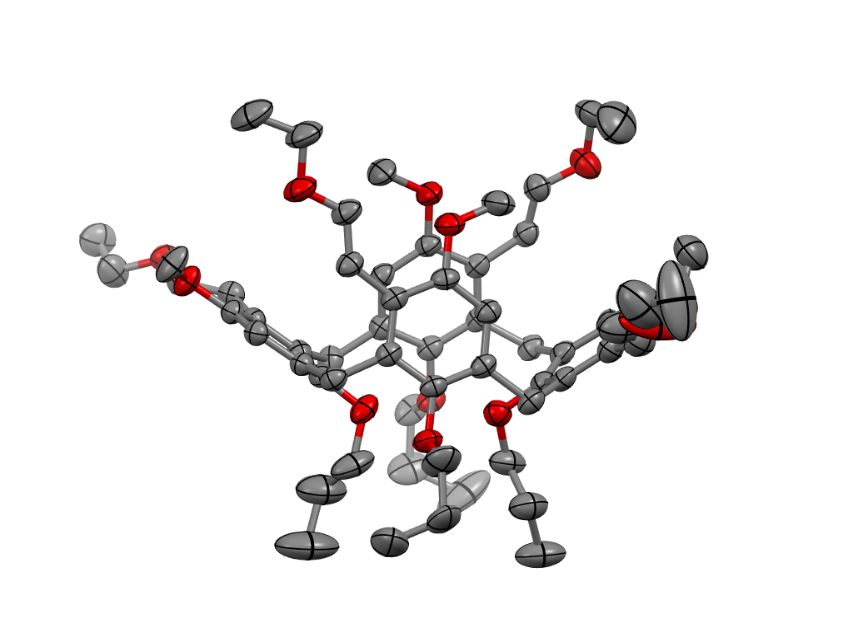


Identification code 2434123

Empirical formula C_60_H_80_O_12_

Formula weight 993.24

Temperature 200(2) K

Wavelength 0.71073 Å

Crystal system triclinic

Space group P$\bar{1}$

Z 2

Unit cell dimensions a = 11.8321(7) Å α = 89.0577(11) deg.

b = 11.8959(8) Å β = 85.2611(11) deg.

c = 21.4686(13) Å γ = 68.7913(10) deg.

Volume 2807.2(3) Å^3^

Density (calculated) 1.17 g/cm^3^

Absorption coefficient 0.08 mm^-1^

Crystal shape brick

Crystal size 0.156 x 0.138 x 0.125 mm^3^

Crystal colour colourless

Theta range for data collection 1.0 to 28.3 deg.

Index ranges -15≤h≤15, -15≤k≤15, -28≤l≤28

Reflections collected 55807

Independent reflections 13873 (R(int) = 0.0471)

Observed reflections 8375 (I > 2σ(I))

Absorption correction Semi-empirical from equivalents

Max. and min. transmission 0.96 and 0.93

Refinement method Full-matrix least-squares on F^2^

Data/restraints/parameters 13873 / 1121 / 825

Goodness-of-fit on F^2^ 1.03

Final R indices (I>2sigma(I)) R1 = 0.064, wR2 = 0.173

Largest diff. peak and hole 0.50 and -0.31 eÅ^-3^

7.6 Nanocone **3**

Crystals suitable for x-ray diffraction were obtained by vapour phase diffusion of acetonitrile in **3** dissolved in CHCl_3_.


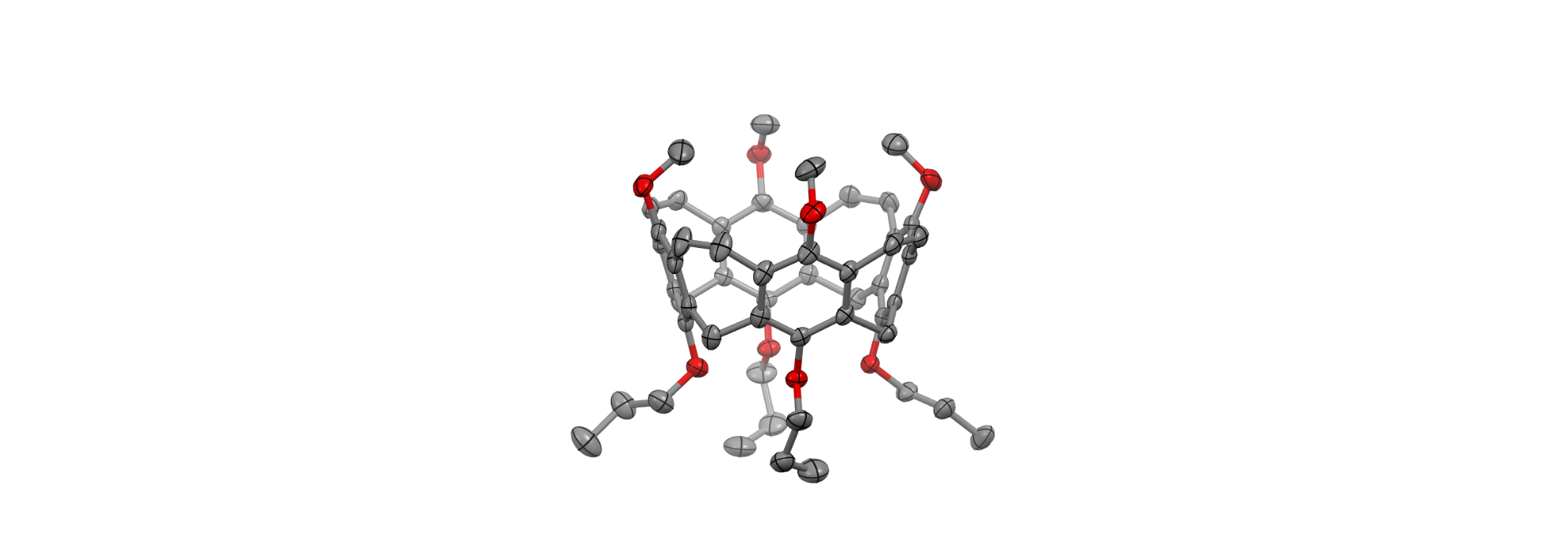


Identification code 2434119

Empirical formula C_52_H_56_O_8_

Formula weight 808.96

Temperature 200(2) K

Wavelength 0.71073 Å

Crystal system monoclinic

Space group C2/c

Z 8

Unit cell dimensions a = 35.120(2) Å α = 90 deg.

b = 14.7964(9) Å β = 108.2853(15) deg.

c = 17.5451(11) Å γ = 90 deg.

Volume 8657.0(9) Å^3^

Density (calculated) 1.24 g/cm^3^

Absorption coefficient 0.08 mm^-1^

Crystal shape brick

Crystal size 0.132 x 0.126 x 0.117 mm^3^

Crystal colour orange

Theta range for data collection 1.5 to 26.4 deg.

Index ranges -43≤h≤43, -18≤k≤18, -21≤l≤21

Reflections collected 44642

Independent reflections 8854 (R(int) = 0.0786)

Observed reflections 5422 (I > 2σ(I))

Absorption correction Semi-empirical from equivalents

Max. and min. transmission 0.96 and 0.92

Refinement method Full-matrix least-squares on F^2^

Data/restraints/parameters 8854 / 819 / 583

Goodness-of-fit on F^2^ 1.06

Final R indices (I>2sigma(I)) R1 = 0.070, wR2 = 0.143

Largest diff. peak and hole 0.34 and -0.25 eÅ^-3^

7.7 Compound **12**

Crystals suitable for X-ray diffraction were obtained after evaporation of CDCl_3_ in an NMR tube.


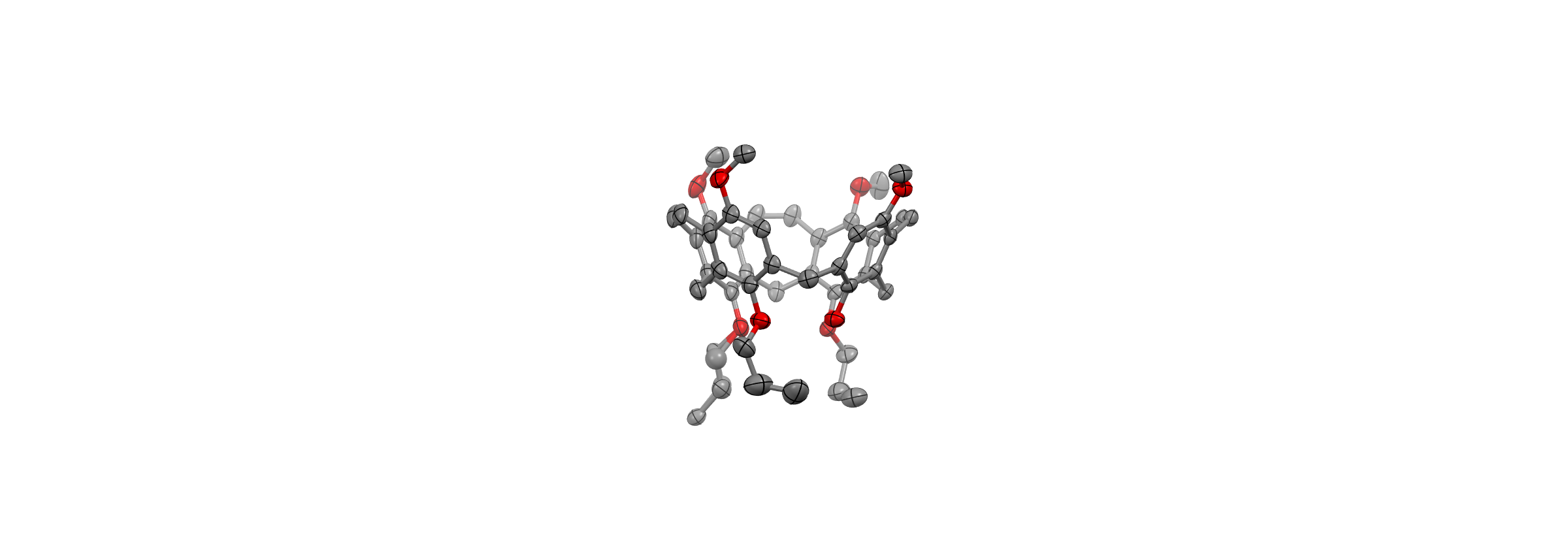


Identification code 2434121

Empirical formula C_47_H_50_O_8_

Formula weight 742.87

Temperature 200(2) K

Wavelength 0.71073 Å

Crystal system orthorhombic

Space group P2_1_2_1_2_1_

Z 4

Unit cell dimensions a = 11.3921(4) Å α = 90 deg.

b = 17.5153(7) Å β = 90 deg.

c = 19.4129(8) Å γ = 90 deg.

Volume 3873.6(3) Å^3^

Density (calculated) 1.27 g/cm^3^

Absorption coefficient 0.09 mm^-1^

Crystal shape cuboid

Crystal size 0.084 x 0.062 x 0.060 mm^3^

Crystal colour yellow

Theta range for data collection 2.1 to 25.7 deg.

Index ranges -13≤h≤13, -21≤k≤21, -23≤l≤23

Reflections collected 26025

Independent reflections 7354 (R(int) = 0.0557)

Observed reflections 5377 (I > 2σ(I))

Absorption correction Semi-empirical from equivalents

Max. and min. transmission 0.96 and 0.92

Refinement method Full-matrix least-squares on F^2^

Data/restraints/parameters 7354 / 30 / 514

Goodness-of-fit on F^2^ 1.04

Final R indices (I>2sigma(I)) R1 = 0.052, wR2 = 0.116

Absolute structure parameter 0.2(6)

Largest diff. peak and hole 0.30 and -0.17 eÅ^-3^

7.8 Reduced truncated cone **13**

Crystals suitable for x-ray diffraction were obtained by evaporation of pure **13** in CH_2_Cl_2_ on a rotary evaporator.


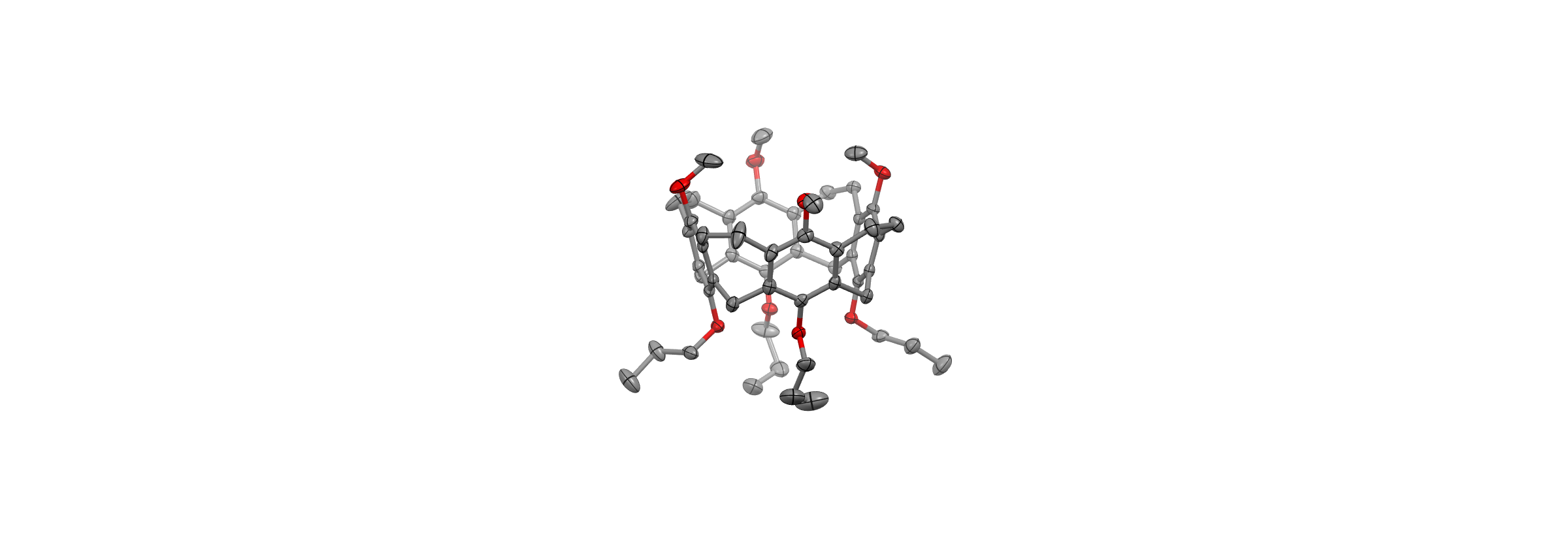


CCDC number 2434122

Empirical formula C_52_H_64_O_8.31_

Formula weight 821.99

Temperature 200(2) K

Wavelength 0.71073 Å

Crystal system triclinic

Space group P$\bar{1}$

Z 4

Unit cell dimensions a = 12.5887(7) Å α = 104.9703(13) deg.

b = 18.0882(10) Å β = 95.5267(13) deg.

c = 20.4308(11) Å γ = 99.0315(13) deg.

Volume 4392.7(4) Å^3^

Density (calculated) 1.24 g/cm^3^

Absorption coefficient 0.08 mm^-1^

Crystal shape polyherdron

Crystal size 0.115 x 0.091 x 0.060 mm^3^

Crystal colour colourless

Theta range for data collection 1.0 to 27.1 deg.

Index ranges -15≤h≤16, -22≤k≤22, -26≤l≤26

Reflections collected 79230

Independent reflections 19060 (R(int) = 0.0796)

Observed reflections 10600 (I > 2σ(I))

Absorption correction Semi-empirical from equivalents

Max. and min. transmission 0.96 and 0.92

Refinement method Full-matrix least-squares on F^2^

Data/restraints/parameters 19060 / 2228 / 1137

Goodness-of-fit on F^2^ 1.03

Final R indices (I>2sigma(I)) R1 = 0.074, wR2 = 0.182

Largest diff. peak and hole 0.84 and -0.34 eÅ^-3^

7.9 Nanocone with sodium triflate Na^+^⸦**3**

Crystals suitable for x-ray diffraction were obtained in CDCl_3_ in an NMR tube.


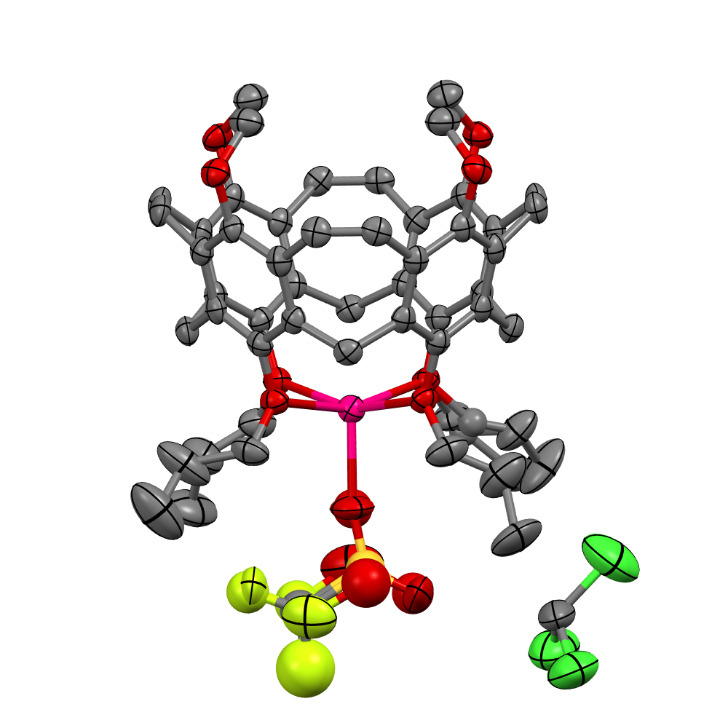


CCDC 2434124

Empirical formula C_54_H_57_Cl_3_F_3_NaO_11_S

Formula weight 1100.39

Temperature 200(2) K

Wavelength 1.54178 Å

Crystal system monoclinic

Space group P2_1_/c

Z 4

Unit cell dimensions a = 11.8480(4) Å α = 90 deg.

b = 16.6510(5) Å β = 96.216(2) deg.

c = 27.5640(8) Å γ = 90 deg.

Volume 5405.9(3) Å^3^

Density (calculated) 1.35 g/cm^3^

Absorption coefficient 2.56 mm^-1^

Crystal shape brick

Crystal size 0.115 x 0.083 x 0.043 mm^3^

Crystal colour pale yellow

Theta range for data collection 3.1 to 68.5 deg.

Index ranges -11≤h≤14, -16≤k≤20, -33≤l≤25

Reflections collected 50125

Independent reflections 9737 (R(int) = 0.0635)

Observed reflections 5350 (I > 2σ(I))

Absorption correction Semi-empirical from equivalents

Max. and min. transmission 0.87 and 0.78

Refinement method Full-matrix least-squares on F^2^

Data/restraints/parameters 9737 / 374 / 713

Goodness-of-fit on F^2^ 0.96

Final R indices (I>2sigma(I)) R1 = 0.057, wR2 = 0.142

Largest diff. peak and hole 0.50 and -0.38 eÅ^-3^

7.10 Nanocone with lithium triflate Li^+^⸦**3**

Crystals suitable for X-ray diffraction were obtained in CDCl_3_ in an NMR tube.


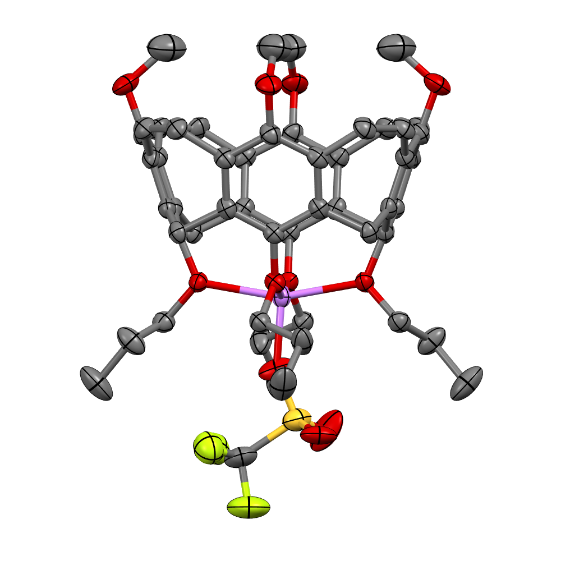


CCDC 2434125

Empirical formula C_53_H_56_F_3_LiO_12_S

Formula weight 980.97

Temperature 200(2) K

Wavelength 0.71073 Å

Crystal system tetragonal

Space group P4/n

Z 2

Unit cell dimensions a = 13.5742(7) Å α = 90 deg.

b = 13.5742(7) Å β = 90 deg.

c = 12.7272(10) Å γ = 90 deg.

Volume 2345.1(3) Å^3^

Density (calculated) 1.39 g/cm^3^

Absorption coefficient 0.15 mm^-1^

Crystal shape cuboid

Crystal size 0.094 x 0.052 x 0.047 mm^3^

Crystal colour yellow

Theta range for data collection 1.5 to 28.4 deg.

Index ranges -17≤h≤16, -17≤k≤16, -16≤l≤16

Reflections collected 17352

Independent reflections 2762 (R(int) = 0.0544)

Observed reflections 2100 (I > 2σ(I))

Absorption correction Semi-empirical from equivalents

Max. and min. transmission 0.96 and 0.89

Refinement method Full-matrix least-squares on F^2^

Data/restraints/parameters 2762 / 190 / 216

Goodness-of-fit on F^2^ 1.09

Final R indices (I>2sigma(I)) R1 = 0.075, wR2 = 0.215

Largest diff. peak and hole 1.06 and -0.53 eÅ^-3^

7.11 Nanocone with potassium triflate K^+^⸦**3**

Crystals suitable for X-ray diffraction were obtained in CDCl_3_ in a vial.


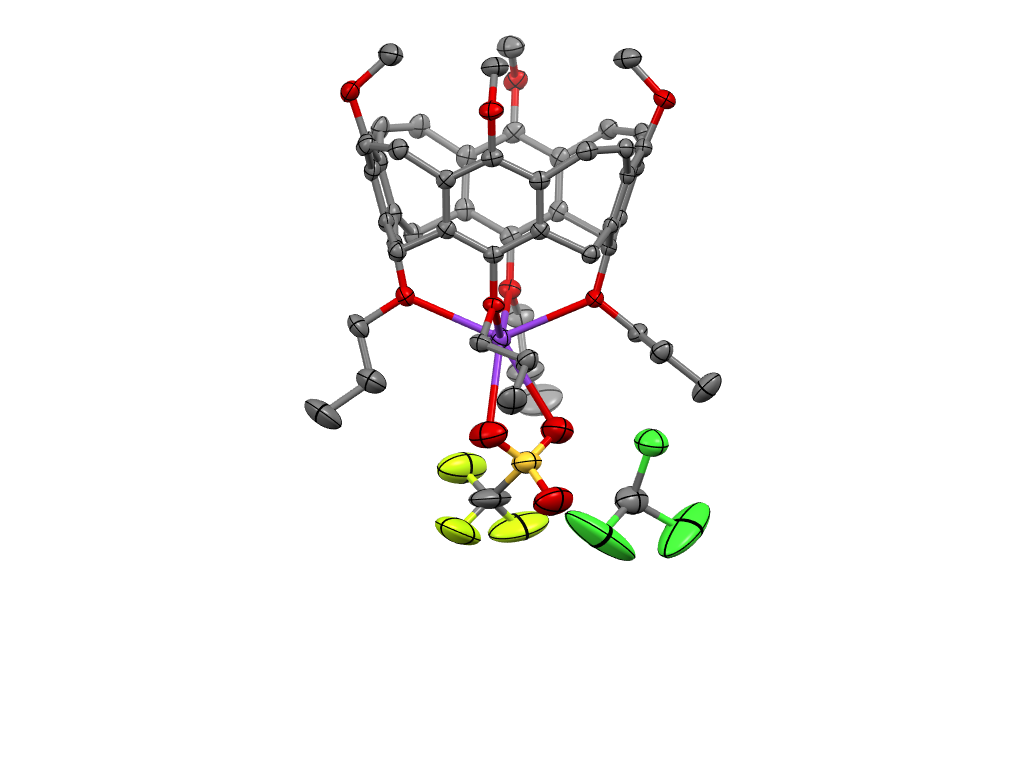


CCDC 2440262

Empirical formula C_54_H_57_Cl_3_F_3_KO_11_S

Formula weight 1116.50

Temperature 200(2) K

Wavelength 1.54178 Å

Crystal system orthorhombic

Space group Pnma

Z 4

Unit cell dimensions a = 27.7161(3) Å α = 90 deg.

b = 17.2798(2) Å β = 90 deg.

c = 11.4695(2) Å γ = 90 deg.

Volume 5493.07(13) Å^3^

Density (calculated) 1.35 g/cm^3^

Absorption coefficient 3.12 mm^-1^

Crystal shape plate

Crystal size 0.105 x 0.068 x 0.044 mm^3^

Crystal colour colourless

Theta range for data collection 4.1 to 68.6 deg.

Index ranges -33≤h≤15, -20≤k≤17, -13≤l≤11

Reflections collected 44075

Independent reflections 5199 (R(int) = 0.0527)

Observed reflections 3772 (I > 2σ(I))

Absorption correction Semi-empirical from equivalents

Max. and min. transmission 0.92 and 0.84

Refinement method Full-matrix least-squares on F^2^

Data/restraints/parameters 5199 / 0 / 347

Goodness-of-fit on F^2^ 1.05

Final R indices (I>2sigma(I)) R1 = 0.053, wR2 = 0.144

Largest diff. peak and hole 0.93 and -0.94 eÅ^-3^

7.12 Reduced truncated cone with lithium triflate Li^+^⸦**13**

Crystals suitable for X-ray diffraction were obtained in CDCl_3_ in an NMR tube.


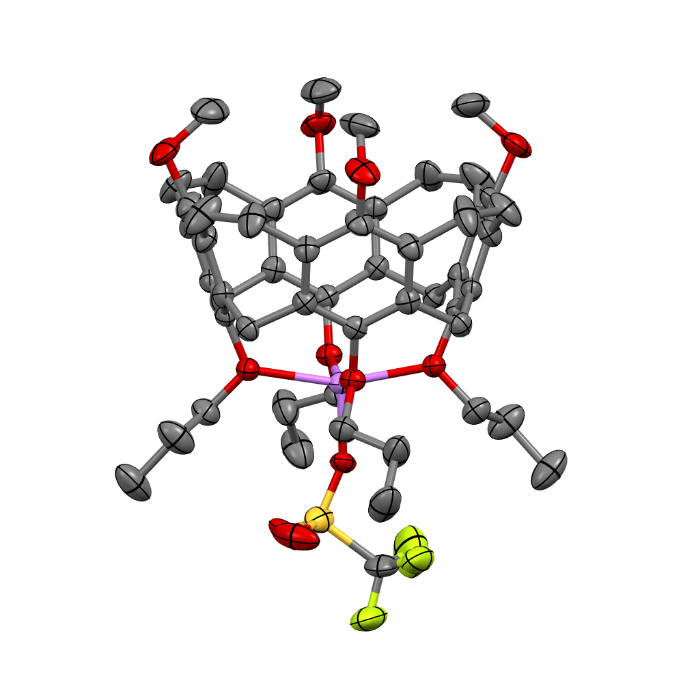


CCDC 2434126

Empirical formula C_53_H_64_F_3_LiO_11_S

Formula weight 973.04

Temperature 200(2) K

Wavelength 1.54178 Å

Crystal system tetragonal

Space group P4/n

Z 2

Unit cell dimensions a = 13.7438(4) Å α = 90 deg.

b = 13.7438(4) Å β = 90 deg.

c = 12.6286(5) Å γ = 90 deg.

Volume 2385.44(17) Å^3^

Density (calculated) 1.36 g/cm^3^

Absorption coefficient 1.23 mm^-1^

Crystal shape cuboid

Crystal size 0.103 x 0.050 x 0.028 mm^3^

Crystal colour colourless

Theta range for data collection 3.2 to 68.5 deg.

Index ranges -16≤h≤10, -16≤k≤14, -10≤l≤15

Reflections collected 11845

Independent reflections 2171 (R(int) = 0.0638)

Observed reflections 1456 (I > 2σ(I))

Absorption correction Semi-empirical from equivalents

Max. and min. transmission 0.95 and 0.84

Refinement method Full-matrix least-squares on F^2^

Data/restraints/parameters 2171 / 219 / 232

Goodness-of-fit on F^2^ 1.03

Final R indices (I>2sigma(I)) R1 = 0.055, wR2 = 0.132

Largest diff. peak and hole 0.44 and -0.20 eÅ^-3^

1. **Pinched cone interconversion of bromocalixarenes 6, 7 and 8**

The pinched cone to pinched cone interconversion (PCI) of the three bromine compounds **6-8** are analysed below. The PCI is temperature dependent and occurs on the NMR time scale either so quickly that only an averaged signal ‘TS’ is visible, or slow enough to show two signal sets, one for the parallel aromatic benzene rings (Scheme S1 number 2) and one for the aromatic rings pointing outwards (Scheme S1**Fehler! Verweisquelle konnte nicht gefunden werden.** number 1). Based on the measured NMR spectra at different temperature, $\Delta G^{\neq}$ was calculated using the Eyring equation:

$$\Delta G^{\neq}= RT\cdot(\ln\left( \frac{k_{B}}{h} \right)+\ln\left( \frac{T}{k} \right))+$$

*k* was determined according to a method from Baset and Anet ^[S^[^38^](#_ENREF_38)^]^and by Francis P. Gasparro and Nancy H. Kolodny ^[S^[^39^](#_ENREF_39)^]^

$k=\pi(w-w_{0})$ for T < T_c_

$k=\frac{2.2 {\Delta v}^{2}}{(w-w_{0})}$ for *T* > *T*_c_

$k=2.2 \Delta v$ for *T* = *T*_c_

with:

$k=$ reaction rate constant

$w=$ FWHM at requestes temperature

$w_{0}=$ FWHM at low temperature limit

$\Delta v$ = chemical shift difference at low temperature limit

T_c_ = coalescence temperature

**8.1** **Pinched cone interconversion of bromocalixarene 6**

**Scheme S1**: Representation of pinched cone interconversion of **6**.

Bromocalixarene **6** has two identical PC conformations due to its symmetry (see Scheme S1). At high temperatures above 263 K (see Figure S92), only one signal set is observed in the ^1^H NMR spectrum. During further cooling, the PCI becomes so slow compared to the NMR time scale that two signal sets appear (eg. Ar-H 1 and 2 in Figure S92 top), one for the parallel aromatic rings and one for the aromatic rings pointing outwards. The experimental value for the PCI of bromocalixarene **6** is $\Delta G^{\neq}$ = 11.7 kcal/mol in CDCl_3_.

**Figure S92:** ^1^H NMR of bromocalixarene **6** at different temperatures in CDCl_3_ (223 K to 293 K, 300 MHz).

**8.2** **Pinched cone interconversion of bromocalixarene 7**

**Scheme S2:** Representation of pinched cone interconversion of **7**.

Bromocalixarene **7** has also two identical PCs due to its symmetry. At room temperature, the PCI is slow and resolved signals are visible e.g. all for aromatic signals are a singlet. At a temperature at T = 413 K the PCI is fast enough compared to the NMR time scale to give only two broad signals. The measured value for the PCI of bromocalixarene **7** is $\Delta G^{\neq}$ = 18.1 kcal/mol in dimethylsulfoxide-d_6_.

**Figure S93:** top: visualisation of the PCI for **5**. Bottom: ^1^H NMR spectra of **7** in dimethylsulfoxide-d_6_ at different temperatures (298 K to 413 K).

**8.3** **Pinched cone interconversion of Bromocalixarenes8**

**Scheme S3:** Representation of pinched cone interconversion of **8**.

Bromocalixarene **8** is the only one that has two different pinched cone conformations (see Scheme S3). PC1 is according to the ^1^H NMR the dominant PC (that is the one found by single crystal X-ray diffraction), while the small signals are assigned to PC2. The integral ratio between PC1 and PC2 is changing with higher temperatures towards the disfavoured PC2 (see Table S1). A van’t Hoff plot is based on a linear temperature depending behaviour, while the fit of **8** is non-linear. According to literature,^[S40]^ a conformational change can change the enthalpy and entropy of a retention process, resulting in this unusual van’t Hoff plot.

**Figure S94**: ^1^H NMR spectra cut-out 500 MHZ in CD_2_Cl_2_ at different temperatures from **8.**

**Table S1:** Integrals of different pinched cone conformation (PC1 and PC2) of bromocalixarene **8** at different temperatures.

|  | Integrals PC1 | | | Integrals PC2 | | | | LN K |
| --- | --- | --- | --- | --- | --- | --- | --- | --- |
| T K | PC1_a_ | PC1_b_ | ØPC1 | PC2_a_ | PC2_b_ | PC2_C_ | ØPC2 | ln(PC1/PC2) |
| 283 | 1.00 | 1.00 | 1.00 | 0.22 | 0.21 | 0.20 | 0.21 | 1.57 |
| 273 | 1.00 | 1.00 | 1.00 | 0.20 | 0.19 | 0.18 | 0.19 | 1.66 |
| 263 | 1.00 | 0.99 | 0.99 | 0.19 | 0.17 | 0.17 | 0.18 | 1.73 |
| 253 | 1.00 | 0.99 | 0.99 | 0.18 | 0.16 | 0.16 | 0.17 | 1.79 |
| 243 | 1.00 | 0.99 | 1.00 | 0.17 | 0.16 | 0.15 | 0.16 | 1.84 |
| 233 | 1.00 | 1.07 | 1.00 | 0.16 | 0.16 | 0.14 | 0.16 | 1.89 |
| 223 | 1.00 | 1.00 | 1.00 | 0.16 | 0.16 | 0.14 | 0.15 | 1.88 |
| 213 | 1.00 | 0.99 | 1.00 | 0.15 | 0.16 | 0.14 | 0.15 | 1.90 |
| 203 | 1.00 | 0.97 | 0.98 | 0.16 | 0.15 | 0.13 | 0.14 | 1.92 |
| 193 | 1.00 | 0.96 | 0.98 | 0.16 | 0.15 | 0.14 | 0.15 | 1.89 |

**Figure S95:** v’ant Hoff plot of **8**.

**8.3.1** **Theoretical Calculations for the PCI of 8**

The geometric structures of PC1 and PC2 were optimized using B3LYP/6-31g**, the calculation was performed according to a literature procedure. ^[S^[^41^](#_ENREF_41)^]^

The relative energy of PC1 compared with PC2 is ΔΔ*G*_f_*_,_*_298_ = -2.4 kcal/mol.

1. **NMR Titration Experiments**

**9.1 General procedure for calculating binding constants with salts:**

By adding guest (G) to an NMR tube with host (H) solution, a binding constant could be determined, similar to the procedure Cram *et al*. used. ^[S^[^42^](#_ENREF_42)^]^

The NMR spectra were recorded until no further changes could be seen to ensure thermodynamic equilibrium. The binding constants were calculated as follows:

$$K_{HG}= \frac{\left[ HG \right]_{e}}{\left[ H \right]_{e}\cdot\left[ G \right]_{e}}$$

With:

$${[G]}_{e} = {[G]}_{i}-{[H]}_{e}$$

$${[H]}_{e} = {[H]}_{i}-{[HG]}_{e}$$

*K_HG_* can be simplified to:

$K_{HG}= \frac{\left[ HG \right]_{e}}{\left[ H \right]_{e}\cdot(\left[ G \right]_{i}-\left[ HG \right]_{e})}$ (eqn. 1)

A linear adjustment was carried out for titration measurements with several values. For this, [HG] was used as the y-value and [H]·[G] as x-value. The slope should correspond to *K* in the linear fitting range.

**9.2 Titration of calixarene 5 with alkali triflates**

**9.2.1 Titration in CDCl_3_**

Calixarene **5** (23.05 mg) was dissolved in 4 mL CDCl_3_ and NaOTf, LiOTf or KOTf were added as salts to the NMR tubes. Because no NMR spectra could be recorded with KOTf, the salt mixture needed to be filtered through a plug of cotton prior to measurement. For calculation of the ratios, the integrals of the aromatic signals were used: Li^+^⸦5: *δ =* 6.60 ppm, Na^+^⸦5: *δ =* 6.62 ppm und 5: *δ =* 6.19 ppm.

Even with 100 equiv. KOTf no uptake of potassium ions was observed in CDCl_3_. The maximum amount of lithium uptake was approx. 93%. The maximum amount of sodium uptake was approx. 82%.

**Figure S96**: ^1^H NMR spectra (CDCl_3_, 300 MHz, 295 K) of **5** with KOTf after filtered through a plug of cotton.

**Figure S97**: ^1^H NMR spectra (CDCl_3_, 300 MHz, 295 K) of **5** with different amounts of LiOTf.

**Figure S98:** ^1^H NMR spectra (CDCl_3_, 300 MHz, 295 K) of **5** with different amounts of NaOTf.

**9.2.2 Titration of calixarene 5 with alkali triflates in THF-*d_8_***

Calixarene **5** was dissolved in THF-*d_8_* and LiOTf, NaOTf or KOTf were added as salts to the NMR tubes. After ultrasonication until everything was dissolved (KOTf did not dissolved completely), ^1^H NMR spectra were recorded.

With LiOTf and KOTf no uptake was observed in THF-*d_8_*. Therefore, a maximum uptake of 1% was assumed, relating to a *K*_a_ < 0.030 M^-1^. The same is true for lithium. *K*_Li_ < 0.03 M^-1^.

(c_i_(calix): 0.0038 M, *c*_i_ KOTf: 0.367 M)

$$K_{Kcalix}< \frac{0.000038}{0.003762\cdot(0.367-0.000038)}=0.03 M^{-1}$$

**Figure S99:** ^1^H NMR (THF-d_8_, 300 MHz, 295 K) of **5** with different amounts of KOTf.

*c*_i_(calix): 0.0038 M, *c*_i_ (LiOTf): 0.378 M

$$K_{Licalix}< \frac{0.000038}{0.003762\cdot(0.378-0.000038)}=0.03 M^{-1}$$

**Figure S100:** ^1^H NMR (THF-d_8_, 300 MHz, 295 K) of **5** with different amounts of LiOTf.

For measurements with NaOTf, calixarene **5** was dissolved in THF-*d_8_* and 1 equiv., 92 equiv., 125 equiv., and 201 equiv. NaOTf were added as salts to the NMR tube. After ultrasonication until everything was dissolved, ^1^H NMR spectra (300 MHz) were recorded. For the calculation, the ratio of the integration of the aromatic signals were used: Na^+^⸦5: *δ=* 6.76 ppm and **5:** *δ =* 6.17 ppm. The *K*_Na_ value for sodium ions in THF-*d_8_* and calixarene is *K*_Na_ = 1.35 ±0.07 M^-1^.

**Table S2:** Values for the titration experiment from 5 in THF-d_8_ with NaOTf.

| Equiv. | [H_0_] (M) | [G_0_](M) | % HG | [HG](M) | [H](M) | [G](M) |
| --- | --- | --- | --- | --- | --- | --- |
| 1 | 3.76·10^-3^ | 3.73·1^-3^ | 0 | - | - | - |
| 92 | 3.76·10^-3^ | 0.345 | 42 | 1.58·10^-3^ | 2.18·10^-3^ | 3.44·10^-1^ |
| 125 | 3.76·10^-3^ | 0.469 | 48 | 1.81·10^-3^ | 1.96·10^-3^ | 4.68·10^-1^ |
| 201 | 3.76·10^-3^ | 0.755 | 58 | 2.18·10^-3^ | 1.58·10^-3^ | 7.53·10^-1^ |

**Figure S101:** ^1^H NMR spectra (THF-d_8_, 300 MHz, 295 K) for the titration experiment of calixarene **5** with NaOTf. For integration the aromatic signals at δ = 6.76 ppm and δ = 6.17 ppm were used.

**Figure S102:** Linear fitting of titration experiments of NaOTf and calixarene **5** in THF-*d_8_*.

- 1. **Titration of nanocone 3 with alkali triflates**

**9.3.1 Titration in CDCl_3_**

Because of high binding constants in CDCl_3_ and insolubility of the triflate salts, no binding constants could be obtained by adding salts to the NMR tube. Therefore, a competition experiment with nanocone **3** and 18-crown-6 and cryptand 2.2.1. (commercially available as Kryptofix221) were performed in CDCl_3_ for LiOTf and KOTf. A measurement with CDCl_3_ saturated with D_2_O was not performed due to results being not promising for these two salts. For the measurements with sodium triflate the solvent was saturated with D_2_O to better compare to literature reported values of 18-crown-6 were measured in CHCl_3_·H_2_O or CDCl_3_·D_2_O. A known amount of lithium triflate complex (Li^+^⸦**3**)OTf, sodium complex (Na^+^⸦**3**)OTf or potassium triflate complex (K^+^⸦**3**)OTf as well as the crown ether were dissolved in CDCl_3_ to get stock solutions.

Note: all NMR spectra from the same experiment were processed in the same way: the phases and the baselines were corrected. The integrals were all drawn equally to avoid deviation within the experiment. Nevertheless, it was found that the measured integrals have an error of about ±5%. This corresponds to an error of approx. ±0.03 equiv. especially when determining the amount of crown ether. The method of Cram ^[S^[^42^](#_ENREF_42)^]^ was used for this experiment and is exemplarily explained for 18-crown-6 and potassium triflate complex (K^+^⸦**3**)OTf.

Preparation: all NMR tubes, vials, separating funnel etc. were washed with dichloromethane, dried, washed with acetone (HPLC grade), MilliQ water and EtOH (HPLC grade) and dried overnight. CDCl_3_ (stored over Ag) was saturated with D_2_O by shaking CDCl_3_ (100 mL) with D_2_O (10 mL) for 2 min. The layers were separated.

A stock solution from potassium triflate complex (K^+^⸦**3**)OTf and 18-crown-6 were prepared in CDCl_3_·D_2_O. Both solutions were mixed in NMR tubes. The tubes were kept in the dark until no changes were observed in the NMR spectra anymore, to ensure thermodynamic equilibrium. The amount of nanocone **3**, K^+^⸦5 and 18-crown-6 were determined by integrating diagnostic NMR signals.

*K*_e_ was determined as follow: (i : initial, e: equilibrium)

*K_e_*= $\frac{\left[ K^{+}⸦18C6 \right]_{e}\cdot\left[ \mathbf{3} \right]_{e}}{\left[ K^{+}⸦3 \right]_{e}\cdot\left[ \mathbf{18C6} \right]_{e}}$

With following assumptions:

$${[K^{+}⸦18C6]}_{e} = {[\mathbf{3}]}_{e}$$

$${[\boldsymbol{18}\boldsymbol{C}\boldsymbol{6}]}_{e} = {[\mathbf{18C6}]}_{i}-{[K^{+}⸦18C6]}_{e}$$

*K*_e_ can be simplified to:

*K_e_*= $\frac{{\left[ \mathbf{3} \right]_{e}}^{2}}{\left[ K^{+}⸦3 \right]_{e}\cdot(\left[ \mathbf{18C6} \right]_{i}-\left[ \mathbf{3} \right]_{e})}$ (eqn. 2)

For 18-crown-6, Stoddart et al. published in 1980 the following values for CDCl_3_·H_2_O: ^[S^[^43^](#_ENREF_43)^]^

Li^+^: 0.43·10^6^ M^-1^ Na^+^: 1.3·10^6^ M^-1^ K^+^: 100,000·10^6^ M^-1^

**9.3.1.a Measurements with potassium triflate:**

**Table S3:** Used equiv. of 18-crown-6 and measured equiv. nanocone **3** for the K^+^⸦3 experiment.

| Equiv. 18-crown-6 measured | Equiv. nanocone **3** measured |
| --- | --- |
| 0.64 | 0.65 |
| 0.75 | 0.76 |
| 0.86 | 0.87 |
| 0.97 | 0.97 |

By adding 64% of 18-crown-6 to potassium triflate complex (K^+^⸦**3**)OTf, 65% nanocone **3** and 35% potassium complex K^+^⸦**3** were obtained. This results could be reproduced with different amounts of 18-crown-6 indicating that the binding constant of nanocone **3** towards potassium is lower than for 18-crown-6 (K < 100,000·10^6^ M^-1^).

**Figure S103**: ^1^H NMR spectra (500 MHz, 295 K, CDCl_3_) from 18-crown-6 and potassium triflate complex K^+^⸦**3** . The equivalents of each substance were determined by integration of the marked signals. *δ* = 3.63 ppm 18-crown-6.

**9.3.1.b Measurements with lithium triflate:**

By adding 59% of 18-crown-6 to lithium triflate complex (Li^+^⸦3)OTf, 59% nanocone **3** and 41% Li^+^⸦3 were obtained. Due to sodium contamination, a signal for sodium complex Na^+^⸦3 was detected in every measurement. With higher amounts of crown ether, less amount of nanocone was detected. The binding constant was estimated to be lower or similar to the one measured with 18-crown-6. According to Stoddart et al. the constant for lithium picrate in CDCl_3_·H_2_O is *K*_a_ = 0.43·10^6^ ^[S^[^43^](#_ENREF_43)^]^

**Table S4:** Measured equiv. of 18-crown-6 and measured equiv. of nanocone **3** for the Li^+^⸦3 experiment.

| Equiv. 18-crown-6 | Amount nanocone **3** |
| --- | --- |
| 0.56 | 0.59 |
| 0.59 | 0.59 |
| 0.66 | 0.66 |
| 0.76 | 0.72 |
|  |  |

**Figure S104**: ^1^H NMR spectra from 18-crown-6 and Li^+^⸦3 (CDCl_3_, 500 MHz). The equivalents of each substance were determined by integration of the marked signals. *δ* = 3.72 ppm 18-crown-6.

**9.3.1.c Measurements with sodium triflate:**

Measurements with NaOTf were performed in CDCl_3_ saturated with D_2_O to get values which are comparable with values reported in the literature.

**Table S5:** Measured equiv. of 18-crown-6 and measured equiv. of nanocone **3** for the Na^+^⸦3 experiment.

| Equiv. 18-crown-6 | Amount nanocone **3** | *K*_e_ |
| --- | --- | --- |
| 6.5 | 0.71 | 0.30 |
| 6.5 | 0.68 | 0.25 |
| 6.7 | 0.72 | 0.31 |
|  |  |  |

Average: *K*_e_= 0.29 or 1/*K*_e_= 3.48.

The *K*_a_ value for nanocone **3** towards 18-crown-6 is estimated to be 3.5 times higher than 1.3·10^6^ M^-1^.

**Figure S105*:*** ^1^H NMR spectra (600 MHz, CDCl_3_·D_2_O, 295K) from 18-crown-6 with Na^+^⸦3 . The equivalents of each substance were determined by integration of the marked signals.

*δ* = 3.68 ppm 18-crown-6.

**Table S6:** Measured equiv. of Kryptofix221 and measured equiv. of nanocone **3** for the Na^+^⸦3 experiment.

| Equiv. Kryptofix221 | Amount nanocone **3** |
| --- | --- |
| 0.42 | 0.42 |
| 0.42 | 0.43 |
| 0.66 | 0.65 |
|  |  |

By adding 0.66 equivs. of Kryptofix 221 to (Na^+^⸦3)OTf, 66% nanocone and 44% Na^+^⸦3 were obtained. The binding constant is estimated to be substantially lower to the one measured with Kryptofix 221. According to Cram the constant for sodium picrate in CDCl_3_·D_2_O is 9.57·10^12^ M^-1^.^[S12],[^[^S42^](#_ENREF_42)^]^. Note: Because of complicated spectra of Kryptofix 221 the determination of equiv. was difficult. The error of integration is approx. ±0.03 equiv.

**Figure S106**: ^1^H NMR spectra (600 MHz, CDCl_3_·D_2_O, 295K) from Kryptofix 221 and Na^+^⸦3 . The equivalents of each substance were determined by integration of the marked signals.

**9.3.2 Titration of nanocone 3 in THF**

**9.3.2.a Measurements with potassium triflate in THF-*d_8_***

Due to insolubility of KOTf in THF, all NMR gave broadened spectra with salt in the NMR tube. Therefore, the salt was filtered off with a plug of cotton. Unfortunately, the NMR spectra still looking broadened. Due to overlapping of the NMR peaks integration is performed as depicted in Figure S107. The association constant for the uptake is *K_K_* = 150 M^-1^.

**Table S7:** Values for KOTf and nanocone **3** for the K^+^⸦3 experiment in THF-*d*_8_.

| Equiv. KOTf | **3** initial | KOTf initial | K^+^⸦3 | *K*_K_ |
| --- | --- | --- | --- | --- |
| 0.60 | 0.0079 | 0.0048 | 0.0022 | 151 |
| 0.68 | 0.0011 | 0.0079 | 0.0040 | 140 |
| 1.64 | 0.0075 | 0.0123 | 0.0045 | 191 |
|  |  |  |  |  |

**Figure S107:** ^1^H NMR spectra (300 MHz, THF-*d_8_*, 295K) from nanocone **3** with different amounts of KOTf. The equivalents of each substance were determined by integration of the marked signals.

**9.3.2.b Measurements with lithium triflate in THF-*d_8_***

Titration experiment were carried out in THF-d_8_ (dried overnight with 4 Å molecular sieves). As internal standard, tetramethylsilane was used. For the first titration experiment, LiOTf (98%) was used and weighed in a glovebox. For the second titration experiment, LiOTf stored under ambient conditions was used (lower sodium contamination). Because of higher binding towards sodium ions, the nanocone **3** binds even residues of sodium ions. For calculating the binding constants with the program bindfit, the initial value of host concentration (titration 1: 4.31·10^-3^ M^-1^, titration 2: 3.78·10^-3^ M^-1^) as well as the corrected values were used by subtracting the amount of formed Na^+^⸦**3** from the initial concentration of **3**. The chemical shifts of the double bound (H^a^: *δ* = 7.04 ppm) the O-CH_2_- group (H^b^: *δ* = 3.84 ppm) and propyl-CH_3_ group (H^c^: *δ* = 1.19 ppm) were plotted using bindfit (http://supramolecular.org) ^[S44,S45]^. To obtain better results, 5 decimal places were used. Both titration experiments were repeated three times (experiment 1: after ~6 h, 4d, 5d, experiment 2: ~6 h, 1d, 2d). Note: only the results with corrected host concentrations are discussed here. The initial concentrations are also plotted, but no significant differences were observed. The bindfit links are included at the end of the SI

**Table S8:** Average value of the binding constant K_a_ and their standard deviation.

| Measurement | Titration 1 | Titration 2 | All values |
| --- | --- | --- | --- |
| 1 | 15.57 | 42.7 |  |
| 2 | 2.45 | 15.15 |  |
| 3 | 2.24 | 13.03 |  |
| Average value | 6.75 | 23.63 | 15.19 |
| **Standard deviation** | 6.23 | 13.51 | 13.49 |

**Titration experiment 1:**

**Table S9**: Association constancies obtained by titration of Nanocone **3** with LiOTf.

| Measurement | K_a_ (M^-1^) | Ka error % | K_a_ error (M^-1^) | SSR |
| --- | --- | --- | --- | --- |
| 1 | 15.57 | 98 | 15.26 | 1.46·10^-5^ |
| 2 | 2.45 | 6.41 | 0.16 | 9.82·10^-6^ |
| 3 | 2.24 | 6.46 | 0.15 | 1.17·10^-5^ |

**Table S10:** Parameters of the titration experiment 1 measurement 1.

| Solution | Host [M] | LiOTf [M] | Chemical shift ppm | | |
| --- | --- | --- | --- | --- | --- |
|  |  |  | H^a^ | H^b^ | H^c^ |
| 0 | 4.31·10^-3^ | 0 | 7.04272 | 3.83987 | 1.18672 |
| 1 | 3.41·10^-3^ | 4.31·10^-3^ | 7.04304 | 3.84009 | 1.18643 |
| 2 | 3.23·10^-3^ | 2.16·10^-2^ | 7.04306 | 3.84137 | 1.18554 |
| 3 | 3.19·10^-3^ | 1.08·10^-1^ | 7.04397 | 3.84135 | 1.18465 |
| 4 | 3.36·10^-3^ | 2.16·10^-1^ | 7.04489 | 3.84339 | 1.18388 |
| 5 | 3.32·10^-3^ | 4.31·10^-1^ | 7.04563 | 3.84209 | 1.18592 |
| 6 | 3.32·10^-3^ | 6.47·10^-1^ | 7.04574 | 3.84238 | 1.18712 |
| 7 | 3.23·10^-3^ | 8.62·10^-1^ | 7.04675 | 3.8438 | 1.18657 |
| std dev |  |  | 0.00140 | 0.00132 | 0.00104 |

**Figure S108**: Fits (left) and residuals (right) of ^1^H NMR titration experiment (300 MHz, THF-d_8_/TMS, 295 K) of Nanocone **3** and LiOTf evaluated by bindfit software. Titration 1, Measurement 1.

**Figure S109:** ^1^H NMR spectra (300 MHz, THF-d_8_/TMS, 295 K) with different equivalents of LiOTf. Titration Experiment 1, measurement 1.

**Figure S110:** ^1^H NMR spectra (300 MHz, THF-d_8_/TMS, 295 K) with different equivalents of LiOTf. Titration Experiment 1, measurement 2.

**Table S11:** Parameters of the titration experiment 1 measurement 2.

| Solution | Host [M] | LiOTf [M] | Chemical shift ppm | | |
| --- | --- | --- | --- | --- | --- |
|  |  |  | H^a^ | H^b^ | H^c^ |
| 0 | 4.31·10^-3^ | 0 | 7.0451 | 3.84078 | 1.18674 |
| 1 | 3.49·10^-3^ | 4.31·10^-3^ | 7.04584 | 3.84129 | 1.18608 |
| 2 | 3.19·10^-3^ | 2.16·10^-2^ | 7.04557 | 3.84266 | 1.18558 |
| 3 | 3.19·10^-3^ | 1.08·10^-1^ | 7.0458 | 3.84965 | 1.18334 |
| 4 | 3.28·10^-3^ | 2.16·10^-1^ | 7.04585 | 3.85649 | 1.18036 |
| 5 | 3.23·10^-3^ | 4.31·10^-1^ | 7.04714 | 3.86291 | 1.17708 |
| 6 | 3.23·10^-3^ | 6.47·10^-1^ | 7.04883 | 3.86525 | 1.17424 |
| 7 | 3.23·10^-3^ | 8.62·10^-1^ | 7.04986 | 3.86983 | 1.17228 |
| std dev |  |  | 0.00161 | 0.01087 | 0.00527 |

**Figure S111:** Fits (left) and residuals (right) of ^1^H NMR titration experiment (300 MHz, THF-d_8_/TMS, 295 K) of Nanocone **3** and LiOTf evaluated by bindfit software. Titration 1, Measurement 2.

**Figure S112**: ^1^H NMR spectra (300 MHz, THF-d_8_/TMS, 295 K) with different equivalents of LiOTf. Titration experiment 1, measurement 3.

**Table S12**: Parameters of the titration experiment 1 measurement 3.

| Solution | Host [M] | LiOTf [M] | Chemical shift ppm | | |
| --- | --- | --- | --- | --- | --- |
|  |  |  | H^a^ | H^b^ | H^c^ |
| 0 | 4.31·10^-3^ | 0 | 7.04531 | 3.84087 | 1.18669 |
| 1 | 3.54·10^-3^ | 4.31·10^-3^ | 7.04648 | 3.84168 | 1.1864 |
| 2 | 3.28·10^-3^ | 2.16·10^-2^ | 7.04576 | 3.84279 | 1.18554 |
| 3 | 3.32·10^-3^ | 1.08·10^-1^ | 7.04595 | 3.84963 | 1.18335 |
| 4 | 3.28·10^-3^ | 2.16·10^-1^ | 7.04648 | 3.85788 | 1.18077 |
| 5 | 3.28·10^-3^ | 4.31·10^-1^ | 7.04745 | 3.86358 | 1.17727 |
| 6 | 3.23·10^-3^ | 6.47·10^-1^ | 7.04912 | 3.86701 | 1.17427 |
| 7 | 3.32·10^-3^ | 8.62·10^-1^ | 7.05 | 3.87232 | 1.1725 |
| std dev |  |  | 0.0160 | 0.0116 | 0.0052 |

**Figure S113:** Fits (left) and residuals (right) of ^1^H NMR titration experiment (300 MHz, THF-d_8_/TMS, 295 K) of Nanocone **3** and LiOTf evaluated by bindfit software. Titration 1, Measurement 3.

**Titration experiment 2:**

**Table S13:** Association constancies obtained by titration of Nanocone **3** with LiOTf. Experiment 2.

| Measurement | K_a_ (M^-1^) | K_a_ error % | K_a_ error (M^-1^) | SSR |
| --- | --- | --- | --- | --- |
| 1 | 42.70 | 22.32 | 9.52 | 4.63·10^-6^ |
| 2 | 15.15 | 14.08 | 2.13 | 6.61·10^-6^ |
| 3 | 13.03 | 14.50 | 1.89 | 6.74·10^-6^ |

**Figure S114**: ^1^H NMR spectra (300 MHz, THF-d_8_/TMS, 295 K) with different equivalents of LiOTf. Titration experiment 2, measurement 1.

**Table S14:** Parameters of the titration experiment 2 measurement 1.

| Solution | Host [M] | LiOTf [M] | Chemical shift ppm | | |
| --- | --- | --- | --- | --- | --- |
|  |  |  | H^a^ | H^b^ | H^c^ |
| 0 | 3.78E-03 | 0 | 7.04307 | 3.8397 | 1.1867 |
| 1 | 3.40·10^-3^ | 3.78·10^-3^ | 7.04302 | 3.84036 | 1.1866 |
| 2 | 3.37·10^-3^ | 7.56·10^-3^ | 7.04343 | 3.841 | 1.18617 |
| 3 | 3.37·10^-3^ | 1.13·10^-2^ | 7.04306 | 3.84137 | 1.1857 |
| 4 | 3.37·10^-3^ | 1.51·10^-2^ | 7.04317 | 3.84212 | 1.18546 |
| 5 | 3.37·10^-3^ | 1.89·10^-2^ | 7.04356 | 3.84293 | 1.18534 |
| 6 | 3.37·10^-3^ | 3.78·10^-2^ | 7.04405 | 3.84575 | 1.1841 |
| 7 | 3.37·10^-3^ | 8.33·10^-2^ | 7.04395 | 3.84483 | 1.18455 |
| 8 | 3.33·10^-3^ | 1.92·10^-1^ | 7.04416 | 3.84624 | 1.18409 |
| std dev |  |  | 0.00043 | 0.00226 | 0.00094 |

**Figure S115:** Fits (left) and residuals (right) of ^1^H NMR titration experiment (300 MHz, THF-d_8_/TMS, 295 K) of Nanocone **3** and LiOTf evaluated by bindfit software. Titration 2, Measurement 1.

**Figure S116:** ^1^H NMR spectra (300 MHz, THF-d_8_/TMS, 295 K) with different equivalents of LiOTf. Titration experiment 2, measurement 2.

**Table S15:** Parameters of the titration experiment 2 measurement 2.

| Solution | Host [M] | LiOTf [M] | Chemical shift ppm | | |
| --- | --- | --- | --- | --- | --- |
|  |  |  | H^a^ | H^b^ | H^c^ |
| 0 | 3.78E-03 | 0 | 7.04307 | 3.8397 | 1.1867 |
| 1 | 3.33·10^-3^ | 3.78·10^-3^ | 7.04301 | 3.84023 | 1.1862 |
| 2 | 3.29·10^-3^ | 7.56·10^-3^ | 7.04344 | 3.84117 | 1.18606 |
| 3 | 3.33·10^-3^ | 1.13·10^-2^ | 7.04354 | 3.84195 | 1.18595 |
| 4 | 3.22·10^-3^ | 1.51·10^-2^ | 7.04408 | 3.84306 | 1.18568 |
| 5 | 3.22·10^-3^ | 1.89·10^-2^ | 7.04412 | 3.84355 | 1.18558 |
| 6 | 3.33·10^-3^ | 3.78·10^-2^ | 7.04424 | 3.84623 | 1.18422 |
| 7 | 3.22·10^-3^ | 8.33·10^-2^ | 7.04488 | 3.84675 | 1.18455 |
| 8 | 3.25·10^-3^ | 1.92·10^-1^ | 7.04531 | 3.85245 | 1.18248 |
| std dev |  |  | 0.00074 | 0.00380 | 0.00123 |

**Figure S117:** Fits (left) and residuals (right) of ^1^H NMR titration experiment (300 MHz, THF-d_8_/TMS, 295 K) of Nanocone **3** and LiOTf evaluated by bindfit software. Titration 2, Measurement 2.

**Figure S118:** ^1^H NMR spectra (300 MHz, THF-d_8_/TMS, 295 K) with different equivalents of LiOTf. Titration experiment 3, measurement 1.

**Table S16**: Parameters of the titration experiment 2 measurement 3.

| Solution | Host [M] |  | LiOTf [M] | Chemical shift ppm | | |
| --- | --- | --- | --- | --- | --- | --- |
|  |  |  |  | H^a^ | H^b^ | H^c^ |
| 0 | 3.78·10^-3^ |  | 0 | 7.04435 | 3.84049 | 1.1868 |
| 1 | 3.37·10^-3^ |  | 3.78·10^-3^ | 7.04381 | 3.84094 | 1.18642 |
| 2 | 3.37·10^-3^ |  | 7.56·10^-3^ | 7.044 | 3.84182 | 1.18641 |
| 3 | 3.29·10^-3^ |  | 1.13·10^-2^ | 7.04398 | 3.84236 | 1.18576 |
| 4 | 3.29·10^-3^ |  | 1.51·10^-2^ | 7.04428 | 3.84323 | 1.18566 |
| 5 | 3.33·10^-3^ |  | 1.89·10^-2^ | 7.04401 | 3.84364 | 1.18515 |
| 6 | 3.29·10^-3^ |  | 3.78·10^-2^ | 7.0449 | 3.84697 | 1.18463 |
| 7 | 3.22·10^-3^ |  | 8.33·10^-2^ | 7.04499 | 3.84743 | 1.18451 |
| 8 | 3.29·10^-3^ |  | 1.92·10^-1^ | 7.04573 | 3.85254 | 1.18235 |
| std dev |  |  |  | 0.00060 | 0.00368 | 0.00129 |

**Figure S119**: Fits (left) and residuals (right) of ^1^H NMR titration experiment (300 MHz, THF-d_8_/TMS, 295 K) of Nanocone **3** and LiOTf evaluated by bindfit software. Titration 2, Measurement 3.

**Determination of the standard deviation of the NMR machine (Bruker Avance III 300 MHz)**

To determine the displacement error of the NMR device, the blank tubes from experiments 1 and 2 (only pure compound **3** in THF-d_8_/TMS, (titration 1: 4.31·10^-3^ M^-1^, titration 2: 3.78·10^-3^ M^-1^) were measured four times (M1-4) over a period of 1-6 days. The shift (ppm) of H^a^, H^b^ and H^c^ were used to calculated the standard derivation. The standard deviation and the shifts are listed in Table S9. The standard deviation is between 0.00013 and 0.0015 ppm.

**Table S17:** Chemical shifts in ppm of Nanocone **3** in THF-d_8_ with TMS measured 4 times

|  | Chemical shift ppm | | |
| --- | --- | --- | --- |
| Experiment 1 | H^a^ | H^b^ | H^c^ |
| M1 | 7.04259 | 3.83986 | 1.18676 |
| M2 | 7.04445 | 3.84036 | 1.18647 |
| M3 | 7.04499 | 3.84078 | 1.18683 |
| M4 | 7.04531 | 3.84092 | 1.18675 |
| **Average value** | **7.04434** | **3.8405** | **1.18670** |
| **Standard deviation** | **0.00105** | **0.00041** | **0.00014** |
| Experiment 2 | H^a^ | H^b^ | H^c^ |
| M1 | 7.04308 | 3.83972 | 1.18669 |
| M2 | 7.04363 | 3.83998 | 1.18681 |
| M3 | 7.04387 | 3.84025 | 1.18698 |
| M4 | 7.04425 | 3.84039 | 1.1867 |
| Average value | 7.04371 | 3.84009 | 1.18680 |
| **Standard deviation** | **0.00042** | **0.00026** | **0.00012** |

**Figure S120**: ^1^H NMR spectra (300 MHz, THF-d_8_/TMS, 295 K) of pure Nanocone **3** to determine the standard deviation of the NMR shift (Bruker Avance III 300 MHz). Concentrations: titration experiment 1: 4.31·10^-3^ M^-1^, titration experiment 2: 3.78·10^-3^ M^-1^.

**9.3.2.c Measurements with sodium triflate in THF-*d_8_***

**Table S18:** Values for NaOTf and nanocone **3** for the Na^+^⸦3 experiment in THF-*d*_8_.

| Equiv. NaOTf | **3** initial | NaOTf initial | Na^+^⸦3 | *K*_Na_ |
| --- | --- | --- | --- | --- |
| 0.65 | 0.0038 | 0.0025 | 0.0020 | 2248 |
| 1.0 | 0.005 | 0.005 | 0.0040 | 2801 |

The constant for sodium binding in THF is *K* = 2.5·10^3^ M^-1^.

**Figure S121:** ^1^H NMR spectra (300 MHz, THF-*d_8_*, 295*K*) from nanocone **3** with different amounts of NaOTf. The equivalents of each substance were determined by integration of the marked signals.

**9.3.2.d Separation of lithium and sodium triflate:**

A mixture of LiOTf (39.5 mg) and NaOTf (15.6 mg) was prepared and dissolved in THF. Nanocone **3** was added and waited for 5 min. The solvent was evaporated and to the remaining solid was added CDCl_3_. The mixture was filtered through a syringe filter. An NMR spectrum was measured, in which only signals from Na^+^⸦3 were visible. Therefore, a full separation of lithium and sodium salts is possible with nanocone **3** in THF and CHCl_3_. Note: THF signals were visible. Probably because the THF was only evaporated with an ordinary rotary evaporator.

**Figure S122:** ^1^H NMR spectra (300 MHz, CDCl_3_, 295 K) of separation experiment between LiOTf and NaOTf with nanocone **3**. The equivalents of each substance were determined by integration of the marked signals.

**9.4** Competition reaction between salts and nanocone **3**

In order to obtain a comparative value between the respective salts, competition experiments were carried out. Mixtures of LiOTf/KOTf, LiOTf/NaOTf and NaOTf/KOTf were prepared for this purpose. The mixtures were prepared in such a way that the same equiv. of salt was present in each case. The salts were mixed with a spatula to homogenise it as much as possible. A quantity **X** of each mixture was weighed into an NMR tube using a balance with an accuracy of 2 decimal places (X.XX mg). A stock solution of nanocone **3** was added to this mixture so that the following equivalents were achieved: 1 equiv. salt A, 1 equiv. salt B, 0.8 equiv. nanocone **3**. The stock solutions were prepared in CDCl_3_ or CDCl_3_·D_2_O, to determine if the water has any strong effect. It was shown, that there is no difference in the results with CDCl_3_ or CDCl_3_·D_2_O. The experiments were repeated in the glovebox (N_2_-atmosphere, <0.1 ppm water, <0.1 ppm O_2_). The balance of the glovebox has an accuracy of one decimal place. The NMR tube was weighed 3 times to ensure the most accurate weighing possible. The sealed tubes with salt mixtures were removed from the glovebox and a stock solution of cone **3** in CDCl_3_·D_2_O was added to obtain a ratio of 1:1:0.8 again.

The salts used are hardly soluble in CDCl_3_·D_2_O. The results from the glovebox weights and the measurements made in air at ambient laboratory operation do not deviate significantly, concluding that influence of humidity was negligible. All NMR tubes were measured until no further changes could be seen (equilibrium after approx. 1-2 weeks).

For every salt mixture only two NMR spectra of the full series are depicted below, measured on a 600 MHz NMR spectrometer.

The relative *K* values are calculated as follows: (e: equilibrium, i: initial)

*K_Na_*= $\frac{\left[ \mathrm{Na}^{+}⸦3 \right]_{e}}{\left[ \mathbf{3} \right]_{e}\cdot\left[ NaOTf \right]_{e}}$

*K_Li_*= $\frac{\left[ \mathrm{Li}^{+}⸦3 \right]_{e}}{\left[ \mathbf{3} \right]_{e}\cdot\left[ LiOTf \right]_{e}}$

*K_Li_ /K_Na_*= $\frac{\left[ \mathrm{Li}^{+}⸦3 \right]_{e}\cdot\left[ NaOTf \right]_{e}}{\left[ \mathrm{Na}^{+}⸦3 \right]_{e}\cdot\left[ LiOTf \right]_{e}}$

For simplifying the relative *K* values were calculated with following initial values:

[LiOTf]_i_ = 1 M, [NaOTf] _i_ = 1 M, [**3**] _i_ = 0.8 M

[LiOTf]_e_ = [LiOTf]_i_ -[ Li^+^⸦3 ]_e_

[NaOTf]_e_ = [NaOTf]_i_-[Na^+^⸦3 ]_e_

**9.4.1 Measurements with LiOTf vs NaOTf:**

**Table S19:** Values for the measurement of NaOTf (1 equiv.) and LiOTf (1 equiv.) with nanocone **3** (0.8 equiv.) in CDCl_3_·D2O.

| NMR | Li^+^⸦3 | Na^+^⸦3 | NaOTf | LiOTf | *K*_Li_/*K*_Na_ |
| --- | --- | --- | --- | --- | --- |
| 1 | 3%, 0.024 M | 97% 0.72 M | 0.224 M | 0.976 M | 0.007 |
| 2 | 3%, 0.024 M | 97% 0.72 M | 0.224 M | 0.976 M | 0.007 |

*K*_Na_ is around or more than 140 times higher then *K*_Li._

**Figure S123:** ^1^H NMR spectra (295 K, 600 MHz) of nanocone **3** (0.8 equiv.) and LiOTf (1 equiv.) and NaOTf (1 equiv.) in CDCl_3_·D_2_O. The equivalents of each substance were determined by integration of the marked signals.

**9.4.2 Measurements with KOTf vs NaOTf:**

**Table S20:** Values for the measurement of NaOTf (1 equiv.) and KOTf (1 equiv.) with nanocone **3** (0.8 equiv.) in CDCl_3_·D2O.

| NMR | K^+^⸦3 | Na^+^⸦3 | KOTF | NaOTf | *K_K_*/*K*_Na_ |
| --- | --- | --- | --- | --- | --- |
| 1 | 10%, 0.08 M | 90% 0.72 M | 0.92 M | 0.28 M | 0.033 |
| 2 | 6% 0.048 M | 94% 0.752 M | 0.952 | 0.248 M | 0.017 |

*K*_Na_ is approx. 30 times higher than *K*_K._

**Figure S124:** ^1^H NMR spectra (295 K, 600 MHz) of nanocone **3** (0.8 equiv.) and NaOTf (1 equiv.) and KOTf (1 equiv.) in CDCl_3_·D_2_O. The equivalents of each substance were determined by integration of the marked signals.

**9.4.3 Measurements with LiOTf vs KOTf**

**Table S21:** Values for the measurement of LiOTf (1 equiv.) and KOTf (1 equiv.) with nanocone **3** (0.8 equiv.) in CDCl_3_·D2O.

| NMR | K^+^⸦3 | Li^+^⸦3 | KOTF | LiOTf | *K*_Li_/*K*_K_ |
| --- | --- | --- | --- | --- | --- |
| 1 | 55%, 0.44 M | 45% 0.36 M | 0.56 M | 0.64 M | 1.4 |
| 2 | 58%, 0.464 M | 42% 0.336 M | 0.536 M | 0.664 M | 1.7 |

*K_K_* is 1.6 times higher than *K*_Li._

**Figure S125:** ^1^H NMR spectra (295 K, 600 MHz) of nanocone **3** (0.8 equiv.) and LiOTf (1 equiv.) and KOTf (1 equiv.) in CDCl_3_·D_2_O. The equivalents of each substance were determined by integration of the marked signals.

**9.5 Nanocone 3 in CDCl_3_ and CDCl_3_·D_2_O**

**Figure S 126:** Comparison of ^1^H NMR spectra Na^+^⸦3 synthesized and measured with CDCl_3_ (top) and CDCl_3_·D_2_O (bottom) (300 MHz, 298 K).

**9.6 Nanocone 3 with NaClO_4_ and NaOTf:**

To study the impact of the anion on sodium binding, nanocone **3** was dissolved in CDCl_3_ and NaClO_4_ was added.

**Figure S127**: Comparison of ^1^H NMR spectra (300 MHz, 295 K) of nanocone **3** with NaClO_4_ (top) and NaOTf (bottom) as salts in CDCl_3_.Because the chemical shifts are equal, the anions are not coordinated in solution. From the NMR tube of NaClO_4_ a crystal could be obtained suitable for x-ray structure analyses.

**9.7 NMR spectra of reduced cone 13 and NaOTf, LiOTf and KOTf**

**Figure S128:** Comparison of NMR spectra (300 MHz, CDCl_3_) of reduced nanocone 13 (top) and the corresponding Li^+^- (2nd) and Na^+^-complexes (3rd) and K^+^- (bottom) complex.

- 1. **Titration experiment between calixarene 5 and nanocone 3**

The nanocone triflate complexes were dissolved in CDCl_3_·D_2_O and calixarene **5** was added. These experiments are similar to the competing experiments between nanocone **3** and crown-ethers (see page 78). The ratio between both substances were calculated by the ratio of their integrals.

**9.8.1 Measurements with potassium triflate:**

It is known that calixarene **5** cannot take up potassium ions. Nevertheless, it was tested if something is happening by mixing K^+^⸦**3** with calixarene by adding 1.6 equiv. calixarene. In the NMR spectra no signals of K^+^⸦**5** were detected. But a small signal for the Na^+^⸦**3.**

**Figure S129:** ^1^H NMR spectra (300 MHz, CDCl_3_, 295K) from calixarene **5** with K^+^⸦**3**·OTf **16**. The equivalents of each substance were determined by integration of the marked signals.

**9.8.2 Measurements with sodium triflate**

The same experiment was repeated with sodium triflate: A large excess of calixarene was needed to determine the binding constant. Therefore, the signals of the nanocone **3** and the Na·cone (Na^+^⸦**3**) are in the baseline of the calixarene **5** signals. 75 equiv. and 136 equiv. of calixarene were added to(Na^+^⸦**3**)OTf. Nanocone **3** is 2.5·10^3^ better than calixarene **5** for binding sodium ions.

**Table S22:** Values for the measurement of (Na^+^⸦**3**) with calixarene **5** in CDCl_3_·D_2_O.

| NMR | **5** | Na^+^⸦**3** | **3** | *K*_e_ | *1/ K*_e_ |
| --- | --- | --- | --- | --- | --- |
| 1 | 136 equiv. | 78% | 22% | 4.5·10^-4^ | 2.2·10^3^ |
| 2 | 75 equiv. | 86% | 14% | 3.0·10^-4^ | 3.3·10^3^ |

**Figure S130:** ^1^H NMR spectra (300 MHz, CDCl_3_, 295K) from calixarene **5** with (Na^+^⸦**3**)·OTf. The equivalents of each substance were determined by integration of the marked signals.

**Figure S131:** ^1^H NMR spectra (300 MHz, CDCl_3_, 295K) from calixarene **5** with Na^+^⸦**3**·OTf **15**. The equivalents of each substance were determined by integration of the marked signals.

**10 Quantum Chemical Calculations**

**Figure S132:** HOMO/HOMO-1 and LUMO/LUMO+1 from Cone **5** and redcone **13**.

**Table S23:** NICS values from **5** and **13**. NICS-inside the cavity, NICS outside the cavity and NICS-0 values.

|  | NICS | A | B | C | D | E | F | G | H |
| --- | --- | --- | --- | --- | --- | --- | --- | --- | --- |
| **5** | inside | -14.5 | -6.5 | -14.5 | -6.5 | -14.5 | -6.5 | -14.5 | -6.5 |
|  | 0 | -13.8 | -1.4 | -13.8 | -1.4 | -13.8 | -1.4 | -13.8 | -1.4 |
|  | outside | -12.3 | -3.1 | -12.3 | -3.1 | -12.3 | -3.1 | -12.3 | -3.1 |
| **13** | inside | -13.7 | -5.0 | -13.5 | -5.2 | -13.6 | -9.6 | -13.5 | -5.2 |
|  | 0 | -13.4 | -0.2 | -13.4 | 0.3 | -13.4 | 0.3 | -13.3 | 0.3 |
|  | outside | -12.4 | 0.6 | -12.4 | -0.0 | -12.5 | -0.0 | -12.3 | -0.1 |

**Nanocone 3:**

| atom | X | Y | Z |
| --- | --- | --- | --- |
| C | -0.06969 | -2.94405 | 1.23132 |
| C | 0.57732 | -2.52279 | 2.53707 |
| C | -1.41514 | -3.37073 | 1.23629 |
| C | 0.60276 | -2.81027 | 0.00788 |
| C | -0.06965 | -2.95099 | -1.21479 |
| C | -1.41506 | -3.37772 | -1.21737 |
| C | 0.60271 | 0.00789 | 2.81022 |
| C | -0.06976 | 1.23131 | 2.94404 |
| C | -1.4152 | 1.23627 | 3.37074 |
| C | -1.41507 | -1.21739 | 3.37777 |
| C | -0.06968 | -1.21479 | 2.95099 |
| O | 1.94728 | 0.00663 | 2.4826 |
| O | 1.94738 | -2.48283 | 0.00658 |
| C | -1.41516 | 3.3777 | 1.21735 |
| C | -0.06975 | 2.95099 | 1.2148 |
| C | 0.6027 | 2.81028 | -0.00785 |
| C | -0.06972 | 2.94407 | -1.23131 |
| C | -1.41518 | 3.37072 | -1.23631 |
| O | 1.94731 | 2.48284 | -0.00651 |
| C | 0.60276 | -0.00786 | -2.81021 |
| C | -0.06965 | 1.21481 | -2.95098 |
| C | -1.41504 | 1.21737 | -3.37777 |
| C | -1.41511 | -1.23629 | -3.37075 |
| C | -0.06967 | -1.2313 | -2.94403 |
| O | 1.94734 | -0.00655 | -2.48263 |
| C | 0.57733 | -2.53707 | -2.52278 |
| C | 0.57731 | 2.52281 | -2.53705 |
| C | 0.57722 | 2.53709 | 2.5228 |
| C | -2.03752 | -3.66932 | 0.0103 |
| C | -2.03749 | -0.0103 | -3.66938 |
| C | -2.03756 | 0.01027 | 3.66936 |
| C | -2.03761 | 3.6693 | -0.01033 |
| O | -3.32 | -4.19391 | 0.01181 |
| O | -3.32003 | 0.01174 | 4.19396 |
| O | -3.31996 | -0.01181 | -4.19399 |
| O | -3.32009 | 4.19386 | -0.01187 |
| C | -2.20067 | 2.47973 | 3.42003 |
| C | -2.2006 | 3.42003 | -2.4798 |
| C | -2.20054 | -2.47977 | -3.42006 |
| C | -2.20057 | -3.42006 | 2.47977 |
| C | -2.20055 | 3.43398 | 2.46057 |
| C | -2.20043 | -2.46063 | 3.43406 |
| C | -2.20042 | -3.43402 | -2.4606 |
| C | -2.20042 | 2.46059 | -3.43408 |
| C | 2.80784 | -3.62982 | 0.01411 |
| C | 4.253 | -3.15592 | 0.00259 |
| C | 5.24657 | -4.32128 | 0.0163 |
| C | 2.80796 | -0.01399 | -3.62949 |
| C | 4.25305 | -0.00264 | -3.15536 |
| C | 5.2468 | -0.0162 | -4.32055 |
| C | 2.80793 | 0.01409 | 3.62944 |
| C | 4.253 | 0.0028 | 3.15526 |
| C | 5.2468 | 0.0164 | 4.32041 |
| C | 2.80779 | 3.62981 | -0.01399 |
| C | 4.25294 | 3.15588 | -0.0024 |
| C | 5.24653 | 4.32121 | -0.01604 |
| C | -4.37974 | 0.00847 | 3.23753 |
| C | -4.3797 | -3.23747 | 0.00862 |
| C | -4.37968 | -0.00858 | -3.23758 |
| C | -4.37978 | 3.23739 | -0.00872 |
| H | 0.4096 | -3.28669 | 3.30526 |
| H | 1.64558 | -2.37841 | 2.39212 |
| H | 0.40961 | -3.30525 | -3.28669 |
| H | 1.6456 | -2.39213 | -2.37841 |
| H | 0.40959 | 3.28671 | -3.30525 |
| H | 1.64557 | 2.37846 | -2.39208 |
| H | 0.40946 | 3.30527 | 3.28671 |
| H | 1.64549 | 2.39218 | 2.37845 |
| H | -2.91189 | 2.56793 | 4.2375 |
| H | -2.91183 | 4.23749 | -2.56801 |
| H | -2.91175 | -2.56798 | -4.23755 |
| H | -2.91179 | -4.23753 | 2.56797 |
| H | -2.91165 | 4.25203 | 2.5443 |
| H | -2.91151 | -2.54439 | 4.25212 |
| H | -2.91151 | -4.25208 | -2.54435 |
| H | -2.91149 | 2.54433 | -4.25216 |
| H | 2.60703 | -4.24147 | 0.90713 |
| H | 2.59865 | -4.25954 | -0.86424 |
| H | 4.41512 | -2.50601 | 0.86987 |
| H | 4.40984 | -2.53152 | -0.88421 |
| H | 5.11631 | -4.96946 | -0.8576 |
| H | 5.1228 | -4.94292 | 0.91018 |
| H | 6.27829 | -3.95739 | 0.007 |
| H | 2.60717 | -0.9069 | -4.2413 |
| H | 2.59893 | 0.86447 | -4.25912 |
| H | 4.415 | -0.87004 | -2.50558 |
| H | 4.40982 | 0.88403 | -2.53077 |
| H | 5.12308 | -0.90996 | -4.94239 |
| H | 6.27847 | -0.00703 | -3.9565 |
| H | 5.11671 | 0.85783 | -4.9686 |
| H | 2.60712 | 0.907 | 4.24125 |
| H | 2.59895 | -0.86437 | 4.25908 |
| H | 4.4149 | 0.87021 | 2.50547 |
| H | 4.40979 | -0.88386 | 2.53066 |
| H | 5.12307 | 0.91015 | 4.94226 |
| H | 6.27845 | 0.00727 | 3.95632 |
| H | 5.11676 | -0.85764 | 4.96846 |
| H | 2.59857 | 4.25952 | 0.86437 |
| H | 2.60703 | 4.24148 | -0.907 |
| H | 4.40972 | 2.53146 | 0.88438 |
| H | 4.41508 | 2.50599 | -0.86969 |
| H | 5.11625 | 4.96938 | 0.85787 |
| H | 6.27825 | 3.95731 | -0.0067 |
| H | 5.12282 | 4.94289 | -0.90992 |
| H | -5.31136 | 0.00937 | 3.80797 |
| H | -4.34331 | 0.9004 | 2.60108 |
| H | -4.34194 | -0.88682 | 2.60593 |
| H | -5.31133 | -3.8079 | 0.00953 |
| H | -4.34323 | -2.60106 | 0.90058 |
| H | -4.34193 | -2.60583 | -0.88664 |
| H | -5.31129 | -0.00949 | -3.80803 |
| H | -4.34323 | -0.90051 | -2.60114 |
| H | -4.34191 | 0.8867 | -2.60597 |
| H | -5.31141 | 3.80781 | -0.00965 |
| H | -4.34326 | 2.601 | -0.90068 |
| H | -4.34201 | 2.60575 | 0.88653 |

**Redcone 13**

| atom | X | Y | Z |
| --- | --- | --- | --- |
| C | -0.03139 | -2.66285 | -1.78749 |
| C | -0.83875 | -2.02975 | -2.91562 |
| C | 1.35519 | -2.8737 | -1.90328 |
| C | -0.64873 | -2.86564 | -0.54312 |
| C | 0.08019 | -3.16032 | 0.61988 |
| C | 1.46767 | -3.37288 | 0.51988 |
| C | -0.8639 | 0.50048 | -2.6403 |
| C | -0.21506 | 1.74291 | -2.68009 |
| C | 1.03352 | 1.85188 | -3.31281 |
| C | 0.98829 | -0.54299 | -3.80585 |
| C | -0.26024 | -0.64791 | -3.1709 |
| O | -2.06974 | 0.38751 | -1.97548 |
| O | -2.02935 | -2.73827 | -0.4524 |
| C | 1.45881 | 3.22999 | -0.64267 |
| C | 0.06363 | 3.04461 | -0.6142 |
| C | -0.56402 | 2.77519 | 0.6133 |
| C | 0.16568 | 2.55402 | 1.7947 |
| C | 1.56015 | 2.74296 | 1.7821 |
| O | -1.94766 | 2.6614 | 0.64293 |
| C | -0.62288 | -0.60889 | 2.72326 |
| C | 0.04829 | 0.53058 | 3.18921 |
| C | 1.34874 | 0.40706 | 3.70334 |
| C | 1.30973 | -1.98838 | 3.21169 |
| C | 0.00767 | -1.86061 | 2.70067 |
| O | -1.89054 | -0.47635 | 2.18882 |
| C | -0.61296 | -3.04158 | 1.97283 |
| C | -0.53598 | 1.91936 | 2.99203 |
| C | -0.7476 | 2.93317 | -1.90034 |
| C | 2.06849 | -3.23367 | -0.74491 |
| C | 1.95371 | -0.85878 | 3.73982 |
| C | 1.60706 | 0.713 | -3.89918 |
| C | 2.17096 | 3.08647 | 0.56185 |
| O | 3.43755 | -3.48558 | -0.86011 |
| O | 2.78867 | 0.84042 | -4.62362 |
| O | 3.19521 | -1.00465 | 4.35154 |
| O | 3.54883 | 3.31544 | 0.54954 |
| C | 1.71568 | 3.19784 | -3.29779 |
| C | 2.4299 | 2.65742 | 3.03495 |
| C | 1.96613 | -3.3454 | 3.13811 |
| C | 2.10694 | -2.79949 | -3.23084 |
| C | 2.22396 | 3.65313 | -1.8957 |
| C | 1.62028 | -1.80829 | -4.33217 |
| C | 2.33572 | -3.81308 | 1.69698 |
| C | 2.04281 | 1.66241 | 4.1717 |
| C | -2.72218 | -3.97053 | -0.68563 |
| C | -4.21882 | -3.72323 | -0.56565 |
| C | -5.03873 | -4.99495 | -0.80415 |
| C | -2.96072 | -0.6299 | 3.12516 |
| C | -4.26706 | -0.37604 | 2.38741 |
| C | -5.49172 | -0.51957 | 3.29494 |
| C | -3.23116 | 0.62728 | -2.77461 |
| C | -4.44527 | 0.57268 | -1.85902 |
| C | -5.75833 | 0.81154 | -2.6092 |
| C | -2.63862 | 3.7816 | 1.2176 |
| C | -2.77697 | 4.97086 | 0.26829 |
| C | -3.53994 | 6.13145 | 0.91567 |
| C | 3.99037 | 0.66377 | -3.88447 |
| C | 4.26312 | -2.3411 | -0.66246 |
| C | 4.32578 | -0.84544 | 3.50407 |
| C | 4.33354 | 2.15635 | 0.28217 |
| H | -0.81271 | -2.64148 | -3.8242 |
| H | -1.8742 | -1.94364 | -2.59523 |
| H | -0.51483 | -3.96158 | 2.55965 |
| H | -1.67152 | -2.85908 | 1.80473 |
| H | -0.42279 | 2.5267 | 3.89721 |
| H | -1.59706 | 1.83691 | 2.7732 |
| H | -0.68713 | 3.85149 | -2.49494 |
| H | -1.78939 | 2.7733 | -1.6376 |
| H | 1.01969 | 3.94817 | -3.68917 |
| H | 2.5636 | 3.18944 | -3.98616 |
| H | 2.48691 | 3.66284 | 3.47522 |
| H | 3.45069 | 2.44056 | 2.72057 |
| H | 1.29672 | -4.0831 | 3.59452 |
| H | 2.87343 | -3.34929 | 3.74607 |
| H | 2.12004 | -3.80741 | -3.6688 |
| H | 3.15316 | -2.58456 | -3.01303 |
| H | 2.28326 | 4.75058 | -1.90058 |
| H | 3.25776 | 3.3263 | -1.78362 |
| H | 0.89789 | -2.31751 | -4.97982 |
| H | 2.46968 | -1.5578 | -4.97142 |
| H | 2.37061 | -4.91155 | 1.6996 |
| H | 3.36194 | -3.50942 | 1.49001 |
| H | 1.39173 | 2.17366 | 4.88975 |
| H | 2.94736 | 1.3993 | 4.72433 |
| H | -2.47312 | -4.35598 | -1.68622 |
| H | -2.39531 | -4.7271 | 0.0442 |
| H | -4.50588 | -2.94732 | -1.28512 |
| H | -4.42988 | -3.31868 | 0.43129 |
| H | -4.78534 | -5.77645 | -0.07911 |
| H | -4.86234 | -5.40294 | -1.80567 |
| H | -6.11023 | -4.794 | -0.71289 |
| H | -2.94374 | -1.64346 | 3.55581 |
| H | -2.83832 | 0.07869 | 3.95905 |
| H | -4.32635 | -1.07409 | 1.54488 |
| H | -4.22485 | 0.6281 | 1.95004 |
| H | -5.56046 | -1.52729 | 3.72038 |
| H | -6.41617 | -0.33204 | 2.74041 |
| H | -5.45818 | 0.18898 | 4.13046 |
| H | -3.15651 | 1.60953 | -3.26621 |
| H | -3.30429 | -0.12977 | -3.57146 |
| H | -4.30901 | 1.31795 | -1.06729 |
| H | -4.45651 | -0.40404 | -1.36224 |
| H | -5.7719 | 1.79514 | -3.09255 |
| H | -6.61391 | 0.76702 | -1.92847 |
| H | -5.91804 | 0.0589 | -3.38982 |
| H | -2.13682 | 4.09895 | 2.14253 |
| H | -3.62833 | 3.3981 | 1.49004 |
| H | -1.77819 | 5.30459 | -0.03626 |
| H | -3.29304 | 4.64178 | -0.64129 |
| H | -3.02521 | 6.4975 | 1.81115 |
| H | -3.63817 | 6.97319 | 0.22377 |
| H | -4.55023 | 5.82959 | 1.21488 |
| H | 4.81488 | 0.79937 | -4.58887 |
| H | 4.0851 | 1.40297 | -3.07846 |
| H | 4.05613 | -0.34144 | -3.4484 |
| H | 4.1312 | -1.91047 | 0.33822 |
| H | 5.2973 | -2.67477 | -0.77854 |
| H | 4.05168 | -1.55346 | -1.39662 |
| H | 5.20988 | -0.99268 | 4.1294 |
| H | 4.33483 | -1.58615 | 2.69399 |
| H | 4.36521 | 0.15866 | 3.06223 |
| H | 5.37937 | 2.47308 | 0.29942 |
| H | 4.17856 | 1.37553 | 1.03739 |
| H | 4.10177 | 1.72381 | -0.69934 |

**calixarene 5**

| Atom | X | Y | z |
| --- | --- | --- | --- |
| C | -2.95247 | -1.71163 | 0.73559 |
| C | -2.35344 | -1.72675 | 2.13698 |
| C | -3.60105 | -2.85072 | 0.2359 |
| C | -2.74803 | -0.61593 | -0.11717 |
| C | -3.03506 | -0.70684 | -1.49327 |
| C | -3.6755 | -1.8523 | -1.96568 |
| C | -3.99156 | -2.9097 | -1.10458 |
| C | 0.06904 | -0.95353 | 2.50236 |
| C | 1.44623 | -1.10817 | 2.28383 |
| C | 1.91696 | -2.30496 | 1.72534 |
| C | 1.0292 | -3.3203 | 1.37266 |
| C | -0.34493 | -3.12112 | 1.52835 |
| C | -0.84245 | -1.94304 | 2.07927 |
| O | -0.40736 | 0.19345 | 3.12872 |
| O | -2.18268 | 0.54178 | 0.37961 |
| C | 4.4928 | -0.05745 | 1.06543 |
| C | 3.1938 | 0.41417 | 1.31139 |
| C | 2.55066 | 1.17079 | 0.32499 |
| C | 3.12752 | 1.35921 | -0.94482 |
| C | 4.41783 | 0.88064 | -1.16692 |
| C | 5.11456 | 0.19955 | -0.15984 |
| O | 1.30198 | 1.69676 | 0.58814 |
| C | -0.11447 | 1.19698 | -2.45635 |
| C | 1.26227 | 0.9538 | -2.57034 |
| C | 1.69898 | -0.27496 | -3.08433 |
| C | 0.77752 | -1.25242 | -3.45709 |
| C | -0.5875 | -1.02648 | -3.26033 |
| C | -1.05071 | 0.18443 | -2.75112 |
| O | -0.56002 | 2.4454 | -2.03458 |
| C | -2.54069 | 0.35961 | -2.45847 |
| C | 2.30126 | 1.95689 | -2.07424 |
| C | 2.44022 | 0.01655 | 2.57187 |
| C | -3.07013 | 1.36083 | 1.15761 |
| C | -3.85648 | 2.36215 | 0.31363 |
| C | -4.74343 | 3.26498 | 1.17753 |
| C | -0.78358 | 3.35864 | -3.11628 |
| C | -1.23311 | 4.69697 | -2.54783 |
| C | -1.47948 | 5.73951 | -3.64275 |
| C | 1.35669 | 3.03885 | 1.09115 |
| C | -0.05565 | 3.4912 | 1.41606 |
| C | -0.10297 | 4.9382 | 1.9149 |
| C | -0.47543 | 0.07425 | 4.55523 |
| C | -1.01793 | 1.37425 | 5.1315 |
| C | -1.12426 | 1.33372 | 6.659 |
| O | -4.653 | -3.964 | -1.67742 |
| O | 1.10783 | -2.47012 | -3.99194 |
| O | 6.38609 | -0.19671 | -0.48529 |
| O | 1.39861 | -4.52964 | 0.84433 |
| C | -4.94499 | -5.08785 | -0.86634 |
| C | 2.48085 | -2.76986 | -4.16324 |
| C | 7.14075 | -0.88727 | 0.49405 |
| C | 2.77448 | -4.7551 | 0.59877 |
| H | -2.81876 | -2.53746 | 2.70951 |
| H | -2.55499 | -0.79645 | 2.66735 |
| H | -3.77641 | -3.68683 | 0.90348 |
| H | -3.93567 | -1.94851 | -3.01546 |
| H | 2.97986 | -2.41006 | 1.54456 |
| H | -1.02022 | -3.8956 | 1.17971 |
| H | 4.99965 | -0.62309 | 1.83936 |
| H | 4.90677 | 1.02266 | -2.12617 |
| H | 2.76454 | -0.45816 | -3.15582 |
| H | -1.27894 | -1.8314 | -3.48602 |
| H | -2.69795 | 1.35437 | -2.04424 |
| H | -3.11289 | 0.28664 | -3.3908 |
| H | 1.78578 | 2.85891 | -1.74296 |
| H | 2.97265 | 2.23856 | -2.89377 |
| H | 3.16099 | -0.3077 | 3.33247 |
| H | 1.8923 | 0.86849 | 2.97561 |
| H | -3.76955 | 0.72828 | 1.72084 |
| H | -2.43399 | 1.88149 | 1.87687 |
| H | -3.14624 | 2.96558 | -0.26254 |
| H | -4.47517 | 1.81707 | -0.40952 |
| H | -5.29886 | 3.9799 | 0.56285 |
| H | -5.47541 | 2.68039 | 1.74658 |
| H | -4.14834 | 3.83924 | 1.89701 |
| H | 0.14178 | 3.47943 | -3.69991 |
| H | -1.54614 | 2.95248 | -3.79818 |
| H | -0.46929 | 5.0563 | -1.84792 |
| H | -2.14686 | 4.54319 | -1.96166 |
| H | -2.25712 | 5.41027 | -4.34094 |
| H | -1.80299 | 6.6919 | -3.21245 |
| H | -0.57112 | 5.93041 | -4.22505 |
| H | 1.99604 | 3.07402 | 1.98713 |
| H | 1.81707 | 3.70172 | 0.34231 |
| H | -0.66111 | 3.36871 | 0.51287 |
| H | -0.46026 | 2.80793 | 2.16899 |
| H | -1.1283 | 5.23971 | 2.15123 |
| H | 0.49711 | 5.07347 | 2.82254 |
| H | 0.27949 | 5.6368 | 1.16152 |
| H | 0.5248 | -0.14067 | 4.96123 |
| H | -1.125 | -0.77095 | 4.82965 |
| H | -2.00174 | 1.5716 | 4.68927 |
| H | -0.36392 | 2.19734 | 4.81986 |
| H | -1.79409 | 0.53326 | 6.99247 |
| H | -0.14684 | 1.16258 | 7.12375 |
| H | -1.51586 | 2.27727 | 7.05034 |
| H | -5.46619 | -5.79858 | -1.50988 |
| H | -4.03201 | -5.558 | -0.47754 |
| H | -5.59565 | -4.82362 | -0.02201 |
| H | 2.97127 | -2.06359 | -4.84661 |
| H | 3.02124 | -2.76844 | -3.20725 |
| H | 2.52082 | -3.77115 | -4.59579 |
| H | 8.11431 | -1.08565 | 0.04266 |
| H | 7.28046 | -0.28436 | 1.40097 |
| H | 6.67357 | -1.84171 | 0.77221 |
| H | 3.18511 | -4.02925 | -0.11553 |
| H | 2.84616 | -5.75728 | 0.17249 |
| H | 3.36637 | -4.71554 | 1.52336 |

**Tetrabromo 8 PC1**

| atom | X | Y | Z |
| --- | --- | --- | --- |
| C | 2.95266 | 1.8282 | 1.01482 |
| C | 1.92763 | 2.86919 | 1.43062 |
| C | 4.18134 | 2.20591 | 0.47898 |
| C | 2.66261 | 0.45661 | 1.08586 |
| C | 3.4788 | -0.51285 | 0.47155 |
| C | 4.72808 | -0.08607 | -0.00308 |
| C | -0.60996 | 2.88519 | 1.13302 |
| C | -1.76883 | 2.75968 | 0.33338 |
| C | -1.59762 | 2.81821 | -1.05281 |
| C | 0.79096 | 2.81045 | -0.821 |
| C | 0.6695 | 2.8576 | 0.568 |
| O | -0.72247 | 2.95746 | 2.51087 |
| O | 1.51552 | 0.03792 | 1.71051 |
| C | -4.7508 | 0.83792 | -0.11327 |
| C | -3.61058 | 1.04701 | 0.66593 |
| C | -2.87962 | -0.07068 | 1.08254 |
| C | -3.12324 | -1.35068 | 0.54334 |
| C | -4.25851 | -1.51102 | -0.25467 |
| O | -1.8487 | 0.08387 | 1.98191 |
| C | 0.44052 | -2.38969 | 0.36957 |
| C | -0.84965 | -2.19283 | -0.1292 |
| C | -0.99853 | -1.70873 | -1.43049 |
| C | 1.38464 | -1.56528 | -1.67677 |
| C | 1.58146 | -1.98939 | -0.36088 |
| O | 0.59592 | -2.91087 | 1.64435 |
| C | 2.95198 | -1.93802 | 0.31114 |
| C | -2.0792 | -2.43134 | 0.74614 |
| C | -3.12859 | 2.46509 | 0.9686 |
| C | 5.096 | 1.26369 | 0.01404 |
| C | 0.10262 | -1.42467 | -2.23073 |
| C | -0.32704 | 2.83094 | -1.64968 |
| C | -5.11662 | -0.436 | -0.54376 |
| O | 6.33852 | 1.68075 | -0.39556 |
| C | 6.43672 | 2.02766 | -1.77791 |
| O | -0.27325 | 2.84511 | -3.00594 |
| C | 0.99723 | 2.74566 | -3.63866 |
| O | 0.01988 | -0.98558 | -3.51419 |
| C | -1.26869 | -0.77788 | -4.07956 |
| O | -6.22847 | -0.70312 | -1.27845 |
| C | -7.10246 | 0.36562 | -1.60485 |
| C | 1.54575 | 0.04371 | 3.15069 |
| C | 2.45275 | -1.01117 | 3.77643 |
| C | 2.43819 | -0.9202 | 5.30534 |
| C | 0.99096 | -4.29353 | 1.70559 |
| C | -0.15149 | -5.27695 | 1.47272 |
| C | 0.31671 | -6.729 | 1.60458 |
| Br | -3.1012 | 2.93309 | -2.24685 |
| Br | -4.7107 | -3.22034 | -1.01364 |
| H | 4.46615 | 3.25301 | 0.43876 |
| C | -1.26358 | 4.16266 | 3.08682 |
| C | -0.22681 | 5.24262 | 3.39988 |
| C | 0.20569 | 6.10347 | 2.20954 |
| C | -2.2662 | 0.26456 | 3.34951 |
| C | -2.43389 | -1.0493 | 4.10638 |
| C | -2.81792 | -0.81273 | 5.56987 |
| Br | 2.87075 | -1.16077 | -2.82408 |
| H | 2.4016 | 3.85507 | 1.36748 |
| H | 1.6289 | 2.74566 | 2.46945 |
| Br | 6.04171 | -1.34407 | -0.62811 |
| H | 1.78192 | 2.76064 | -1.25303 |
| H | -5.33626 | 1.69549 | -0.4156 |
| H | -1.99562 | -1.54557 | -1.81503 |
| H | 2.87734 | -2.40641 | 1.29053 |
| H | 3.67308 | -2.51631 | -0.26565 |
| H | -2.51255 | -3.40965 | 0.5322 |
| H | -1.7536 | -2.44557 | 1.7825 |
| H | -3.87654 | 3.17357 | 0.60427 |
| H | -3.0518 | 2.61193 | 2.04358 |
| H | 7.46977 | 2.33136 | -1.94311 |
| H | 6.20128 | 1.17385 | -2.41982 |
| H | 5.76855 | 2.86122 | -2.02207 |
| H | 1.4993 | 1.81025 | -3.37803 |
| H | 1.63657 | 3.59835 | -3.38619 |
| H | 0.78943 | 2.75814 | -4.70685 |
| H | -1.851 | -1.70465 | -4.09866 |
| H | -1.82103 | -0.00317 | -3.54045 |
| H | -1.08672 | -0.44567 | -5.09991 |
| H | -7.92042 | -0.08631 | -2.16262 |
| H | -7.4998 | 0.84865 | -0.70564 |
| H | -6.60722 | 1.11409 | -2.2321 |
| H | 0.50867 | -0.12859 | 3.43106 |
| H | 1.83245 | 1.03739 | 3.51243 |
| H | 3.47738 | -0.8819 | 3.41246 |
| H | 2.11265 | -1.9966 | 3.45288 |
| H | 2.80152 | 0.05096 | 5.65619 |
| H | 3.07604 | -1.68724 | 5.75158 |
| H | 1.42952 | -1.05936 | 5.70769 |
| H | 1.40235 | -4.42282 | 2.7108 |
| H | 1.79918 | -4.47772 | 0.9876 |
| H | -0.57373 | -5.11334 | 0.47648 |
| H | -0.94968 | -5.072 | 2.19331 |
| H | -0.5104 | -7.4246 | 1.44409 |
| H | 1.0933 | -6.96925 | 0.87199 |
| H | 0.72735 | -6.93172 | 2.59883 |
| H | -1.73592 | 3.82948 | 4.01456 |
| H | -2.04701 | 4.56815 | 2.4372 |
| H | 0.63965 | 4.76771 | 3.87204 |
| H | -0.66651 | 5.89366 | 4.16624 |
| H | 0.66467 | 5.51288 | 1.41573 |
| H | 0.92843 | 6.86296 | 2.52029 |
| H | -0.65068 | 6.6257 | 1.77172 |
| H | -1.49287 | 0.88551 | 3.80256 |
| H | -3.2048 | 0.82988 | 3.37672 |
| H | -3.20189 | -1.65692 | 3.61652 |
| H | -1.499 | -1.61589 | 4.04833 |
| H | -2.92645 | -1.75785 | 6.10737 |
| H | -2.05992 | -0.2224 | 6.09461 |
| H | -3.76883 | -0.27741 | 5.65508 |

**Tetrabromo PC2 8**

| atom | X | Y | Z |
| --- | --- | --- | --- |
| C | -3.88895 | 0.30403 | 0.33518 |
| C | -3.6725 | -0.90105 | 1.23842 |
| C | -4.83049 | 0.33624 | -0.69625 |
| C | -3.00969 | 1.40107 | 0.45399 |
| C | -2.93774 | 2.38721 | -0.53238 |
| C | -3.88849 | 2.37527 | -1.55717 |
| C | -1.34274 | -1.89405 | 1.62844 |
| C | -0.15544 | -2.49303 | 1.14262 |
| C | -0.19817 | -3.06173 | -0.13353 |
| C | -2.42304 | -2.23583 | -0.49376 |
| C | -2.45104 | -1.7029 | 0.79644 |
| O | -1.40879 | -1.42695 | 2.92916 |
| O | -2.14265 | 1.44911 | 1.52086 |
| C | 3.51699 | -2.34999 | 1.22772 |
| C | 2.29678 | -1.70498 | 1.44428 |
| C | 2.21474 | -0.33662 | 1.16655 |
| C | 3.25977 | 0.3473 | 0.50552 |
| C | 4.47335 | -0.32685 | 0.34774 |
| O | 1.08794 | 0.377 | 1.50422 |
| C | 0.692 | 2.69398 | -0.4478 |
| C | 1.7865 | 1.91765 | -0.88438 |
| C | 1.66954 | 1.28902 | -2.13077 |
| C | -0.5956 | 2.04301 | -2.36216 |
| C | -0.5263 | 2.71151 | -1.14199 |
| O | 0.81272 | 3.42063 | 0.72471 |
| C | -1.77919 | 3.36643 | -0.56149 |
| C | 3.02426 | 1.76574 | -0.00834 |
| C | 1.11624 | -2.51064 | 1.98677 |
| C | -4.86922 | 1.3867 | -1.63017 |
| C | 0.49235 | 1.35854 | -2.88369 |
| C | -1.32715 | -2.95145 | -0.96072 |
| C | 4.6238 | -1.67161 | 0.72623 |
| O | -5.84767 | 1.3497 | -2.57179 |
| C | -5.91104 | 2.39222 | -3.53232 |
| O | -1.26987 | -3.5389 | -2.1867 |
| C | -2.45291 | -3.58692 | -2.97028 |
| O | 0.39575 | 0.78689 | -4.12765 |
| C | -0.08913 | -0.55922 | -4.13216 |
| O | 5.84713 | -2.23849 | 0.54572 |
| C | 6.02397 | -3.60113 | 0.89311 |
| C | -2.72615 | 1.83272 | 2.78166 |
| C | -2.53344 | 3.31464 | 3.08486 |
| C | -3.06068 | 3.68581 | 4.47367 |
| C | 0.95497 | 4.84372 | 0.56014 |
| C | 2.36627 | 5.28387 | 0.18286 |
| C | 2.46352 | 6.8049 | 0.03501 |
| Br | 1.27232 | -4.08652 | -0.81998 |
| Br | 6.03356 | 0.55081 | -0.35237 |
| Br | -6.13595 | -1.06444 | -0.90897 |
| C | -1.39755 | -2.39094 | 4.00181 |
| C | -2.77454 | -2.63225 | 4.61786 |
| C | -3.69259 | -3.55895 | 3.81742 |
| C | 0.97123 | 0.72508 | 2.89773 |
| C | 1.99745 | 1.73409 | 3.40541 |
| C | 1.80686 | 2.00922 | 4.90035 |
| H | -1.51523 | 2.03886 | -2.93672 |
| H | -4.56043 | -1.53374 | 1.21538 |
| H | -3.52914 | -0.58797 | 2.26841 |
| H | -3.85573 | 3.1589 | -2.30414 |
| H | -3.2848 | -2.0939 | -1.13214 |
| H | 3.58902 | -3.40704 | 1.44785 |
| Br | 3.14984 | 0.3556 | -2.91208 |
| H | -1.55755 | 3.71111 | 0.44502 |
| H | -2.06128 | 4.24296 | -1.15533 |
| H | 3.90368 | 2.08132 | -0.56888 |
| H | 2.92692 | 2.44538 | 0.83512 |
| H | 1.4497 | -3.54556 | 2.10474 |
| H | 0.87328 | -2.15589 | 2.98565 |
| H | -6.77492 | 2.16457 | -4.15359 |
| H | -6.05308 | 3.36845 | -3.05687 |
| H | -5.0129 | 2.41854 | -4.15864 |
| H | -2.77694 | -2.58772 | -3.27896 |
| H | -3.2687 | -4.08019 | -2.43219 |
| H | -2.19507 | -4.16877 | -3.85326 |
| H | 0.55443 | -1.22017 | -3.54654 |
| H | -1.10922 | -0.60466 | -3.73564 |
| H | -0.0874 | -0.87854 | -5.17389 |
| H | 7.06721 | -3.82458 | 0.67765 |
| H | 5.82872 | -3.7747 | 1.9572 |
| H | 5.3831 | -4.25696 | 0.29421 |
| H | -2.23305 | 1.21445 | 3.53505 |
| H | -3.79276 | 1.58297 | 2.78868 |
| H | -3.04811 | 3.90933 | 2.32254 |
| H | -1.46785 | 3.55158 | 3.00541 |
| H | -4.13195 | 3.48147 | 4.56609 |
| H | -2.91186 | 4.74818 | 4.6816 |
| H | -2.54941 | 3.12328 | 5.26117 |
| H | 0.66933 | 5.2667 | 1.52716 |
| H | 0.2373 | 5.20338 | -0.18633 |
| H | 2.65895 | 4.80063 | -0.75454 |
| H | 3.06748 | 4.93707 | 0.94855 |
| H | 3.48039 | 7.10986 | -0.22273 |
| H | 1.80101 | 7.17564 | -0.75325 |
| H | 2.19259 | 7.3188 | 0.96285 |
| H | -0.72683 | -1.97478 | 4.75942 |
| H | -0.96725 | -3.33458 | 3.6517 |
| H | -3.2547 | -1.66226 | 4.78672 |
| H | -2.61017 | -3.06379 | 5.61351 |
| H | -3.89867 | -3.17212 | 2.81885 |
| H | -4.652 | -3.69232 | 4.32459 |
| H | -3.24341 | -4.54974 | 3.69799 |
| H | -0.03639 | 1.12453 | 2.97622 |
| H | 1.01736 | -0.18242 | 3.50872 |
| H | 3.00999 | 1.35549 | 3.23153 |
| H | 1.89213 | 2.65886 | 2.83566 |
| H | 2.52976 | 2.74491 | 5.26211 |
| H | 0.807 | 2.40155 | 5.11376 |
| H | 1.93794 | 1.10188 | 5.4989 |

**11 References**

[S1] G. R. Fulmer, A. J. M. Miller, N. H. Sherden, H. E. Gottlieb, A. Nudelman, B. M. Stoltz, J. E. Bercaw, K. I. Goldberg, *Organometallics* **2010**, *29*, 2176-2179.

[S2] L. Krause, R. Herbst-Irmer, G. M. Sheldrick, D. Stalke, *J. Appl. Crystallogr.* **2015**, *48*, 3-10.

[S3] G. M. Sheldrick, *Acta Crystallogr., Sect. A:Found. Adv.* **2015**, *71*, 3-8.

[S4] G. M. Sheldrick, *Acta Crystallogr., Sect. C:Struct. Chem.* **2015**, *71*, 3-8.

[S5] M. J. Frisch, G. W. Trucks, H. B. Schlegel, G. E. Scuseria, M. A. Robb, J. R. Cheeseman, G. Scalmani, V. Barone, G. A. Petersson, H. Nakatsuji, X. Li, M. Caricato, A. V. Marenich, J. Bloino, B. G. Janesko, R. Gomperts, B. Mennucci, H. P. Hratchian, J. V. Ortiz, A. F. Izmaylov, J. L. Sonnenberg, Williams, F. Ding, F. Lipparini, F. Egidi, J. Goings, B. Peng, A. Petrone, T. Henderson, D. Ranasinghe, V. G. Zakrzewski, J. Gao, N. Rega, G. Zheng, W. Liang, M. Hada, M. Ehara, K. Toyota, R. Fukuda, J. Hasegawa, M. Ishida, T. Nakajima, Y. Honda, O. Kitao, H. Nakai, T. Vreven, K. Throssell, J. A. Montgomery Jr., J. E. Peralta, F. Ogliaro, M. J. Bearpark, J. J. Heyd, E. N. Brothers, K. N. Kudin, V. N. Staroverov, T. A. Keith, R. Kobayashi, J. Normand, K. Raghavachari, A. P. Rendell, J. C. Burant, S. S. Iyengar, J. Tomasi, M. Cossi, J. M. Millam, M. Klene, C. Adamo, R. Cammi, J. W. Ochterski, R. L. Martin, K. Morokuma, O. Farkas, J. B. Foresman, D. J. Fox, Wallingford, CT, **2016**.

[S6] P. Hohenberg, W. Kohn, *Phys. Rev.* **1964**, *136*, B864-B871.

[S7] W. Kohn, L. J. Sham, *Phys. Rev.* **1965**, *140*, 1133-1138.

[S8] R. G. P. a. W.Yang, *Density-functional theory of atoms and molecules*, Oxford Univ. Press New York, NY, **1994**.

[S9] D. M. C. H. Prof. Dr. Wolfram Koch, *A Chemist's Guide to Density Functional Theory*, Wiley‐VCH Verlag GmbH, **2001**.

[S10] S. H. Vosko, L. Wilk, M. Nusair, *Can. J. Phys.* **1980**, *58*, 1200-1211.

[S11] C. Lee, W. Yang, R. G. Parr, *Phys. Rev. B* **1988**, *37*, 785-789.

[S12] A. D. Becke, *J. Chem. Phys.* **1993**, *98*, 5648-5652.

[S13] P. J. Stephens, F. J. Devlin, C. F. Chabalowski, M. J. Frisch, *J. Phys. Chem.* **1994**, *98*, 11623-11627.

[S14] R. Krishnan, J. S. Binkley, R. Seeger, J. A. Pople, *J. Chem. Phys* **1980**, *72*, 650-654.

[S15] A. D. McLean, G. S. Chandler, *J. Chem. Phys* **1980**, *72*, 5639-5648.

[S16] M. M. Francl, W. J. Pietro, W. J. Hehre, J. S. Binkley, M. S. Gordon, D. J. DeFrees, J. A. Pople, *J. Chem. Phys.* **1982**, *77*, 3654-3665.

[S17] M. N. Glukhovtsev, A. Pross, M. P. McGrath, L. Radom, *J. Chem. Phys.* **1995**, *103*, 1878-1885.

[S18] J. J. P. Stewart, *J. Mol. Model.* **2007**, *13*, 1173-1213.

[S19] J. A. Gaunt, *Mathematical Proceedings of the Cambridge Philosophical Society* **1928**, *24*, 328-342.

[S20] D. R. Hartree, *Mathematical Proceedings of the Cambridge Philosophical Society* **1928**, *24*, 111-132.

[S21] J. C. Slater, *Physical Review* **1928**, *32*, 339-348.

[S22] V. Fock, *Zeitschrift für Physik* **1930**, *62*, 795-805.

[S23] J. C. Slater, *Physical Review* **1930**, *35*, 210-211.

[S24] C. C. J. Roothaan, *Reviews of Modern Physics* **1951**, *23*, 69-89.

[25] D. R. Hartree, W. Hartree, *Proceedings of the Royal Society of London. Series A, Mathematical and Physical Sciences* **1997**, *150*, 9-33.

[S26] F. London, *Journal de Physique et le Radium* **1937**, *8*, 397-409.

[S27] R. McWeeny, *Phys. Rev.* **1962**, *126*, 1028-1034.

[S28] R. Ditchfield, *Mol. Phys.* **1974**, *27*, 789-807.

[S29] K. Wolinski, J. F. Hinton, P. Pulay, *Journal of the American Chemical Society* **1990**, *112*, 8251-8260.

[S30] J. R. Cheeseman, G. W. Trucks, T. A. Keith, M. J. Frisch, *J. Chem. Phys.* **1996**, *104*, 5497-5509.

[S31] B. G. Johnson, P. M. W. Gill, J. A. Pople, D. J. Fox, *Chem. Phys. Lett.* **1993**, *206*, 239-246.

[S32] D. P. Chong, *Recent Advances in Density Functional Methods, Part I*, World Scientific Publishing Co Pte Ltd, World Scientific Publishing Co Pte Ltd, **1996**.

[S33] B. D. C. David Gutsche, Kwang Hyun No, and Ramamurthi Muthukrishnan, *J. Am. Chem. Soc.* **1981**, *103*, 3782-3792.

[S34] C. David Gutsche, L.-G. Lin, *Tetrahedron* **1986**, *42*, 1633-1640.

[S35] L. M. Arturo Arduini, Daniela Paganuzzi, Alessandra Pinalli, Andrea Pochini, Andrea Secchi, Rocco Ungaro, *Tetrahedron* **1996**, *52*, 6011-6018.

[S36] A. M. Alessandro Dondoni, Marie-Christine Scherrmann, Alessandro Casnati, Francesco Sansone, and Rocco Ungaro, *Chem. - Eur. J.* **1997**, *3*.

[S37] A. A. P. Vyacheslav I. Boyko, Anton V. Yakovenko, Vladimir, V. P. a. V. I. Kalchenko, *J. Inclusion Phenom. Macrocyclic Chem.* **2005**, *50*, 193-197.

[S38] V. J. B. Frank A. L. Anet, *Journal of Magnetic Resonance* **1978**, *32*, 339-343.

[S39] F. P. Gasparro, N. H. Kolodny, *J. Chem. Educ.* **1977**, *54*, 258.

[S40] T. Galaon, V. David, *J. Sep. Sci.* **2011**, *34*, 1423-1428.

[S41] J. W. Ochterski, *Gaussian Inc.* **2000**.

[S42] S. P. H. Donald J. Cram, *J. Am. Chem. Soc.* **1986**, *108*, 2998-3005.

[S43] G. D. B. a. J. F. Stoddart, *Tetrahedron Lett.* **1980**, *21*, 867 - 870.

[S44] P. Thordarson, *Chem. Soc. Rev.* **2011**, *40*, 1305-1323.

[S45] D. Brynn Hibbert, P. Thordarson, *Chem. Commun.* **2016**, *52*, 12792-12805.

Links for Bindfit: Nanocone **3** and LiOTf in THf-d_8_:

E1M1: http://app.supramolecular.org/bindfit/view/d1d4303c-e5a

E1M2: http://app.supramolecular.org/bindfit/view/563bbab9-8246-431c-97e0-7ef52fceb42c

E1M3: http://app.supramolecular.org/bindfit/view/776a5f04-1de7-4b06-bb57-e07daa60c3b2

E2M1: http://app.supramolecular.org/bindfit/view/f2fd7d7d-0375-468e-a8e0-4139a23c1e52

E2M2: http://app.supramolecular.org/bindfit/view/69c4ab27-eeda-4cc7-8ddb-7ba3ee9d45c4

E2M3: http://app.supramolecular.org/bindfit/view/3c575524-04d2-4bb3-8b44-0263aabafda2

Reference for the plotting with the initial Nanocone **3** concentrations (titration 1: 4.31·10^-3^ M^-1^, titration 2: 3.78·10^-3^ M^-1^)

http://app.supramolecular.org/bindfit/view/9ff81b41-ca6d-464f-a2f4-a8fc6637f759

http://app.supramolecular.org/bindfit/view/4f18918e-eabf-464c-86de-c0e5d74a8f7e

http://app.supramolecular.org/bindfit/view/3f3c8ac8-3a42-44c8-9624-b2a35ead411c

http://app.supramolecular.org/bindfit/view/44c0e6b2-d3b9-4732-b09d-c9a12c0fb860

http://app.supramolecular.org/bindfit/view/bafcc52e-08d2-4e3b-a699-972ae331d148
